# Inhibition of H1 and H5 Influenza A Virus Entry by Diverse Macrocyclic Peptides Targeting the Hemagglutinin Stem Region

**DOI:** 10.1021/acschembio.2c00040

**Published:** 2022-08-04

**Authors:** Mirte N. Pascha, Vito Thijssen, Julia E. Egido, Mirte W. Linthorst, Jipke H. van Lanen, David A. A. van Dongen, Antonius J. P. Hopstaken, Frank J. M. van Kuppeveld, Joost Snijder, Cornelis A. M. de Haan, Seino A. K. Jongkees

**Affiliations:** † Section Virology, Division Infectious Diseases and Immunology, Department of Biomolecular Health Sciences, Faculty of Veterinary Medicine, Utrecht University, Yalelaan 1, 3584 CL Utrecht, The Netherlands; ‡ Department of Chemical Biology & Drug Discovery, Utrecht Institute for Pharmaceutical Sciences, Utrecht University, Universiteitsweg 99, 3584 CG Utrecht, The Netherlands; § Biomolecular Mass Spectrometry and Proteomics, Bijvoet Center for Biomolecular Research and Utrecht Institute for Pharmaceutical Sciences, Utrecht University, Padualaan 8, 3584 CH Utrecht, The Netherlands; ∥ Department of Chemistry and Pharmaceutical Sciences, Amsterdam Institute for Molecular and Life Sciences, VU Amsterdam, de Boelelaan 1108, 1081 HZ Amsterdam, The Netherlands

## Abstract

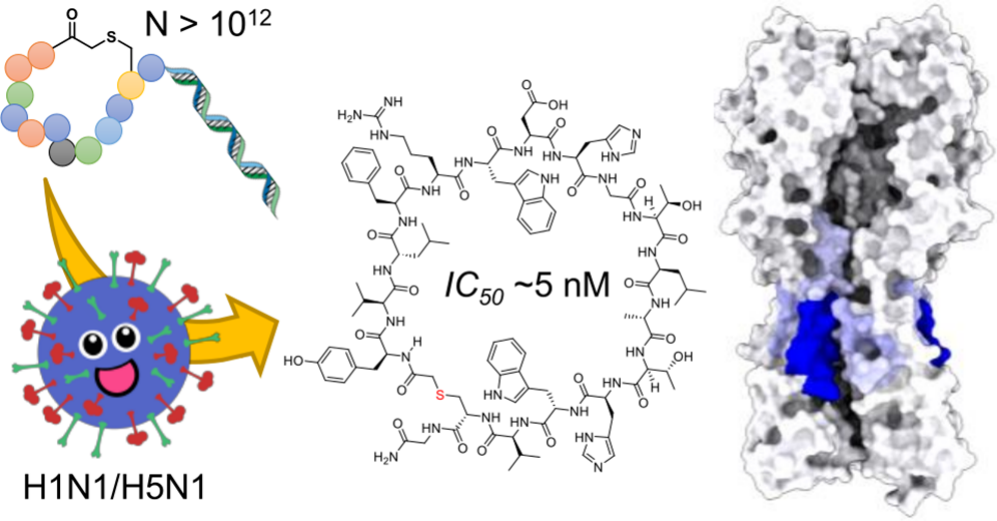

Influenza A viruses pose a serious pandemic risk, while generation of efficient vaccines against seasonal variants remains challenging. There is thus a pressing need for new treatment options. We report here a set of macrocyclic peptides that inhibit influenza A virus infection at low nanomolar concentrations by binding to hemagglutinin, selected using ultrahigh-throughput screening of a diverse peptide library. The peptides are active against both H1 and H5 variants, with no detectable cytotoxicity. Despite the high sequence diversity across hits, all tested peptides were found to bind to the same region in the hemagglutinin stem by HDX-MS epitope mapping. A mutation in this region identified in an escape variant confirmed the binding site. This stands in contrast to the immunodominance of the head region for antibody binding and suggests that macrocyclic peptides from in vitro display may be well suited for finding new druggable sites not revealed by antibodies. Functional analysis indicates that these peptides stabilize the prefusion conformation of the protein and thereby prevent virus–cell fusion. High-throughput screening of macrocyclic peptides is thus shown here to be a powerful method for the discovery of novel broadly acting viral fusion inhibitors with therapeutic potential.

## Introduction

Influenza A virus (IAV) has caused numerous pandemics throughout history and remains a great risk for causing pandemics in the future. Current vaccines against seasonal variants are typically strain-specific, and the antigenic mismatch between vaccine antigens and the circulating strains limits their efficacy. An emerging antigenically novel virus will require a vaccine update, or the virus will be able to spread without much constraint.^1^ In the event of a new pandemic, broadly acting therapies would therefore be essential to bridge the time needed to produce an antigenically matched vaccine.

The IAV envelope contains two glycoproteins: hemagglutinin (HA) and neuraminidase (NA). Both have essential roles in the viral replication cycle: HA for binding to the receptor and mediating membrane fusion, and NA for the release of virus particles from decoy receptors in mucus or on the cell surface.^2,3^ Current therapeutic strategies against IAV are predominantly focused on NA.^4^ Small-molecule inhibitors of NA target the conserved catalytic site and as a result are broadly cross-protective.

Attempts to target hemagglutinin (HA) are however on the rise.^5^ The discovery of broadly neutralizing antibodies that target the HA stem^6^ has led to increased attention for HA as a target for broadly cross-protective antivirals. These antibodies are under investigation for direct use as therapeutic agents and have served as inspiration for the design of smaller cyclic peptides based on the antibody-binding interface.^7−11^ However, as is seen with neuraminidase-targeting drugs, the intrinsic high mutation rate of IAV can cause the virus to develop resistance^12^ and so a single entity is liable to be relatively easily overcome.

One strategy to minimize escape is demonstrated in combination antiretroviral therapy: coadministration of multiple antiviral drugs with different targets. In a similar strategy, we envisaged the discovery of a suite of broadly reactive antiviral peptides with varied structures and binding various sites on HA that could be coadministered, either as a mixture or a fused single entity, to minimize viral escape. Instead of rational design such as the antibody-based method mentioned earlier, we opted for an unbiased peptide display technology to potentially find new-to-nature sequences using a platform called the RaPID system.^13^ This is a combination of ultrahigh-throughput screening of peptides by mRNA display with translation under a reprogrammed genetic code to allow the display of stably macrocyclized structures. This technology has proven successful in providing peptide-based hits against a diverse array of target types, including soluble,^14^ membrane-associated,^15^ and integral membrane proteins.^16^ RaPID display is entirely carried out *in vitro*, unlike techniques such as phage display. Therefore it is highly amenable to modifications in both library architecture and building block composition while also reducing potential sequence biases. The genetic code reprogramming employed in the RaPID system is also particularly flexible as it does not require the use of aminoacyl-tRNA synthetases. Noncanonical amino acids are instead activated synthetically and then charged onto in vitro transcribed tRNA by the use of a catalytic acylating ribozyme termed a flexizyme.^17^ The most common application of this reprogramming is to initiate peptide synthesis with a chloroacetylated amino acid, which reacts spontaneously with the first downstream cysteine. The head-to-sidechain thioether macrocyclization that results is readily formed in both translated and synthetic peptides and is not susceptible to reductive opening.^18^ Such macrocyclic peptides have several advantages over their linear counterparts. They are more resistant to proteolytic degradation by both endo- and exo-acting proteases, and the more limited conformational flexibility removes some of the entropic cost of binding and reduces the likelihood of off-target binding by alternate conformations.^19^

In this work, we use the RaPID system for the mRNA display of macrocyclic peptides to derive HA-binding peptides as potential broadly acting anti-influenza agents. Another recent report also details the use of the RaPID system to target HA.^20^ In our work, we target H1 instead of H5 and perform additional subselections to directly target the HA stem region. Our selection strategy proved successful, affording HA-binding peptides with notably high affinities and slow dissociation rates. All peptides tested proved to be potent inhibitors of IAV infection with IC_50_ values as low as 6 nM for H1 subtype viruses. One candidate showed particularly broad activity and was equally effective in the inhibition of infection by H5 subtype viruses. Hydrogen–deuterium exchange mass spectrometry (HDX-MS) demonstrated that the inhibiting peptides all bind the same helix A in the HA stem, which was confirmed by the generation of a resistant virus. The peptides were shown to inhibit infection by preventing conformational changes in HA that are required for membrane fusion. We believe that this approach provides a promising new avenue for the treatment of IAV infection, including against pandemic strains not captured by seasonal vaccinations. These peptide inhibitors combine the benefits of broadly neutralizing activity with defined chemical entities that are amenable to mass production.

## Results and Discussion

**Figure 1:**
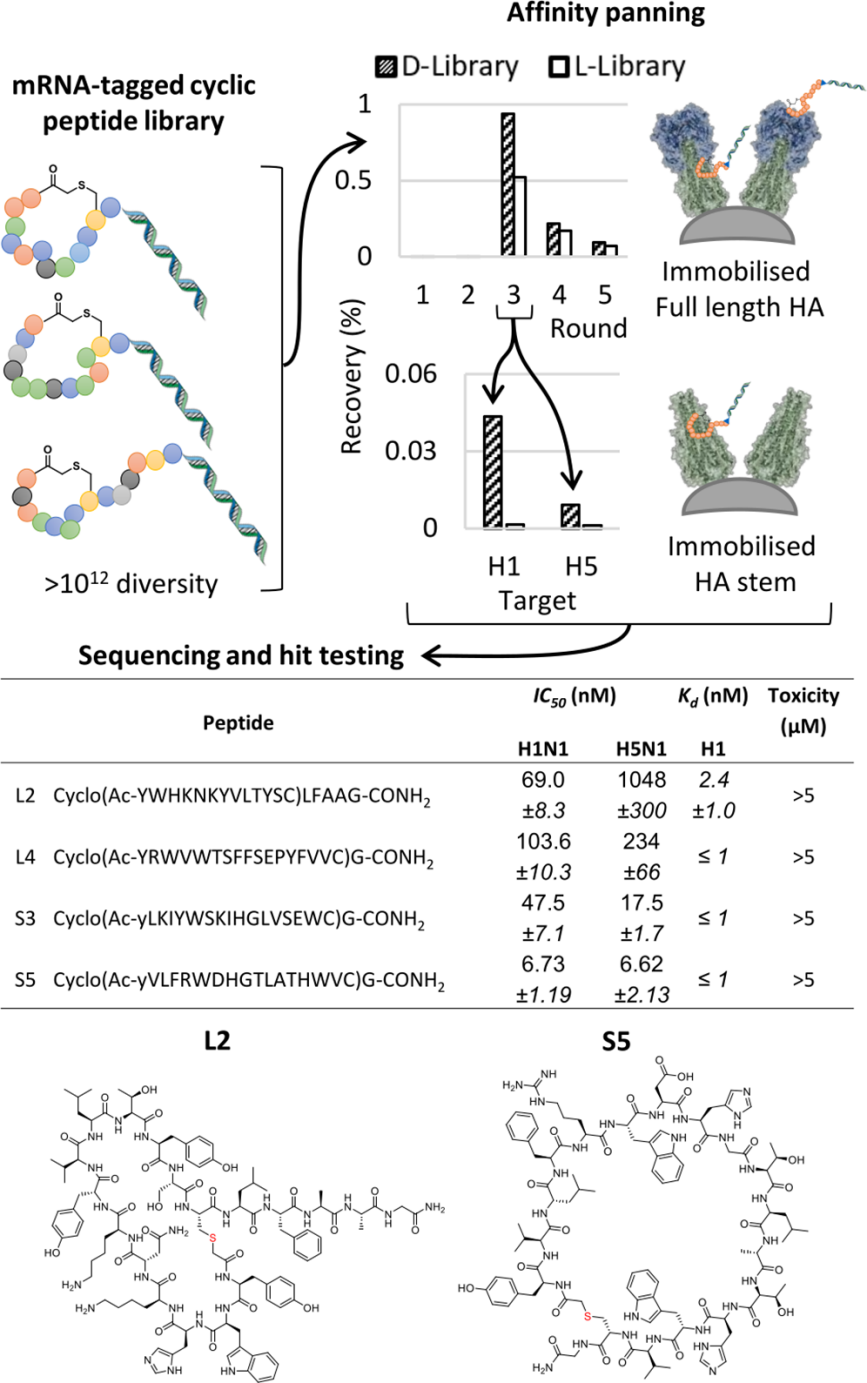
Schematic of the peptide selection process and summary of the four best-characterized resulting hits. Generation of an mRNA-displayed library of macrocyclic peptides by translation under a reprogrammed genetic code (upper left) allows this to be panned across immobilized HA (full-length or stem-only) for multiple rounds to enrich tight binders (upper right) that are subsequently identified by high-throughput sequencing and synthesized on solid phase for characterization, with example structures shown (lower). IC_50_ values were determined in a luciferase-based infection assay, *K*_D_ values by FP, and toxicity with a Wst-1 proliferation assay using HeLa and MDCKI cells.

Recombinant soluble trimeric HA ectodomain^21,22^ or HA stem-only proteins^23^ were purified as fusions with monomeric Fc^24^ and/or Strep tags (Table S1). Protein immobilization in these selections was alternated between the two tags to minimize the enrichment of peptides binding to either immobilization medium, starting with the Fc tag in the first round (Table S2). The first rounds of RaPID selections (Figures 1 and S1) were carried out on the full-size H1 ectodomain of a new pandemic H1N1 virus of 2009 (A/California/04/2009). The selection used a library of peptides of general architecture ClAc-Y-X_15_-C-G-S-G-S-G-S (where ClAc is a chloroacetyl group and X is any amino acid encoded by an NNK codon) following published protocols,^25^ with the initiating tyrosine in either d- or l-stereochemistry in two parallel selections. A strong enrichment of HA binders was already observed after three rounds (Figure S2a,b). Based on these results it was decided to extend the wash times for the final two rounds using the complete H1 ectodomain aiming to drive selection for peptides with tight binding through slow off-rates, and a decrease in recovery is observed as a result.

Following successful enrichment of the library for H1 binding peptides, we turned our attention to increasing the probability of finding broadly active sequences. To achieve this, we used the stem-only constructs from both H1 and H5 to further enrich our library. These constructs were used in a single round of selection each starting with the input library of the third round of our initial full-length H1 selection. These rounds resulted in a dramatically lower recovery than their counterpart round in the main selection, but nonetheless, especially the d-tyrosine-initiated library showed substantial recovery over the background. Because of the limited diversity of this input library, we opted not to carry out further selection rounds. All enriched libraries were subsequently submitted for high-throughput sequencing on the Illumina MiSeq platform, resulting in approximately 10–100 thousand sequences per library. Unique peptide sequences were extracted and tallied (enrichment of sequences across rounds shown in Figure S2c–f) and the most abundant of these were then analyzed by multiple sequence alignment (Figures S3–S6). Representative members of each sequence family were chosen for further analysis based on sequence abundance while additionally aiming to cover as broad a sequence space as possible within the potential hits. Particular focus was also placed on choosing peptides that were represented at relatively high abundance in several enriched libraries, thereby aiming to increase the chances of finding sequences binding to conserved regions. Overall, the hits were highly converged but still surprisingly diverse, with a number of seemingly unrelated sequence families. In the final round of selection, the top 20 sequences accounted for 44 and 51% of all sequences (l- and d-Tyr-initiated, respectively). The l-Tyr-initiated library showed no recognizable sequence similarities between 15 of these 20, while for d-Tyr, the sequences were more conserved (Figures S3 and S4, respectively). Between the libraries initiated with d- and l-tyrosine, there is little overlap, with each initiator giving a unique pool of hits. The selections against the H1 and H5 stem domains each showed a set of sequences distinct from the pool observed with the whole H1 ectodomain protein, but with substantial overlap between them (Figures S5 and S6 vs S4). Overall, no single interaction motif could be identified, although aromatic and positively charged residues did appear to be enriched in the hits. Many hits also contained a single negatively charged residue. Most hits from the l-tyrosine-initiated library were a macrocycle spanning the full 15 amino acids in the random region of the original library, although a few lariat peptides of intermediate macrocycle size were also found (e.g., L2), while the d-tyrosine-initiated library showed many hits with a four-residue macrocycle of sequence Y-F-L/V-C, followed by a long linear *C*-terminal region that again contained diverse sequences. This pattern did not hold for the sequences selected from the same library against the stem-only constructs, where full-length macrocycles again dominated.

To quickly screen the candidates identified in this sequence analysis, a small batch of each was produced by automated parallel Fmoc solid-phase peptide synthesis (SPPS) as C-terminal amides truncated after the first glycine in the spacer GSGSGS sequence (Figures S39–S59). A protein thermal shift assay was then used as a convenient preliminary screen for HA-binding ligands to find sequences of potential interest for an in-depth study. The assay detects a change in the thermal stability of a protein upon ligand binding. A set of 20 peptides were tested in the crude form in a thermal shift assay against H1 (Table S3, Figures S7 and S8), and a further set of three peptides (the top hits from the l-Tyr-initiated library) were also immediately purified and assessed using the same assay. In the case of the crude peptide, the percentage purity of the desired peptide in the crude mixture was estimated via HPLC-MS as the peptide yield can vary significantly during SPPS. Given that some were highly impure, an excess amount of peptide (estimated at 5 μM total) over H1 HA (200 nM) was used to ensure the presence of enough peptide to elicit a shift for all binding peptides. Note that impurities in this case will be truncated or slightly modified peptides of the same sequence, some of which may also bind, so this should not stop the assay from giving an indication of promising sequences. For two selected examples (two promising stem binders; d-Tyr-initiated), we also directly compared the peptides in both crude and purified forms to validate that these give a similar shift (Figure S7a). Almost all tested peptides were able to increase the thermal stability of H1 HA, although some displayed a greater increase than others. Together these confirmed that the majority of the sequences synthesized were at least able to bind to HA and therefore were suitable for follow-up assays.

**Figure 2:**
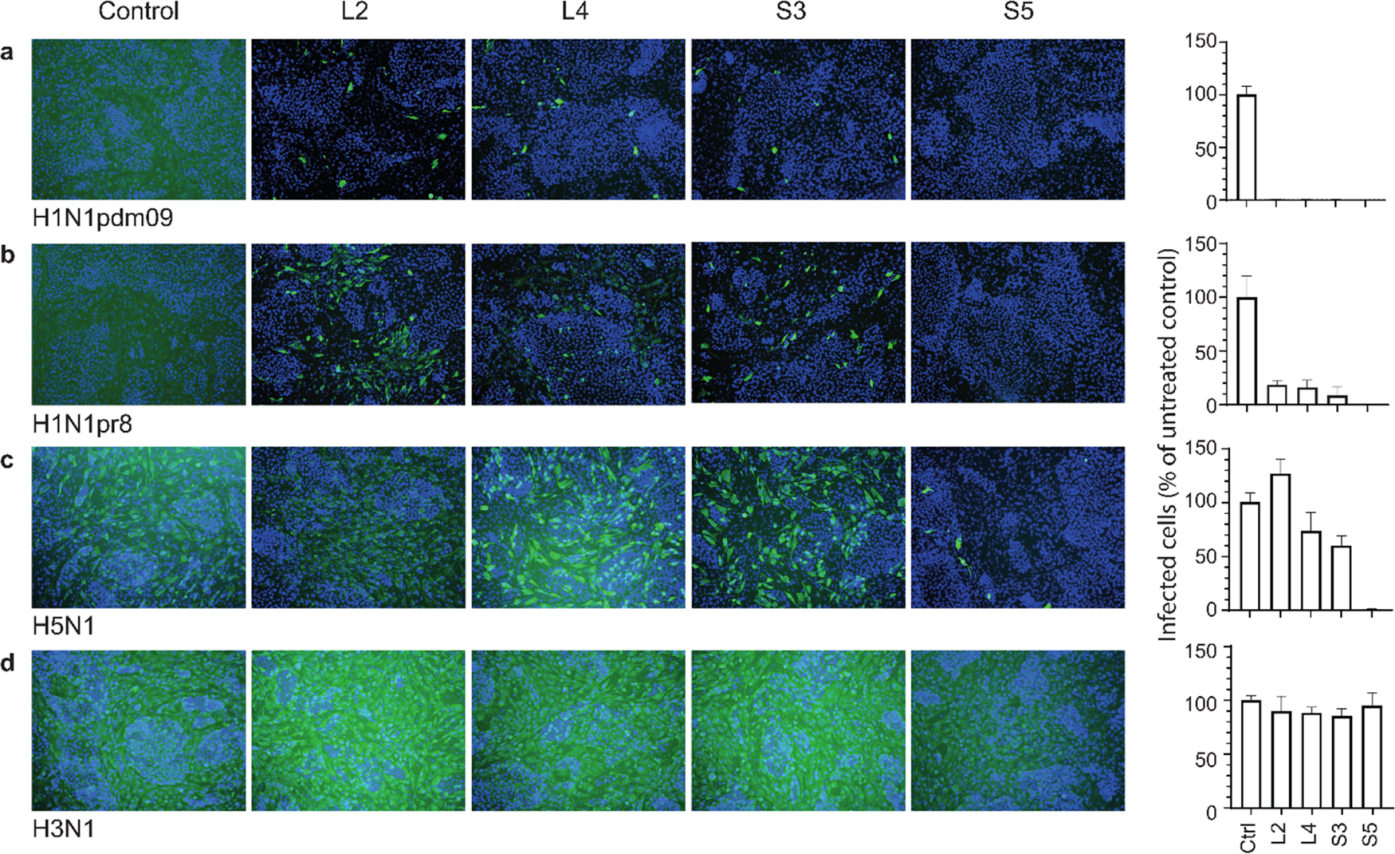
Peptides inhibit infection with H1N1 and H5N1 viruses. MDCKI cells were infected with H1N1pdm09 (A/Netherlands/602/2009) (a), H1N1pr8 (A/PR8/8/34/Mount Sinai) (b), H5N1 virus carrying H5 HA of A/duck/Hunan/795/2002 in the H1N1pr8 genetic background (c), and H3N1 virus carrying H3 HA of A/Bilthoven/1761/76 (H3N2) in the H1N1pr8 background (d) in the presence of macrocyclic peptides L2, L4, S3, and S5 at 5 μM. Left: representative images of immunofluorescence staining of the IAV nucleoprotein (green). Nuclei are labeled with DAPI (blue). Right: quantification of infected cells relative to untreated control. Data are shown as mean ± SD (*n* = 3).

Based on these indications of peptide binding to H1 HA, we proceeded to test a smaller set of purified peptides at a single concentration in a virus neutralization assay based on luciferase reporter gene expression.^26^ Cells transfected with the reporter plasmid express luciferase upon infection with IAV. (Figure S9a, peptides coded B1, B2, B4, L1, L2, L4, S2, S3, and S5). These peptides were prioritized based on the abundance rank in the sequencing data (lower number in the sequence code is higher ranked, described in more detail in the Supporting Information) and thermal shift data (excluding sequences that showed no shift) while preferring larger macrocycles as these are likely to be more stable in a biological setting than lariat peptides with a linear tail. Further active sequences are likely able to still be found in the data set, and for this reason, we provide the full raw sequencing data set as supporting material. All peptides tested displayed strong inhibition of infection with an H1N1pdm09 virus (A/Netherlands/602/2009). Four peptides were chosen for further characterization (coded L2, L4, S3, and S5), again based on their abundance in the sequencing results, the magnitude of the thermal shift, and their apparent initial neutralizing potency in the infection assay. All four candidates strongly inhibited infection (Figure 2) with both tested H1N1 strains (H1N1pdm09 and A/PR8/8/34/Mount Sinai [the latter is referred to as H1N1pr8]). Encouragingly, cross-neutralization was observed against a virus containing the HA of an H5N1 virus (A/duck/Hunan/795/2002) in the genetic background of H1N1pr8 (referred to as H5N1). This effect was particularly evident for S5, although the other peptides were also able to neutralize the H5N1 virus in a luciferase assay (Figure S9b). All peptides however fell short of inhibiting a virus carrying the HA gene of an H3N2 virus (A/Bilthoven/1761/76) in the background of H1N1pr8 (referred to as H3N1, Figure 2). The neutralizing potency of our peptides was quantified in the form of a half-maximal inhibitor concentration (IC_50_) from luciferase assays (Figure S9b). These results indicated S5 as not only the peptide with the broadest reactivity but also the one that exhibits the most potent neutralization with an IC_50_ of 6.7 nM against H1N1pdm09 and 6.6 nM against H5N1 (Figure 1). Control peptides with a scrambled order of the amino acids were not active against H1N1pdm09 and H5N1, while linear versions showed strongly reduced activity. Neutralizing peptides L2, L4, S3, and S5 was also shown to not be cytotoxic at concentrations up to 5 μM, and the neutralizing activity of S5 against H1N1pdm09 was unaffected by the addition of serum proteins (Figure S10), indicating sufficient protease stability.

**Figure 3:**
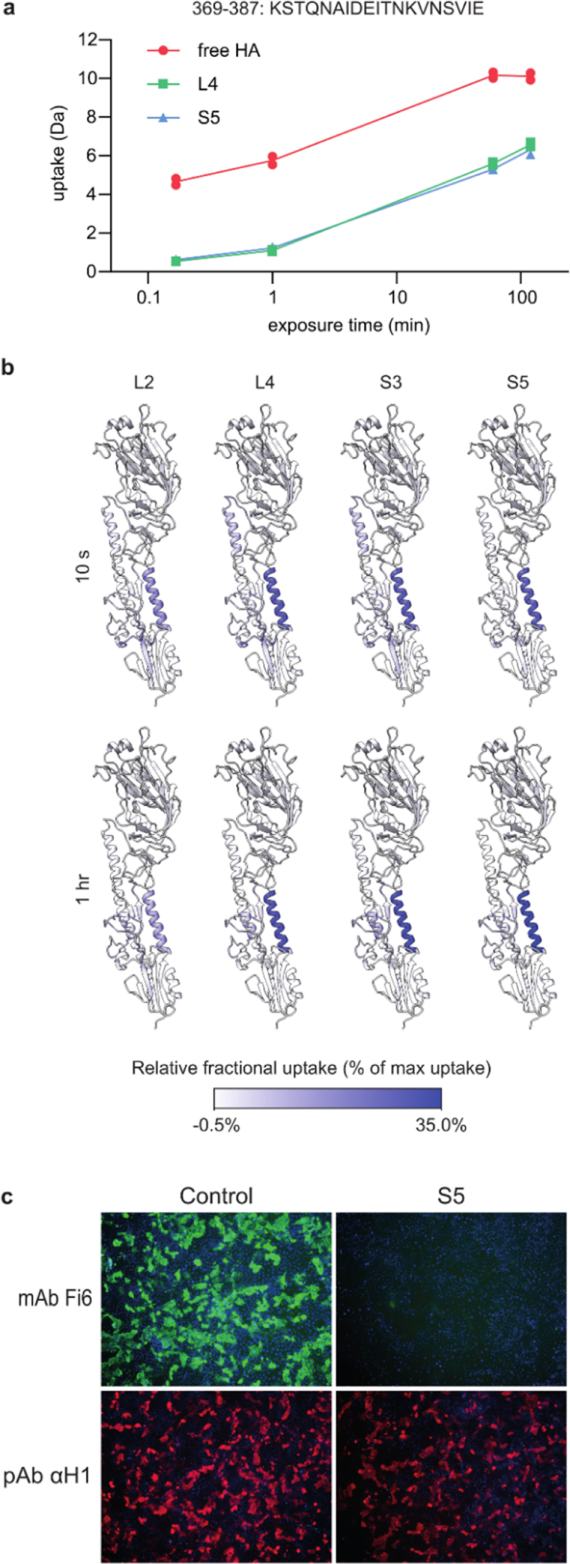
Neutralizing peptides bind helix A in the HA stem region. (a) Deuterium uptake of the H1 recombinant protein from the solvent was monitored using MS. Preincubation with the peptides resulted in a consistent mass shift in the HA peptide fragment 369–387 (H3 numbering), indicative of reduced deuterium uptake as a result of reduced access to the solvent. (b) Relative fractional uptake of deuterium presented as a heat map on the H1 protomer. (c) Cells transiently expressing membrane-bound H1 were stained with mAb Fi6 (green), which binds the HA stem, and pAb αH1 (red), which mainly binds the head domain. The binding of the S5 peptide competed with the binding of mAb Fi6.

To elucidate the binding site of our neutralizing peptides, we employed HDX-MS, which has previously been shown to be able to determine the binding epitope of hemagglutinin drug candidates.^27^ This technique uses MS to detect a change in mass associated with the exchange of hydrogen in the protein with deuterium that is provided in the solvent. Addition of a ligand, such as one of the peptides described here, changes the accessibility of the recombinant HA to the solvent, resulting in a distinctive “footprint” in the HDX-MS data (see Figure S11 for the HDX-MS coverage map of HA). Using this approach, all peptides analyzed (B1, B2, L2, L4, S2, S3, and S5) were found to bind the same region [aa 369–387; H3 numbering] in the HA stem (Figures 3 and S12). The identified binding site is a part of the helix A^28^ and belongs to a common epitope for broadly neutralizing antibodies (bnAbs) including Fi6,^29^ and therefore, we were able to confirm the HDX-MS findings with a competition experiment. Preincubation with peptide S5 before addition of Fi6 strongly decreased the staining of full length, membrane-bound H1 HA expressed on the surface of MDCK cells, while staining with the polyclonal anti-H1 antibody, which mainly targets the immunodominant head domain, was unaffected (Figure 3c). While protection from H–D exchange was most striking in helix A, several additional regions in the HA stem showed a minor degree of protection upon inhibitor binding (Figure S12). This includes the fusion loop, helix C, and the B-loop, which are all involved in the HA conformational change from the pre- to postfusion state.^28^ These findings suggest that inhibitor binding to the HA stem stabilizes the protein in its prefusion conformation.

The binding affinities of L2, L4, S3, and S5 for H1 were measured by fluorescence polarization (FP) using fluorescein-labeled peptides. Peptide L2 showed an affinity of 2.4 nM, while the remaining three peptides showed binding at or below the limit of detection (≤1 nM, based on 2 nM fluorescent peptide, Figures 1, S13, and S14 and Table S5) and thus are consistent with the low- to mid-nM IC_50_ values found in the infection assays. We further characterized binding kinetics by surface plasmon resonance (SPR) using biotinylated versions of peptides L4, S3, and S5 (Tables S7, S8 and Figures S15–S35). Because this assay used peptide immobilized on tetrameric streptavidin covalently coupled to the flow chip and hemagglutinin itself is trimeric, the SPR traces did not fit well with a simple 1:1 model but could rather be the best fit using a bivalent model. This makes obtaining an absolute *K*_d_ value for the interaction more complicated, and therefore, we interpret these results only qualitatively. Nevertheless, a clear indication could be observed for the overall affinity of the interaction and the differences between different HA constructs. All three peptides bind both the full H1 ectodomain and the H1 stem with apparent high affinity consistent with the low- to sub-nM *K*_d_ observed by FP, characterized by slow dissociation rates nearing the limit of detection. In agreement with the inhibition assays, peptides S3 and S5 bind to the H5 stem, albeit with lower affinity than the H1 stem. Surprisingly, the affinity of peptide S3 for the H5 stem was comparable with that of S5, despite the marked difference in infection inhibition. Future structural studies may clarify the origin of these differences in binding and activity for this peptide. The binding of L4 to the H5 stem was not detectable. The SPR setup also conveniently allowed us to profile the breadth of binding to other HA subtypes. As expected from the lack of activity against the H3 HA-containing virus (Figure 2d), none of the peptides showed any interaction with the full H3 ectodomain. Also, no interaction was observed for H9 HA. Binding in SPR thus shows a good correlation with infection inhibition, although it does not perfectly predict potency despite these peptides all binding to the same region. This may reflect subtle differences in binding interactions, but because of the quality of some traces in our data set, we note that we are careful not to overinterpret these differences.

**Figure 4:**
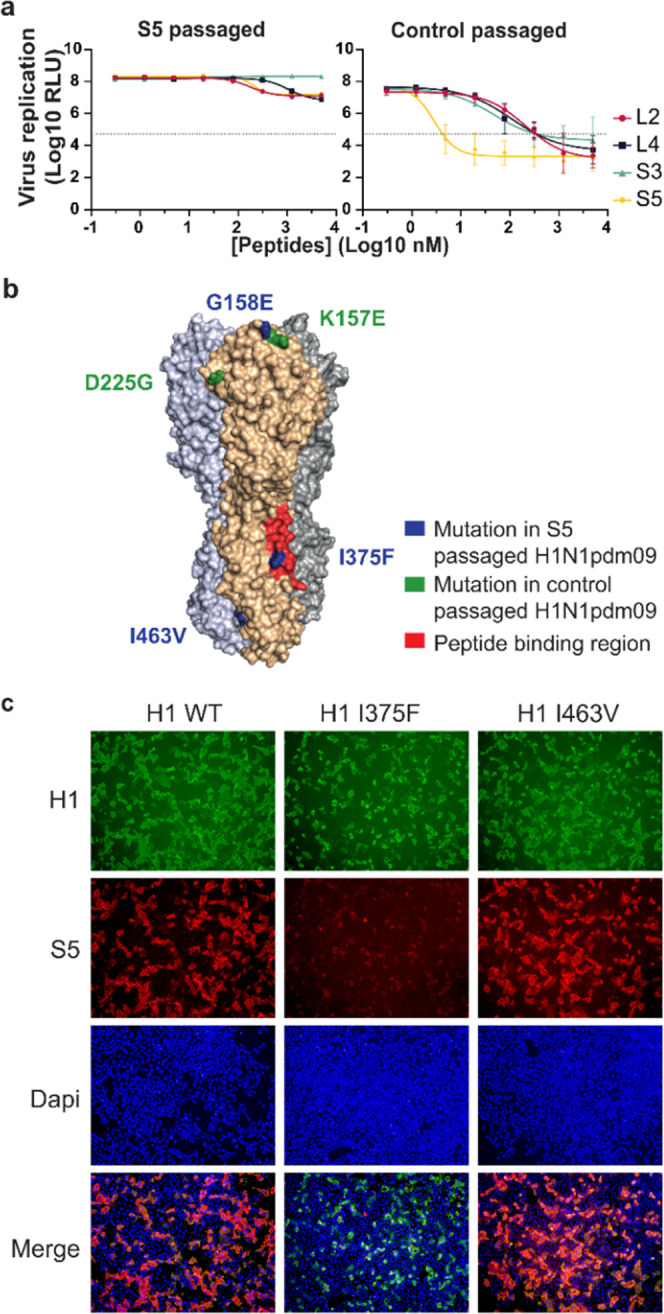
Substitution I375F identified in the HA of resistant H1N1pdm09 impedes peptide binding to H1. H1N1pdm09 was passaged five times on MDCKII cells in the presence or absence of the S5 peptide. (a) Virus passaged in the presence of S5 is no longer sensitive to inhibition by the peptides in a luciferase assay for viral replication. Data are shown as mean ± SD (*n* = 3). (b) Substitutions identified in the HA of the S5-passaged H1N1 virus (blue) and the control-passaged virus (green, H3 numbering). One substitution in the resistant virus, I375F, is located in the peptide-binding site as determined by HDX (red). (c) Immunofluorescence staining of cells expressing wild-type H1, or H1 with single substitutions in the stem region found in the resistant virus (I375F or I463V). HA is stained with pAb αH1 (green) and the biotinylated S5 peptide (red). Nuclei are labeled with DAPI (blue).

To confirm the macrocyclic peptide-binding site and to obtain more insights into the mechanism of inhibition, we next selected an escape variant of H1N1pdm09. The virus was passaged in low concentrations of the S5 peptide for several rounds until a resistant phenotype emerged. A luciferase-based virus neutralization assay confirmed that the S5-passaged variant gained resistance against neutralization with all four peptides (Figure 4a). The HA of this escape variant contained three substitutions in the head and stem domains. The substitution in the head domain (G158E; H3 numbering) is located directly adjacent to the position where a substitution was also identified in a virus passaged in the absence of the inhibitory peptide (K157E). Both residues are located close to the receptor-binding site and are likely selected for altered binding affinity (Figure 4b).^30^ The two substitutions identified in the stem domain (I375F and I463V) warranted further exploration for a potential role in resistance.

To assess the role of the substitutions in peptide binding, recombinant H1 stem proteins containing either one of the substitutions I375F or I463V were expressed for binding analysis with SPR. The I375F substitution resulted in a complete loss of binding to all peptides (L4, S3, and S5) whereas the I463V substitution did not alter peptide binding (Table S7). This result was further confirmed by analyzing the binding of biotinylated S5 to cells expressing full-length H1 HA at the cell surface. The binding of S5 was affected by the I375F substitution but not by the I463V substitution (Figures 4c and S36).

**Figure 5:**
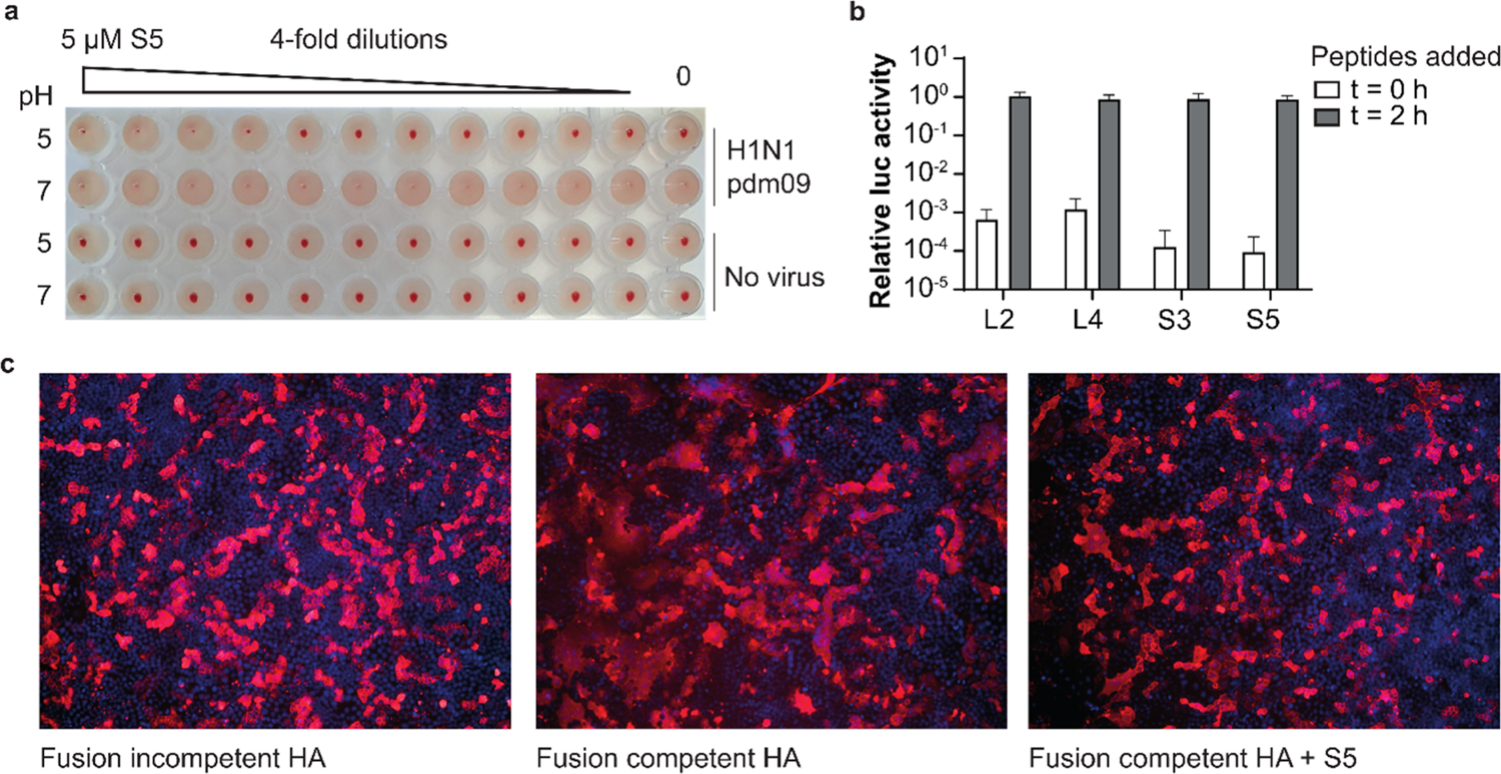
S5 inhibits HA fusion activity during entry by stabilizing the prefusion conformation. (a) S5 prevents pH-induced conformational changes of HA. H1N1pdm09 was incubated with serial dilutions of the S5 peptide, followed by incubation with buffers at pH 5 or 7, and finally, red blood cells (RBCs) to allow hemagglutination. Agglutinated RBCs distribute in the entire well, while nonagglutinated RBCs sediment to the bottom forming a red dot. Virus pretreatment at pH 5 caused the loss of hemagglutination activity as a result of conformational changes, which was prevented by S5. (b) Peptides inhibit infection only when present during entry. The peptides were either added at the same time as infection or 2 h later at the same moment that the inoculum was removed and the entry process halted with the addition of bafilomycin A1, which inhibits acidification of endosomes.^31^ Viral replication was measured as luciferase activity and plotted relative to a control condition without peptides. Data are shown as mean ± SD (*n* = 3). (c) S5 inhibits fusion between H1 expressing cells. Cells were exposed to pH 5 buffer to induce HA fusion activity and subsequent syncytia formation. HA was made fusion competent by trypsin treatment. HA is stained with pAb αH1 (red), and nuclei are labeled with DAPI (blue). Syncytia are visible as large red uninterrupted membrane structures

Helix A in the HA stem is involved in membrane fusion. The bnAbs that bind the same epitope as our peptides are known to neutralize IAV by stabilizing HA in the prefusion state and thus prevent the conformational changes required for fusion. The stabilization of recombinant HA by our peptides in the above thermal shift experiment (Figures S7 and S8) is consistent with this presumed mechanism. While the increased thermal stability indicates that the peptides may affect the conformational changes in HA involved in virus–cell fusion,^32^ the natural trigger for these changes is low pH in endosomes. A more physiologically relevant assay involving acidic pH was set up based on the ability of HA to bind sialic acid receptors on red blood cells, known as hemagglutination. Incubating H1N1pdm09 in a pH 5 buffer resulted in the loss of hemagglutination (Figure 5a), as a result of pH-induced conformational changes of HA.^33^ This process was prevented by preincubation of the virus with S5, indicating a stabilizing effect of the peptide on HA. The results of this assay also demonstrate that the peptide itself does not interfere with hemagglutination, thereby ruling out the possibility of an inhibitory mechanism based on interference with receptor binding, which is common for neutralizing antibodies that bind the HA head domain. In agreement with the peptides preventing pH-induced conformational changes of HA, they were found to inhibit virus infection when present during the entry stage, but not when added thereafter (Figure 5b). To confirm that the stabilizing effect of S5 leads to inhibition of fusion activity, a fusion inhibition assay was performed. To this end, H1 HA was expressed on the surface of MDCK cells, and the acidic conditions of the endosome were mimicked by incubation with a pH 5 buffer. These conditions trigger fusion driven by HA and result in cell–cell fusion, visible in the immunofluorescence assay as large syncytia consisting of uninterrupted membrane structures containing multiple nuclei. Syncytia formation was greatly reduced in the presence of S5 (Figure 5c), consistent with the proposed mechanism that the peptides inhibit conformational changes in HA required for virus–cell fusion.

## Conclusions

In this work, the RaPID system was used with a combination of an unbiased library and several different protein constructs as “bait” for the discovery of broadly active peptide inhibitors of IAV, targeting HA. These peptides bind with antibody-like affinities to a conserved region in the HA stem, resulting in broad activity against different group 1 HA subtypes. While the selection was carried out initially against H1 HA, the most potent peptide S5 also inhibits an H5 HA-containing virus equally well. Of note, H5Nx viruses are considered potential pandemic threats. Binding or inhibition of more distantly related H3- and H9-subtype viruses was however not observed, likely as a result of the multiple differences in the amino acid sequence of the peptide-binding site (Figure S37). Antibodies and other peptides targeting a similar region of HA are often also limited in their antiviral activity against either group 1 or 2 HA subtypes.^6,9,34,35^ While peptides blocking receptor binding may also be of interest, this preference for a stem binding site is fortuitous as it is less susceptible to antigenic drift.^36^ The macrocyclic peptide-binding site observed here is also distinct from that exploited by the drug arbidol,^37^ which binds to the interface between adjacent protomers of the HA trimer composed of helix A and C. The neutralizing effects of the peptides described in this study are based on stabilization of the prefusion state of HA. By binding to prefusion HA, they prevent the low pH-induced conformational changes needed for virus–cell fusion, an essential step in viral entry. Remarkably, all peptides analyzed in HDX-MS were found to bind the same HA stem region, regardless of the protein target they were enriched with (HA stem [S2, S3, and S5] vs full HA ectodomain [B1, B2, L2, L4]). This suggests that the binding of the macrocyclic RaPID peptides is biased toward the HA stem. These findings are notable since they contrast the immunodominance observed for HA after infection or vaccination. Antibodies have a clear preference for binding the head domain.^38^ This preference still stands when immunizations are done with recombinant soluble HA, indicating that the accessibility of the epitope in the context of virus particles is not the limiting factor for antibody development and binding.^38^

In another recent study published during preparation of this manuscript, the RaPID display technology was used to screen for binders of H5 HA (rather than H1 HA as used here).^20^ The resulting cyclic peptides were inferred to also target the stem of HA from resistance mutants, although the precise binding site was not defined and the mode of action appeared to also involve inhibition of viral adhesion, in contrast to our hemagglutination assay data. Those authors were able to show a protective effect for their best hit in mouse and primate models. This and another recent report^39^ are encouraging for the efficacy of a simple nasal administration route for the hits in the present report. Notably, our work appears to have yielded a larger number of active hits and achieved equal or better potency without the use of noncanonical amino acids beyond that used in cyclization, while still giving peptides active in the presence of serum. This may suggest that HAs differ in their suitability to serve as target for finding active antiviral peptides. Furthermore, the clearly defined binding site we observe for all seven peptides tested here allows us to conclude that this stem region has an unexpected dominance in such selections, in contrast to antibodies.

Neutralizing antibodies are often used to identify the “Achilles heels” of viruses: vulnerable sites that could be interesting targets for vaccines or antivirals. The macrocyclic peptides reported here seem to predominantly bind a dominant site within the stem rather than the globally immunodominant head region. These apparent differences in epitope recognition may allow for identification of novel targets of vulnerability, although for HA, this is relatively well mapped already. The immune-subdominant vulnerable site that our peptides bind to is a well-known target for bnAbs and experimental vaccines. However, for other viruses, a RaPID screen could provide valuable information, which may guide the design of vaccines or antivirals.

RaPID technology allowed us to select a large number of peptides that bind to HA, with 18 of 20 candidate hits showing target binding by a protein thermal shift and from those 9 of 9 tested inhibited infections. While it was hoped that peptides targeting different conserved sites of HA would have been identified, as combination therapy of such peptides would be more difficult for a virus to overcome, this nonetheless provides an abundant pool of candidates for future work. The I375F mutation identified in the H1N1pdm09 escape variant that resulted from passaging with the S5 peptide has previously been reported as a key mutation, providing resistance against bnAbs.^40,41^ Similar to the effect on antibodies, this mutation also allowed the escape of all of the peptide inhibitors studied here. Combination therapy of the current peptides with existing neuraminidase inhibitors on the market may be one way to prevent the development of such resistance.

## Methods

### Expression and Purification Of Recombinant Hemagglutinin Proteins

Recombinant soluble trimeric HA ectodomain proteins were produced using pFRT expression plasmids containing human codon-optimized cDNAs encoding the head and stem domains of A/California/04/2009 (H1N1), A/Fujian/411/2002 (H3N2), and A/turkey/England/13437/2013 (H9N2) fused to a GCN4 trimerization domain^42^ and Strep-tag (One-STrEP; IBA GmbH) as described previously.^43^ When indicated, the H1 ectodomain fusion protein was extended with a monomeric Fc domain^24^ between the GCN4 domain and the Strep-tag. An H1 stem construct was designed as described by Impagliazzo et al.^23^ and expressed fused to a Strep-tag. An H5 stem construct of A/Vietnam/1194/04 (H5N1) with the Strep-tag was designed similar to the H1 stem construct. Both stem-only proteins displayed a high affinity for stem-specific antibodies. A full-length H1 (H1 FL) expression construct was generated by replacing GCN4 and the Strep-tag with the native transmembrane domain and the cytoplasmic tail. An overview of the protein constructs is listed in Table S1. Plasmids were transfected into HEK293T or HEK293S GnTI–/– cells^44^ using polyethyleneimine (PEI) and 6 days later recombinant soluble proteins were purified from the supernatant using protein A- (GE Healthcare) or Strep-Tactin Sepharose resin (IBA), similarly as described previously.^43^

### Peptide Selection

Cyanomethyl esters of chloroacetylated d- and l-tyrosine (separate reactions) were charged on tRNA_CAU_^fMet^ as previously described.^14^ Based on a previously reported method,^25^ selections were carried out against either Fc- or Strep-tag-immobilized HA using Protein G or Strep-Tactin X magnetic beads, respectively, for both d- and l-tyrosine-initiated libraries in parallel. An overview of the protein construct and magnetic beads used in each round of the selection is listed in Table S2. The DNA library, which encodes for 15 randomized NNK codons followed by a section encoding a CGSGSGS linker, was assembled by PCR, followed by transcription to RNA using T7 RNA polymerase (NEB) at 1 mL overnight at 37 °C with 25 pmol of DNA input and then purification by denaturing agarose gel electrophoresis. Puromycin ligation by T4 RNA ligase (NEB), peptide translation using PURExpress (NEB), and reverse transcription by ProtoScript II Reverse Transcriptase (NEB) were all performed as reported previously.^25^ cDNA/mRNA-tagged peptide libraries were diluted with stock solutions of PBS-T and BSA to achieve final concentrations of 1× and 0.1%, respectively, with a total volume of 25 μL in the first selection round and 14 μL in subsequent rounds; 1 μL of the reverse transcription reaction was diluted in 500 μL of milliQ to serve as an input sample for qPCR analysis. The translation mixes were then sequentially incubated three times for 20 min at 4 °C with 2.5 μL of magnetic beads without the target protein to preclear any peptides binding to Strep-Tactin XT or Protein G beads (omitted for the first round). The supernatants were then incubated with beads carrying hemagglutinin followed by stringent washing with 3 × 20 μL of PBS-T, using conditions listed in Table S2. Bound peptides were then eluted by incubation with 50 μL of MilliQ water for 5 min at 95 °C. The same wash and elution steps were performed on the third preclear bead, which is used as a negative sample in the qPCR analysis. The samples of the input, positive, and negative (1 μL each) were analyzed by qPCR alongside a standard curve, which was obtained by reverse transcription of the input library. Recoveries were calculated for the positive and negative selections by dividing the respective amount by the amount of the input sample after compensating for dilution factors. The remaining solutions of the positive selections were amplified by PCR to act as input for the next round of selection, starting again with transcription to mRNA (without gel purification). This process was repeated until positive samples indicated enrichment above the background after which the DNA output was used for sequencing on the Illumina MiSeq platform using a 2 × 150 bp V2 reagent kit at the Utrecht UMC sequencing facility (USEQ). Raw sequencing data are available from the DataverseNL repository at https://doi.org/10.34894/996EYR

### Peptide Synthesis

All peptides shown in Tables S3 and S4 were synthesized using microwave-assisted Fmoc solid-phase peptide synthesis on Rapp Polymere (Germany) TentaGel S RAM resin on a Liberty Blue (CEM) peptide synthesizer at a 25 μmol scale. Each coupling step of 4 min was performed with 5 equiv of amino acid, 5 equiv of Oxyma Pure, and 10 equiv of *N,N*′-diisopropylcarbodiimide (DIC) in DMF at 90 °C. Fmoc deprotection was performed using 20% piperidine in DMF for 1 min at 90 °C. Automated synthesis was followed by *N-*terminal chloroacetylation using 5 equiv of chloroacetic acid instead of amino acid in the described coupling step or *N*-acetylation by treatment with 20% acetic anhydride in DMF for 2 min at 65 °C. Peptide cleavage, global deprotection, cyclization, and subsequent purification were performed as reported before.^45^ Purified peptides were analyzed by UV-HPLC using a 40 min gradient from 100% buffer A (95% water, 5% acetonitrile + 0.1% TFA) to 70% buffer B (5% water, 95% acetonitrile + 0.1% TFA) with a Dr Maisch (Germany) Silicycle C_18_ column (150 × 4.6 mm, 5 μm). These UV-HPLC traces can be found in Figures S36–S39. Further details of specific syntheses are provided in the Supporting Information.

### Thermal Shift Assay (TSA)

The partially purified peptides were diluted to 200 μM in DMSO, estimated by UV absorbance at 280 nm with calculated extinction coefficients (Expasy ProtParam, Swiss Bioinformatics Resource Portal). The final solutions of the TSA reaction mixture contained 5 μM of the peptide crude, 0.05 mg/mL protein, and 2 × SYPRO orange dye (Thermo Fisher Scientific) in 100 mM HEPES and 150 mM NaCl pH 7.5. Control conditions contained an equal volume of DMSO compared to peptide solution, leading to 5% DMSO in the final reaction solution. TSA was measured on a PCR machine (Bio-Rad CFX96) using the “melting assay” protocol with increase steps of 0.2 °C for 10 s from 30 to 80 °C. Fluorescent intensity was measured using FRET filter settings. All measurements were performed in duplicate and averaged to obtain the final graphs (Figure S8). Tm was then determined using the highest value of the d(RFU)/dT peak.

### Fluorescence Polarization (FP) Binding Assays

Fluorescence polarization assays were performed in black, low-volume, nonbinding 384 well microplates “784900” (Greiner Bio-One, the Netherlands) using a BMG Labtech (Germany) PHERAstar FS microplate reader. Assay buffer consisted of 10 mM Tris-HCl pH 7.5, 150 mM NaCl, and 0.01% Tween-20. H1 ectodomain (see Table S1) was titrated in triplicate to 5 or 2 nM of FAM-labeled peptides by performing a 1:1 serial dilution. Measurements were performed after 30 min incubation at room temperature (Excitation: 485 nm, EmissionA: 520 nm, EmissionB: 520 nm). Data were fit using GraphPad Prism 8.4.3 software to the following equation
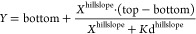


### Virus Neutralization Assays

Virus neutralization was assessed by immunofluorescence staining and a luciferase reporter assay. For staining, MDCKI cells were seeded in 96-well plates (1.5 × 10^4^ cells/well) and incubated for 24 h. Peptide dilutions were prepared in triplicates in OptiMEM medium (Gibco) in separate plates, mixed with an equal volume of the diluted virus, and incubated for 10 min at 20–22 °C before the mixture was added to the cells. At 20 h post infection, the cells were fixed with ice-cold methanol for 15 min and blocked with 2% BSA (BioIVT) in PBS. Immunofluorescence staining was performed with a mouse anti-influenza nucleoprotein monoclonal antibody (HB65) harvested from hybridoma H16-L10-4R5 (ATCC) and polyclonal Goat anti-Mouse IgG Alexa Fluor 488 (Invitrogen). Nuclei were labeled with DAPI (Invitrogen). Cells were imaged using the EVOS imaging system with a 10× objective. The results were quantified by counting the infected cells in Fiji ImageJ.

For virus neutralization using the luciferase reporter assay, HeLa R19 cells were seeded in 96-well plates (10^4^ cells/well). Cells were transfected the next day with the pHH-Gluc luciferase reporter plasmid^46^ using the FuGENE transfection reagent (Promega) according to the manufacturer’s instructions. The pHH-Gluc plasmid contains the Gaussian luciferase gene, flanked by 3′ and 5′ untranslated regions of the IAV NP genome segment, under the control of the RNA polymerase I promoter in the negative sense orientation. Generation of luciferase-encoding mRNAs and thus of the luciferase protein is only observed after infection of transected cells with IAV. After a further 24 h incubation, a virus with peptides was added as described above. At 16 h post infection, the Renilla luciferase assay system (Promega) was used according to the manufacturer’s instructions to assay samples of the cell supernatant for luciferase activity. Luminescence was measured in relative light units (RLUs) using Berthold or Promega luminometers. The half-maximum inhibitor concentration (IC_50_) was calculated based on two independent experiments, each containing three biological replicates using curve fitting in GraphPad Prism 9.1.0.

Virus neutralization with the S5 peptide in the presence of serum proteins was performed by adding indicated percentages of fetal calf serum (Sigma-Aldrich) to the OptiMEM medium in which the peptide was diluted. The assay was then continued as described above.

### Hydrogen–Deuterium Exchange Mass Spectrometry

For the initial peptide identifications, an estimated 90 pmol of the H1 ectodomain of A/California/04/2009 (H1N1) expressed from HEK293S GnTI–/– cells was diluted in a total volume of 40 μL of PBS, pH 7.4 and mixed with 20 μL quench solution consisting of 6 M Urea and 300 mM TCEP, pH 2.5.

Directly after mixing the sample with the quench solution, the complete volume of the sample was manually injected into a 50 μL sample loop of the nanoACQUITY UPLC System with HDX Technology (Waters Corp.). The protein was loaded onto the immobilized pepsin column (Waters) for digestion (20 °C) followed by in-line trapping (0.5 °C) of the formed peptic peptides on a Waters Van Guard BEH C18 trap column (300 Å, 1.7 μm, 2.1 × 5 mm) for 3 min at 125 μL·min^–1^. Peptic peptides were separated with a Waters Acquity UPLC BEH C18 analytical column (1.7 μm, 1.0 × 100 mm) with a 15-min gradient from 8 to 95% B, where A is 0.1% formic acid and B is 0.1% formic acid in acetonitrile.

Separated peptides were analyzed with a XEVO G2 mass spectrometer (Waters Corp.) with MSE data acquisition with 6 V fixed collision energy during the low energy scan and 10–35 V ramped collision energy applied during the high energy scan. Cone/extraction cone voltages were set to 40/4 V, respectively. The data were acquired in the resolution mode within the 50–2000 *m*/*z* range. The peptide identification measurements were performed in triplicate. Peptides were identified with Waters PLGS 3.0.1 software (digestion was set to nonspecific, with methionine oxidation and N-glycans as variable modifications) and processed with Waters DynamX 3.0 (minimum intensity was set to 1000, minimum of four amino acids were considered as a peptide, and the peptide was present in two out of three PLGS files). After this initial filtering, the peptides were visually inspected and selected for spectrum quality, omitting peptides with wrongly assigned charge states and interfering signals.

HDX reactions were carried out at 37 °C in 95% D_2_O with a final concentration of 50 mM Tris, 150 mM NaCl, pD 8.0. HA was preincubated for approximately 10 min at 10 μM with either a 4-fold molar excess of the cyclic peptide inhibitors diluted from their DMSO stock solution or the equivalent amount of DMSO for free HA. A volume of 5 μL of the preincubated HA was then mixed with 35 μL of the D_2_O solution to carry out the exchange reaction for the indicated times.

An initial time course with exposure times of 10 s, 1 min, 60 min, and 120 min was performed in duplicate for L4 and S5 inhibitors. Subsequently, HDX-MS was performed on a broader panel of inhibitors including B1, B2, L2, L4, S2, S3, and S5 for 10 s and 60 min in duplicate. The quenching, digestion, and LC-MS measurement of the HDX reactions were carried out as described above, with the omission of the MSE scans from the MS method. The deuterium uptake of unbound HA was compared to the deuterium uptake of HA inhibitor complexes in DynamX 3.0.

### Selection for Escape Variants

To identify putative peptide-resistant mutations, H1N1pdm09 was passaged in the presence of S5. MDCKII cells were seeded at 10^4^ cells/well in 96-well plates. The next day, dilutions of the S5 peptide were prepared in a separate plate before adding the virus to a final amount of 50 units of 50% tissue culture infectious dose (TCID50)/well. All dilutions were made in OptiMEM with 1 μg/mL trypsin to allow multicycle infection. Wells containing the same amount of the virus without peptide inhibitors were included for reference. The cells were washed with PBS, while the virus was incubated with peptides for 15 min at RT, after which the cells were incubated with the virus–peptide solutions in six replicates. At 72 h post infection, CPE was scored, and supernatants of wells with the highest S5 concentration where CPE was observed were collected and pooled. The supernatant of the infected wells without peptides was collected similarly. Passaged viruses were stored and aliquoted at −80 °C until further use. After each passage, the virus titer was determined by TCID50 analysis, and the next passage was performed as described above. After five passages, CPE was observed at higher peptide concentrations, indicative of emerging escape variants. The cell culture supernatant after the fifth passage was used for sequence analysis and was passaged one additional time in the absence of an inhibitor to generate the virus stock to be used in the neutralization assays.

### Sequence Analysis of Escape Variants

To identify mutations in the passaged viruses, viral RNA was extracted from the supernatant using a NucleoSpin RNA virus kit (Macherey-Nagel) according to the manufacturer’s instructions. cDNA was synthesized from the purified RNA using random hexamer primers and Superscript III Reverse Transcriptase (Invitrogen). The cDNA encoding HA was amplified using Q5 High-Fidelity DNA polymerase (NEB) and two sets of primers combined to cover the whole HA encoding sequence. PCR products were separated on agarose gel, purified using Nucleospin Gel and a PCR clean-up kit (Macherey-Nagel) according to the manufacturer’s instructions, and sequenced by Sanger sequencing (Macrogen).

### Peptide Binding to Membrane-Bound H1

The binding of S5 to H1 in its native membrane-bound form was evaluated using cells expressing full-length H1 on the plasma membrane. MDCKI cells were seeded in 96-well plates at 1.5 × 10^4^ cells/well. The cells were transfected the next day with pFRT expression plasmids encoding the full-length H1 of A/California/04/2009 (H1N1, wild-type, I375F, or I463V variant) using Lipofectamine 2000 (Invitrogen) according to the manufacturer’s instructions. After overnight incubation, the cells were incubated with dilutions of the biotinylated S5 peptide for 2 h. The cells were washed, fixed with 3% paraformaldehyde, and blocked with 2% BSA (BioIVT) in PBS with 50 mM NH_4_Cl. For immunofluorescence analysis, staining was performed using polyclonal sheep anti-H1 (against purified HA of A/California/7/09 (H1N1)), provided by the National Institute for Biological Standards and Control (NIBSC, London, U.K.), followed by Donkey anti-Goat IgG DyLight 488 (Life Technologies). The binding of S5 was detected by incubation with streptavidin conjugated with Alexa Fluor 568 (Invitrogen). For the competition experiment with an antibody recognizing the HA stem epitope, the cells were stained with human mAb Fi6^29^ and sheep anti-H1 antiserum (NIBSC, London, U.K.), followed by Donkey anti-Human IgG Alexa Fluor 488 (Jackson ImmunoResearch) and Donkey anti-Goat IgG Alexa Fluor 594 (Life Technologies). Nuclei were stained with DAPI (Invitrogen). The cells were imaged with an EVOS FL Cell Imaging System at 10× magnification.

For analysis in a cell-based ELISA assay, the cells were incubated with HRP-conjugated streptavidin and reference wells were incubated with sheep anti-H1 antiserum (NIBSC, London, U.K.) followed by HRP-conjugated protein A/G (Pierce). The cells were subsequently incubated with TMB (Super Slow One Component HRP Microwell Substrate, BioFx), and after 3–5 min, the reaction was stopped with 25% sulfuric acid (Merck). Absorbance was measured at 450 nm in an ELISA plate reader, and data were corrected for H1 expression levels (minimally differing based on polyclonal antiserum staining) and background binding of peptides to cells not transfected with H1.

### pH-Dependent Hemagglutination Assay

The ability of the S5 peptide to stabilize the structure of hemagglutinin under low pH conditions was evaluated in a hemagglutination assay. Dilutions of the S5 peptide were prepared in a V-bottom 96-well plate in the OptiMEM medium. H1N1pdm09 was added at four hemagglutination units per well and incubated for 30 min at RT. PBS (pH 7) or citrate buffer with 0.15 M NaCl (pH 5) was added and incubation was continued for 30 min at 37 °C. A 0.5% red blood cell (RBC) suspension (Sanquin) in PBS was prepared in a new V-bottom 96-well plate, while the plate with the virus was allowed to cool down to RT. The virus was transferred to RBC and incubated for 2 h at 4 °C.

### Fusion Assay

MDCKI cells were seeded in 96-well plates at 10^4^ cells/well. The next day, the cells were transfected with pFRT expression plasmids encoding the full-length H1 of A/California/04/2009 (H1N1) using Lipofectamine 2000 (Invitrogen) according to the manufacturer’s instructions. After the removal of the transfection mixture the next day, the cells were incubated for another day before starting the fusion assay, to allow optimal attachment and monolayer formation. First, the cells were washed with PBS and incubated with 0.5 μg/mL trypsin from bovine pancreas (Sigma-Aldrich) in the OptiMEM medium for 30 min at 37 °C to allow for proteolytic cleavage to make H1 fusion competent. Next, the cells were incubated with 5 μM S5 peptide for 1 h at 37 °C. Fusion was then initiated by 1 min incubation with a low pH buffer containing 50 mM sodium acetate and 0.15 mM NaCl buffer at pH 5.0. After washing with OptiMEM, the cells were incubated with 5 μM S5 in OptiMEM for 3 h at 37 °C before fixation with methanol as described above. The cells were stained with sheep anti-H1 antiserum (NIBSC, London, U.K.) and Donkey anti-Goat IgG Alexa Fluor 594 (Life Technologies) and nuclei were labeled with DAPI (Invitrogen). Imaging was performed using the EVOS FL Cell Imaging System at 10× magnification.

## Supporting Information Available

Supporting materials and methods; sequence analysis; synthetic details; thermal shift curves; infection assays; deuterium uptake data; fluorescence polarization data; and SPR data (PDF)
Extracted raw data (ZIP)
