# Supplementary material for: Inhibition of H1 and H5 Influenza A Virus Entry by Diverse Macrocyclic Peptides Targeting the Hemagglutinin Stem Region
Source: ACS Chem Biol. 2022 Aug 4;17(9):2425–36. doi: 10.1021/acschembio.2c00040 (PMC9486808; doi:10.1021/acschembio.2c00040)
Supplement: Supplementary file 1 — cb2c00040_si_001.pdf [file cb2c00040_si_001.pdf]

Supporting information for:

Inhibition of H1 and H5 influenza A virus entry by diverse macrocyclic peptides targeting the hemagglutinin stem region

Mirte N. Pascha,[1+] Vito Thijssen, [2+] Julia E. Egido[1,2], Mirte W. Linthorst[3], Jipke H. van Lanen[1], David A. A. van Dongen[2], Antonius J. P. Hopstaken,[4] Frank J. M. van Kuppeveld[1], Joost Snijder[3], Cornelis A. M. de Haan[1*], Seino A. K. Jongkees[2,4*]

1 Section Virology, Division Infectious Diseases and Immunology, Department of Biomolecular Health Sciences, Faculty of Veterinary Medicine, Utrecht University, Yalelaan 1, 3584 CL Utrecht, The Netherlands
2 Department of Chemical Biology & Drug Discovery, Utrecht Institute for Pharmaceutical Sciences, Utrecht University, Universiteitsweg 99, 3584 CG Utrecht, The Netherlands
3 Biomolecular Mass Spectrometry and Proteomics, Bijvoet Center for Biomolecular Research and Utrecht Institute for Pharmaceutical Sciences, Utrecht University, Padualaan 8, 3584 CH Utrecht, The Netherlands
4 Department of Chemistry and Pharmaceutical Sciences, Amsterdam Institute for Molecular and Life Sciences, VU Amsterdam, de Boelelaan 1108, 1081 HZ Amsterdam, The Netherlands

*Email: S.A.K.Jongkees@VU.nl
C.A.M.deHaan@UU.nl

1. Peptide sequence analysis

Sequences were extracted by Python script matching the T7 polymerase binding site in the forward direction and the puromycin ligation site in the reverse direction to find open reading frames, and then converting these to peptide sequences *in silico* with an adjusted genetic code (D- or L-tyrosine in the initiation position, depending on the library). Unique peptide sequences were tallied and then ranked by abundance, and sequence tallies converted to percentage using the total number of viable reads for each library. The top 100 most abundant unique sequences were aligned, and sequence families identified visually (based on substantial overlap across multiple unique sequences). The sequence family member of highest abundance was typically chosen for testing. For the D-tyrosine initiated library, sequences were further analysed by taking the change in sequence read fraction across the H1 and H5 stem selections as compared to round 3 of the main selection (multiplying relative enrichment factors to find sequences that increase substantially in both).

For the L-tyrosine initiated library sequences were named "L*n*", where *n* is sequencing abundance rank in round 5.

Sequences from the D-tyrosine initiated library were named based on apparent likely binding sites – sequences that showed an increase in sequence abundance from round 3 to the H1 and H5 stem rounds were labeled "S*n*" ('Stem'), where *n* is rank in list of compounded enrichment factors from round 3 to the stem binding rounds calculated according to the following equation:

$$\frac{F_{H5stem.R3}}{F_{H1.R3}} \times \frac{F_{H1stem.R3}}{F_{H1.R3}}$$

$F_{H1.R3}$ = fraction of total sequences in round 3 of the H1 selection

$F_{H5stem.R3}$ = fraction of total sequences in the H5 stem selection using round 3 as input

$F_{H1stem.R3}$ = fraction of total sequences in the H1 stem selection using round 3 as input

Sequences that showed a decrease in the same compounded enrichment factor across the same rounds were labeled "R*n*" ('Receptor binding domain'), and those that did not change substantially were labeled "B*n*" ('Broad'), where *n* is an arbitrary number in a manually curated list but broadly based on high sequence abundance having a lower number.

For an example calculation, the following data were observed for the peptide eventually labeled as S5:

Round 3 of initial selection returned 3 matching sequences out of 139168 total valid reads

Selection against H1 stem returned 144 matching sequences out of 112906 total valid reads

Selection against H5 stem returned 328 matching sequences out of 131765 total valid reads

$F_{H1.R3}$ = 3/193168 = 0.00155%

$F_{H5stem.R3}$ = 144/112906 = 0.127%

$F_{H1stem.R3}$ = 328/131765 = 0.249%

This gives a compounded enrichment factor of $\frac{0.127}{0.00155} \times \frac{0.249}{0.00155}$ = 13162 (relative value, no units)

2. Supplementary peptide synthesis

Synthesis of crude peptides for thermal shift analysis

Peptides were synthesized by standard automated Fmoc solid-phase synthesis on Rapp Polymere (Germany) tentagel S RAM resin using a Syro II (Biotage, Sweden) system at 5 µmol scale. Four equivalents of amino acid, four equivalents of 1-hydroxybenzotriazole (HOBt)/*N*-[(1*H*-benzotriazole-1-yl)(dimethylamino)methylene]-*N*-methylmethanaminium hexafluorophosphate *N*-oxide (HBTU), and eight equivalents of *N*,*N*-diisopropylethylamine (DIPEA) were used in each 40 min coupling step. This was followed by deprotection for 3 min using 40% piperidine in DMF, followed by 10 min 20 % piperidine in DMF. After coupling and deprotection steps for all the amino acids, the peptides were capped on the *N*-terminus with four equivalents of chloroacetic acid, four equivalents of HOBt/HBTU and eight equivalents of DIPEA for 30 minutes twice. Peptides were then cleaved, precipitated and cyclized as reported before.[1] To remove excess TFA and small impurities, peptides were subjected to a fast HPLC purification using a 5 minute gradient from 100% buffer A (95% water, 5% Acetonitrile + 0.1% TFA) to 100% buffer B (5% water, 95% Acetonitrile + 0.1% TFA) on preparative HPLC. Sequences of peptides synthesized for thermal shift analysis can be found in table S4. UV-HPLC traces of the peptides are shown in figures S40-S60.

Synthesis of biotinylated peptides

A linker with sequence Fmoc-GK$_{biotin}$G was manually synthesized on RAPP polymere (Germany) tentagel S RAM resin at 200 µmol scale. Coupling of glycine residues was achieved by coupling with 8 equivalents of amino acid, 8 equivalents of HBTU and 16 equivalents of DIPEA for 45 minutes in DMF twice at room temperature. Coupling of K(biotin) was performed by using 1.5 equivalents of N-α-Fmoc-N-ε-biotinyl-L-lysine, 1.5 equivalents of Oxyma Pure and 3 equivalents of DIC for 16 hours in DMF at room temperature. Deprotection of the Fmoc group preceding coupling steps was performed at room temperature by using 20% piperidine in DMF for 10 minutes twice. The on-resin synthesized Fmoc-GGK(biotin)G sequence was then elongated with the desired peptide sequence by using the synthesis steps as reported for all peptides.

Synthesis of FAM-FLAG labelled peptides

Peptides were synthesized by standard automated Fmoc solid-phase synthesis on Iris Biotech (Germany) tentagel XV RAM resin using a Gyros Protein Technologies (USA) PurePep Chorus peptide synthesizer at 25 µmol scale with C-terminal azidohomoalanine (prepared following the literature[2]). Fmoc deprotection was performed at room temperature for 5 min with 20% piperidine and 0.1 M Oxyma Pure in DMF. Coupling steps of 15 min were performed with 10 equivalents of amino acid/PEG-linker/chloracetic acid, 10 equivalents of Oxyma Pure and 20 equivalents of DIC in DMF at 55 °C. After each cycle uncoupled peptides were capped at room temperature for 5 min with 2 M acetic anhydride and 2 M pyridine in DMF. Peptide

cleavage, global deprotection, cyclization and purification were performed as described previously[1] with 2,2′-(ethylenedioxy)diethanethiol used instead of ethane-1,2-dithiol. Following purification by HPLC, 0.75 equivalents of DBCO-FAM (Lumiprobe, Germany, catalogue number 551F0) were conjugated onto the peptides at room temperature in 100 µL DMSO for 2.5 hours. Conjugated peptides were then separated from unconjugated peptides by a further round of preparative HPLC.

Sequences and MS data of all peptides are shown in tables S3 and S4 and UV-HPLC traces of all peptides can be found in figures S38-59.

3. Influenza viruses

H1N1 influenza viruses A/Netherlands/602/2009 (H1N1pdm09) and A/PR8/8/34/Mount Sinai (H1N1pr8) were characterized previously.[3,4] Generation of recombinant H5N1 and H3N1 viruses in the background of H1N1pr8 has been described previously.[5,6] H5N1 contains the HA and NA genes from A/duck/Hunan/795/2002 (H5N1)[5], while H3N1 contains the HA gene of A/Bilthoven/1761/76 (H3N2)[6]. The H3N1 virus was kindly provided by Ron Fouchier (Erasmus Medical Center, the Netherlands). Viruses were grown in MDCK-II cells (ATCC) as described previously[3] and stored aliquoted at −80 °C until use.

4. Surface plasmon resonance (SPR)

SPR experiments were performed using a Biacore T100 (GE Healthcare) instrument at 25 °C. First Streptavidin (Promega) was covalently immobilized on a CM5 S sensor chip (Cytiva, GE Healthcare) using an amine coupling kit (Cytiva, GE Healthcare) using manufacturers recommended conditions. In brief, the chip was preactivated with 70 µl of EDC (1-ethyl-3-(3-dimethylaminopropyl) carbodiimide) and NHS (N-hydroxysuccinimide) mixture, followed by injection of 70 µL Streptavidin solution (50 mM NaOAc pH 5.0, 100 ng/µL). Remaining active ester was blocked by injection of 70 µL ethanolamine (1 M, pH 8.5) followed by washing with HBS-T buffer (10 mM HEPES-KOH, pH 7.5, 140 mM NaCl, 0.005% Tween-20) to obtain a stable baseline. Peptides were then immobilized on the Streptavidin chip by injecting 100 nM peptide solution at 10 µl/min for 3 minutes, followed by washing with HBS-T as buffer until a stable baseline was acquired. Binding constants were obtained by injection of a series of protein solutions at a flow rate of 50 µL/min for 1 minute. A extended final dissociation step of 60 minutes was incorporated to produce a better off-rate estimation. Binding parameters were obtained using single cycle kinetics by fitting with bivalent analyte model. Regeneration was achieved using glycine-HCl (10 mM pH 2.0).

5. Cell viability assay

HeLa R19 and MDCKI cells were seeded in 96 well plates at 10^4 cells/well. The next day, peptide dilutions were prepared in triplicates in OptiMEM medium and transferred to the cells. After 72 hours of incubation with peptides, cells were incubated with Wst-1 cell proliferation reagent (Sigma-Aldrich) diluted 1:10 in OptiMEM for 4 hours. Absorbance was measured at 450 nm in ELISA plate reader.

6. Site-directed mutagenesis of H1

Substitutions identified in the HA of the H1N1pdm09 escape variant were introduced into H1 proteins for further characterization. Mutagenesis was performed using Q5 Site-Directed Mutagenesis kit (NEB) on expression plasmids encoding the H1 stem domain or the full-length

H1 of A/California/04/2009 (H1N1) using primers containing the desired substitutions.
H1CA09
I375F: CCAGAACGCCttCGACGAGATC (forward), GTGCTCTTCAGGTCGGCG (reverse)
I463V: CGCCAAGGAGgTCGGCAACGG (forward), TTGTTCTTCAGCTGGCTGCG (reverse)
H1stem
I375F: ACAGAACGCCtTTAACGGCAT (forward), GTGCTTTTCTGATCGGCG (reverse)
I463V: CGCCAAGGAGgtgGGGAACGGCT (forward), TTGTTCTTCAGCTGGCTCTTGACC (reverse)].
Mutagenesis was confirmed with Sanger sequencing (Macrogen).

Table S1. Overview of HA constructs

GCN4: leucine zipper trimerization domain; OS: One-STrEP affinity *tag; mFc: monomeric Fc domain.*

Protein	Strain	Domains	Tags	Variants	Genbank accession no.
**H1**	A/California/04/2009 (H1N1)	Ectodomain	GCN4<br>OS	-	MN371616.1
**H1**	A/California/04/2009 (H1N1)	Ectodomain	GCN4<br>mFc<br>OS	-	MN371616.1
**H1stem**	A/Kentucky/UR06-0258/2007 (H1N1)	Stem	OS	I375F<br>I463V	CY028163.1
**H1 FL**	A/California/04/2009 (H1N1)	Full HA	-	I375F<br>I463V	MN371616.1
**H5stem**	A/Vietnam/1194/04 (H5N1)	Stem	OS	-	EF541402.1
**H3**	A/Fujian/411/2002 (H3N2)	Ectodomain	GCN4<br>OS	-	KM821324.1
**H9**	A/Turkey/England/13437/2013 (H9N2)	Ectodomain	GCN4<br>OS	-	KU171016.1

Table S2. Summary of binding and wash step conditions of the peptide library

Selection round	Bead type	Amount of beads per library	Binding step conditions	Wash step conditions
1	Strep-Tactin XT	3 µl	1 hour, 4°C	No incubation
2	Protein G	1.5 µl	1 hour, 4°C	No incubation
3	Strep-Tactin XT	1.5 µl	1 hour, 4°C	No incubation
4	Protein G	1.5 µl	5 minutes, 37°C	Overnight 37°C
5	Strep-Tactin XT	1.5 µl	5 minutes, 37°C	Overnight 37°C
H1stem	Strep-Tactin XT	2 µl	45 minutes, RT	No incubation
H5stem	Strep-Tactin XT	2 µl	45 minutes, RT	No incubation

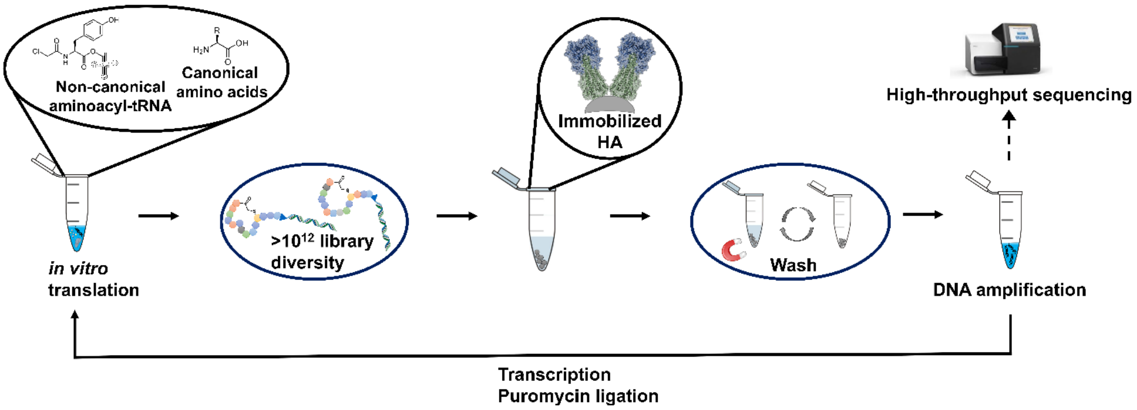

Figure S1 Schematic overview of the RaPID selection process used in this work. A DNA library containing 15 randomized (NNK) codons was translated using an *in vitro* translation system, initiating with a chloroacetylated tyrosine (added as aminoacyl-tRNA complex) that cyclizes with a cysteine sidechain, resulting in highly diverse cyclized peptide library (>10^{12}). After reverse transcription, the library was panned against immobilized HA on magnetic beads (Table S2). Stringent washing of the beads after incubation with the library aims to ensure the recovery of only tight binding peptides. Bound peptides are then eluted and the DNA is amplified by PCR and transcribed to RNA, followed by puromycin ligation to the mRNA to serve as input for a next round of selection. After successful enrichment of the peptide library, DNA is analyzed by high-throughput sequencing.

a

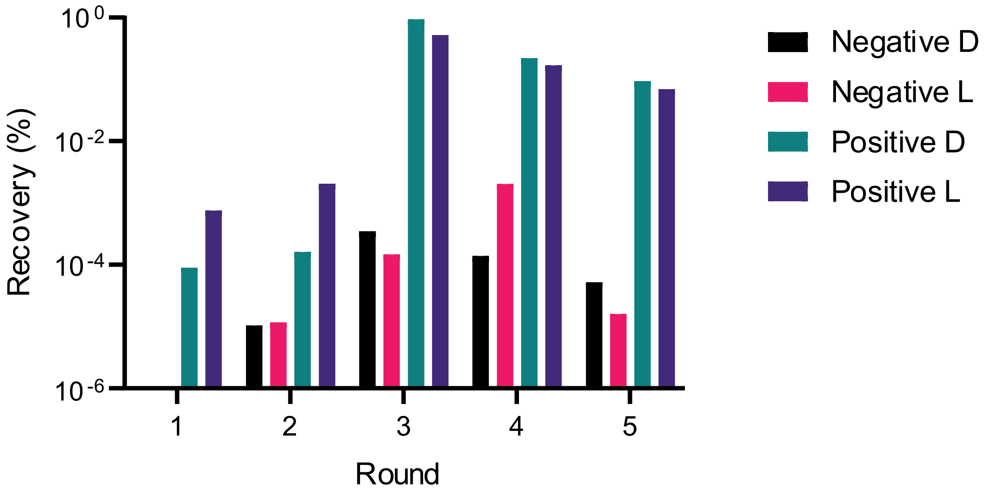

10^0
10^-2
10^-4
10^-6
Recovery (%)
1
2
3
4
5
Round
Negative D
Negative L
Positive D
Positive L

b

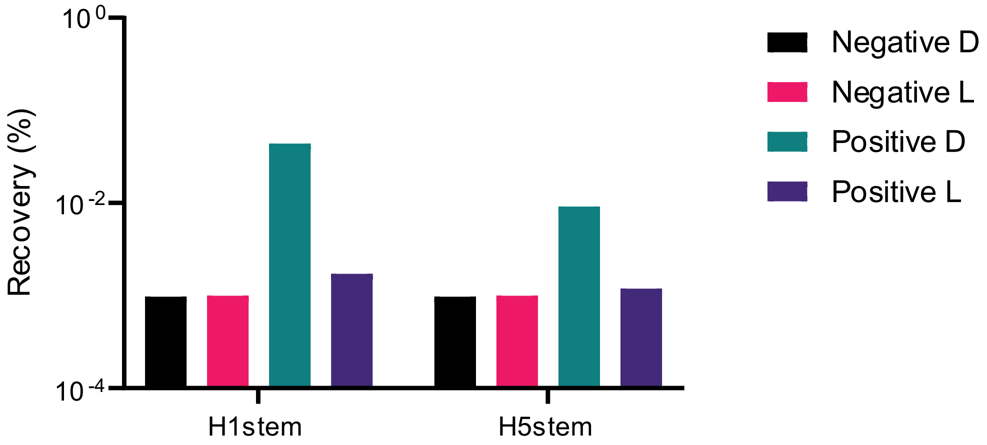

10^0
10^-2
10^-4
Recovery (%)
H1stem
H5stem
Negative D
Negative L
Positive D
Positive L

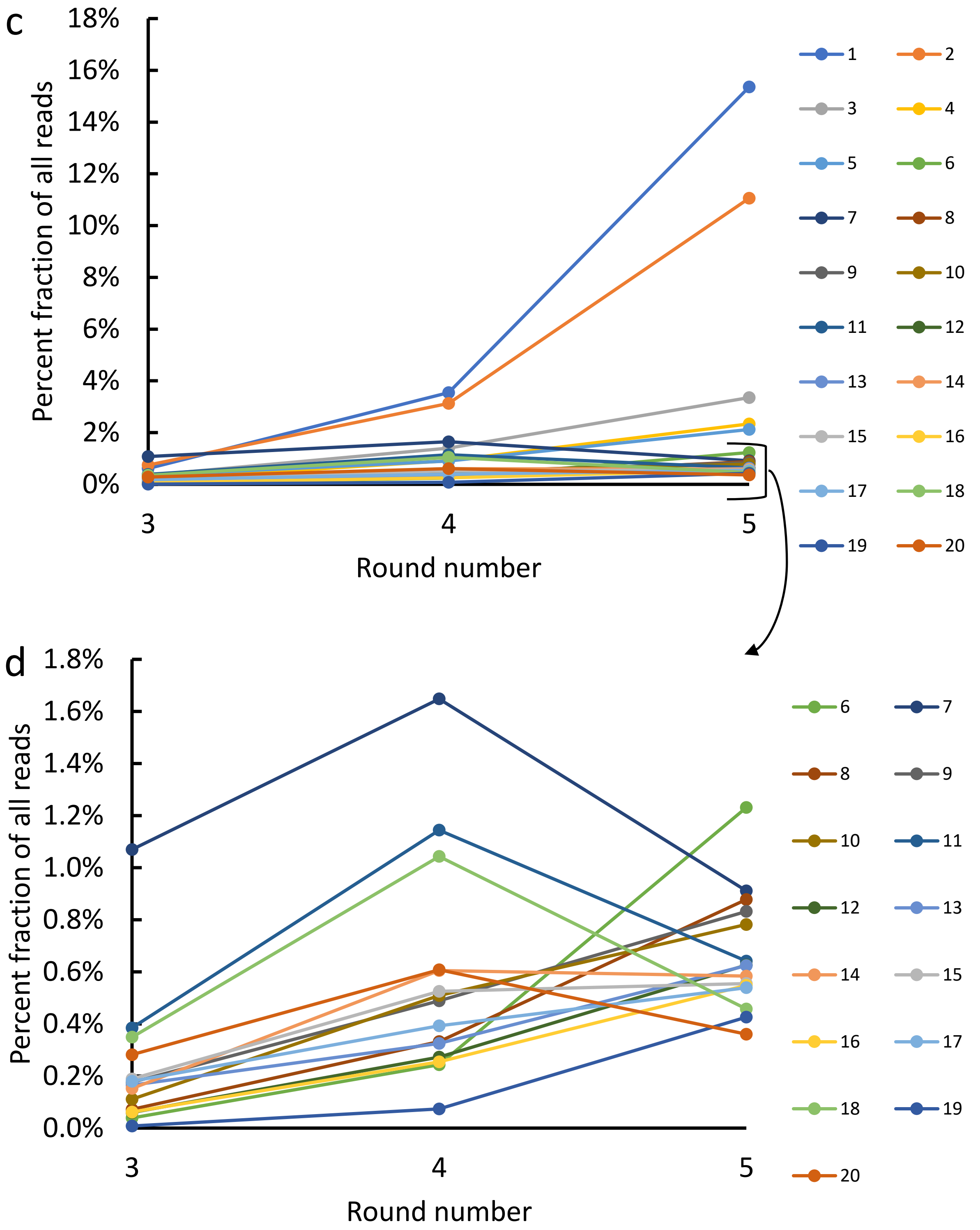
c
18%
16%
14%
12%
10%
8%
6%
4%
2%
0%
Percent fraction of all reads
3
4
5
Round number
1
2
3
4
5
6
7
8
9
10
11
12
13
14
15
16
17
18
19
20
d
1.8%
1.6%
1.4%
1.2%
1.0%
0.8%
0.6%
0.4%
0.2%
0.0%
Percent fraction of all reads
3
4
5
Round number
6
7
8
9
10
11
12
13
14
15
16
17
18
19
20

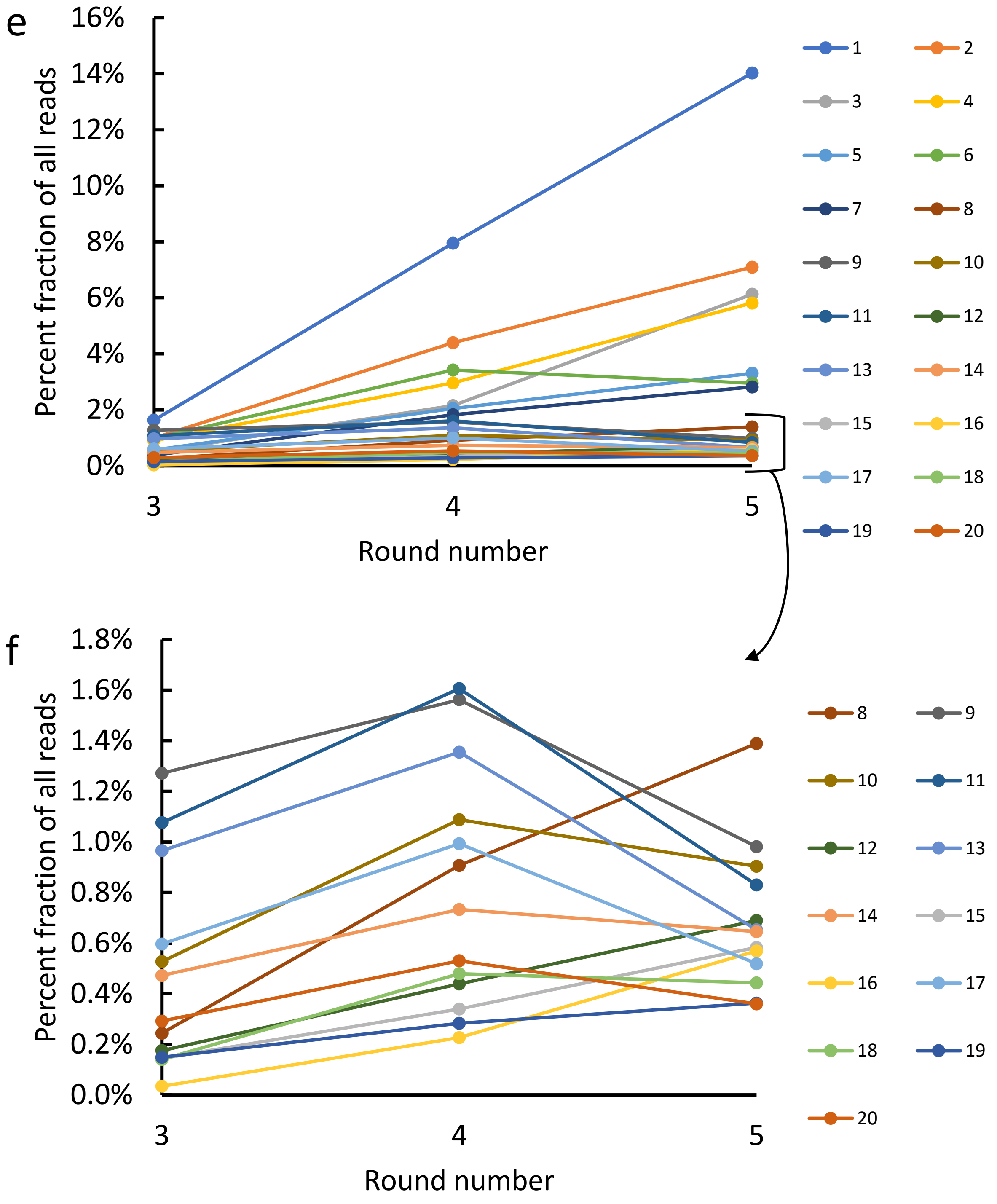

Figure S2. Recovery bar charts of DNA plots across selections rounds, representing the percentage of binding peptides in the library against immobilized protein target on beads (positive) or beads alone (negative) for both L- and D-tyrosine initiated libraries. **a.** Selection rounds against H1, showing enrichment of the libraries after 3 rounds of selection. **b.** Single selection round against H1stem and H5stem using round 3 input DNA of the selection against H1. **c** Amplification of top 20 unique sequences as a fraction of total sequence reads across the last three rounds for the L-tyrosine initiated library. **d** expansion of the baseline in c. **e** Amplification of top 20 unique sequences as a fraction of total sequence reads across the last three rounds for the D-tyrosine initiated library. **f** expansion of the baseline in e. Sequences in c through e are numbered based on abundance in the final round and can be found in fig. S3 and S4.

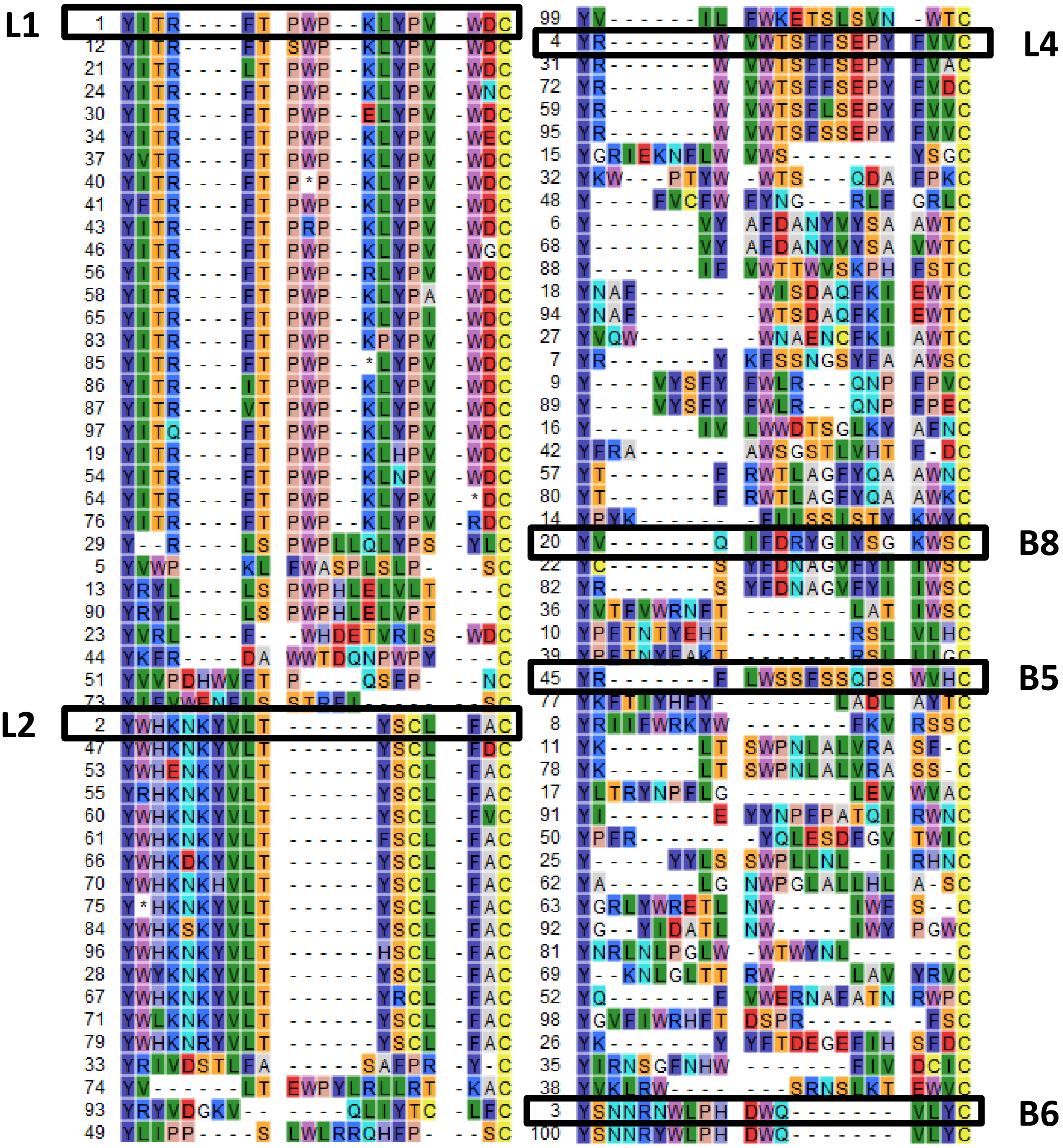

Figure S3. Sequence alignment for the 100 most enriched unique peptides in the L-Tyr initiated library at round 5 of the selection against H1. Peptides synthesized and studied in this work are highlighted.

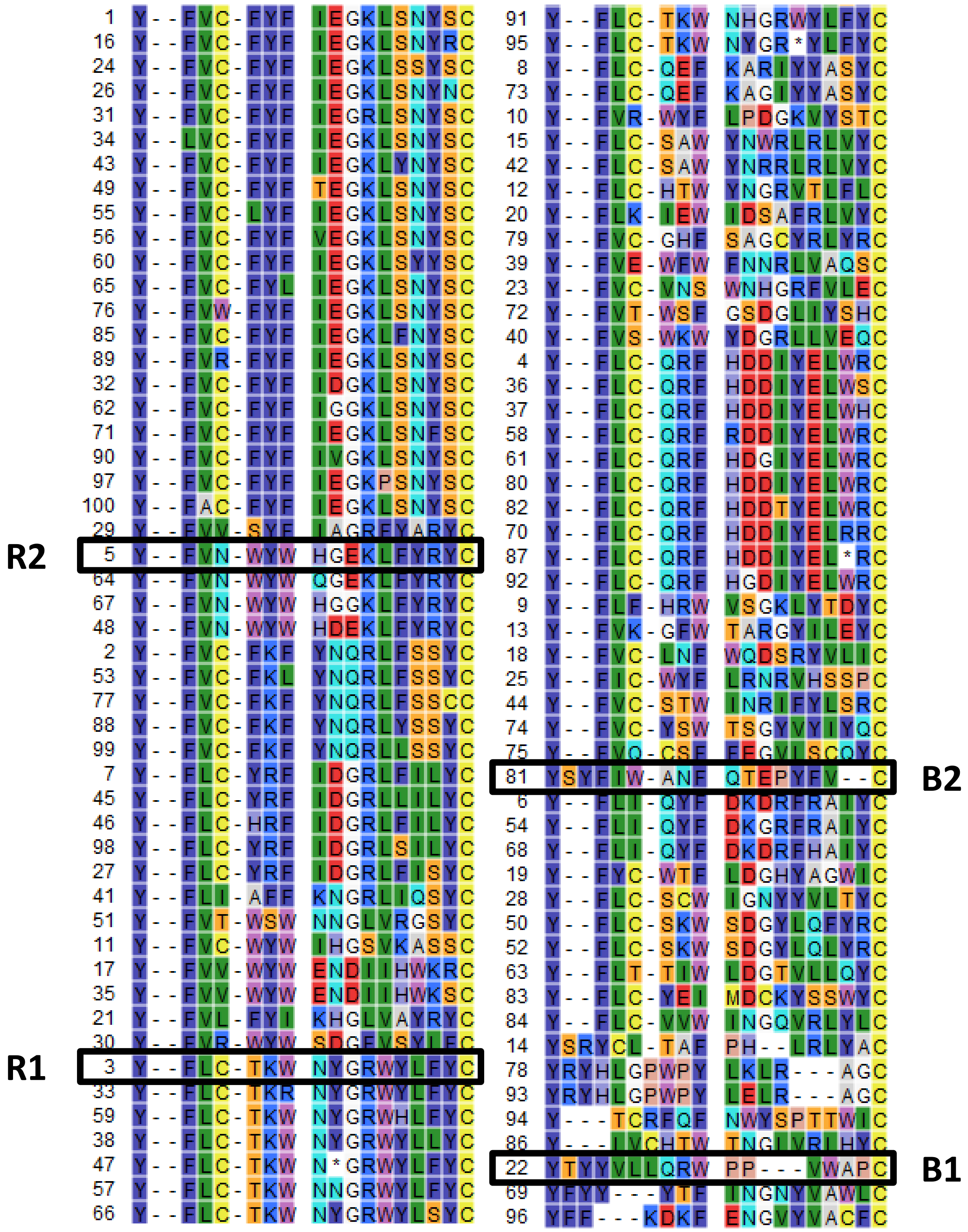

Figure S4. Sequence alignment for the 100 most enriched unique peptides in the D-Tyr initiated library at round 5 of the selection against H1. Peptides synthesized and studied in this work are highlighted.

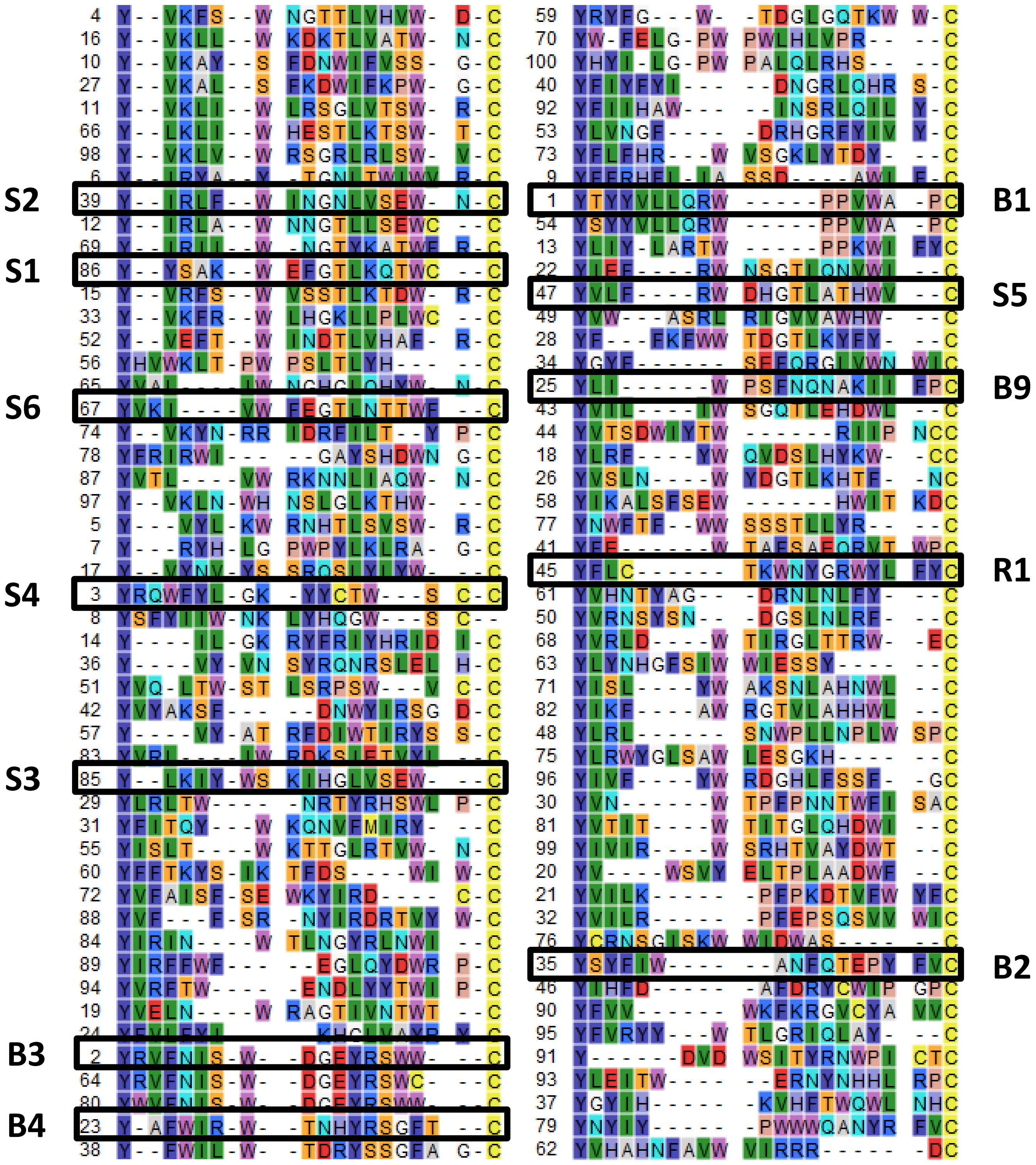

Figure S5. Sequence alignment for the 100 most enriched unique peptides in the D-Tyr initiated library of the selection against H1stem. Peptides synthesized and studied in this work are highlighted.

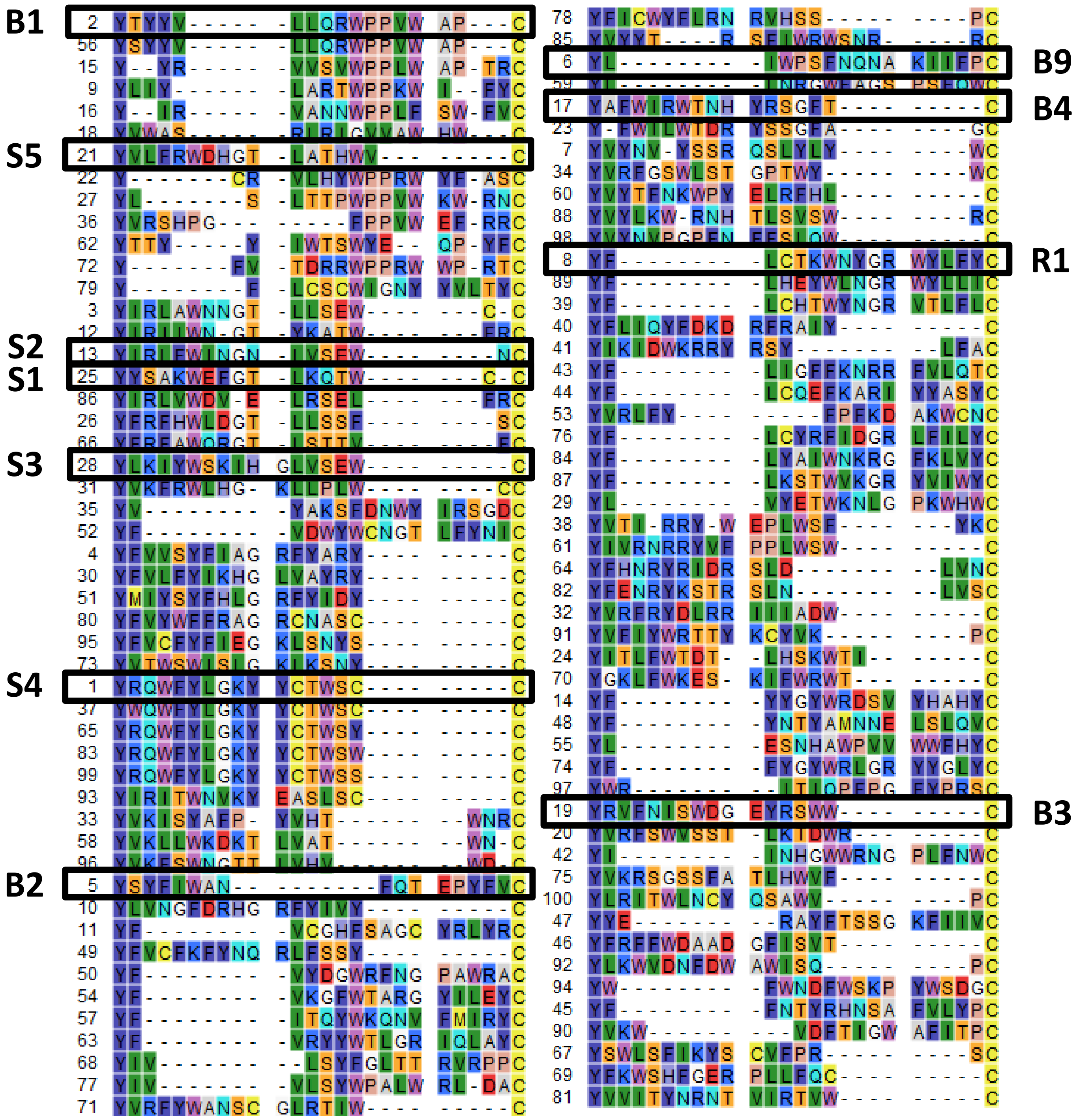

Figure S6. Sequence alignment for the 100 most enriched unique peptides in the D-Tyr initiated library at round 5 of the selection against H5stem. Peptides synthesized and studied in this work are highlighted.

`

Table S3. Sequence, molecular weight (observed and calculated), thermal shift and purity of all synthesized peptides used in the thermal shift assays (Figures S7 and S8). Peptides are coded based on sets, with S = stem binding; B = (putative) broad binding; R = RBD binding; and L = remaining sequences from the L-Tyr initiated library.

Crude	Sequence	[M+2H]$^{2+}$ observed	[M+2H]$^{2+}$ calculated	ΔTm (°C)[a]	Purity (%)[b]
**S1**	Cyclo(Ac-yYSAKWEFGTLKQTWC)CG-NH2	n.o.	1105.5	-	0
**S2**	Cyclo(Ac-yIRLFWINGNLVSEWNC)G-NH2	1112.1	1112.0	4.4	30
**S2[d]**	Cyclo(Ac-yIRLFWINGNLVSEWNC)G-NH2	1112.1	1112.0	n.d.	>95[c]
**S3**	Cyclo(Ac-yLKIYWSKIHGLVSEWC)G-NH2	1111.1	1111.1	3.8	29
**S3[d]**	Cyclo(Ac-yLKIYWSKIHGLVSEWC)G-NH2	1111.1	1111.1	4.2	>95[c]
**S4**	Cyclo(Ac-yRQWFYLGKYYC)TWSCCG-NH2	1183.5	1183.5	0.0	28
**S5**	Cyclo(Ac-yVLFRWDHGTLATHWVC)G-NH2	1100.6	1100.5	4.8	61
**S5[d]**	Cyclo(Ac-yVLFRWDHGTLATHWVC)G-NH2	1100.6	1100.5	7.8	>95[c]
**S6**	Cyclo(Ac-yVKIVWFEGTLNTTWFC)G-NH2	1102.1	1102.0	1.6	29
**S7**	Cyclo(Ac-yVIAIRPFSQLCWRWVC)G-NH2	1118.6	1118.6	0.4	19
**S8**	Cyclo(Ac-yIVLAWKGHYLESRWTC)G-NH2	1111.1	1111.1	1.6	29
**S9**	Cyclo(Ac-YSYFDVNTIFWFSPFC)CG-NH2	1118.0	1118.0	0.0	8
**B1**	Cyclo(Ac-yTYYVLLQRWPPVWAPC)G-NH2	1126.1	1126.1	2.0	66
**B1[d]**	Cyclo(Ac-yTYYVLLQRWPPVWAPC)G-NH2	1126.1	1126.1	n.d.	>90[c]
**B2**	Cyclo(Ac-ySYFIWANFQTEPYFVC)G-NH2	1137.5	1137.5	0.6	26
**B2[d]**	Cyclo(Ac-ySYFIWANFQTEPYFVC)G-NH2	1137.5	1137.5	n.d.	>95[c]
**B3**	Cyclo(Ac-yRVFNISWDGEYRSWWC)G-NH2	1182.5	1182.5	0.4	54
**B4**	Cyclo(Ac-yAFWIRWTNHYRSGFTC)G-NH2	1152.6	1152.5	2.0	60
**B4**	Cyclo(Ac-yAFWIRWTNHYRSGFTC)G-NH2	1152.6	1152.5	n.d.	>95[c]
**B5**	Cyclo(Ac-YRFLWSSFSSQPSWVHC)G-NH2	1107.0	1107.0	1.4	72
**B6**	Cyclo(Ac-YSNNRNWLPHDWQVLYC)G-NH2	1152.5	1152.5	1.6	95
**B8**	Cyclo(Ac-YVQIFDRYGIYSGKWSC)G-NH2	1091.0	1091.1	1.0	31
**B9**	Cyclo(Ac-yLIWPSFNQNAKIIFPC)G-NH2	1075.6	1075.5	3.6	55
**R1**	Cyclo(Ac-yFLC)TKWNYGRWYLFYCG-NH2	1211.6	1211.6	1.4	20
**R2**	Cyclo(Ac-yFVNWYWHGEKLFYRYC)G-NH2	1235.6	1235.6	2.2	26
**R4**	Cyclo(Ac-YYSWFEATGRFRAIFSC)G-NH2	1100.5	1100.5	1.4	34

**R5**	Cyclo(Ac-YYKALWINATFYQSLVC)G-NH2	1090.1	1090.0	1.2	9
**L1**	Cyclo(Ac-YITRFTPWPKLYPVWDC)G-NH2	1141.6	1141.6	3.8	>95[c]
**L2**	Cyclo(Ac-YWHKNKYVLTYSC)LFAAG-NH2	1102.1	1102.0	1.0	>95[c]
**L4**	Cyclo(Ac-YRWVWTSFFSEPYFVVC)G-NH2	1156.6	1156.5	3.4	>95[c]

[a] T_m change compared to DMSO control at estimated 5μM peptide concentration, $\Delta T_m = T_m$[peptide]-T_m[dmso]
[b] Purity is determined by UV absorption at 215 nm using the ratio of area under the curve for the desired peptide compared to all peptide peaks
[c] Purified peptides that were used for all subsequent experiments
[d] Peptides used in the HDX-MS experiment

n.o. not observed
n.d. not determined

Table S4. List of biotin- and fluorescein-labeled peptides and peptide controls used in this work (where y = D-tyrosine, p = 2-[2-[2-(Fmoc-amino)ethoxy]ethoxy]acetic acid, a = L-azidohomoalanine, FAM = DBCO-activated fluorescein 6-isomer)

Peptide	Sequence	$[M+2H]^{2+}$ observed	$[M+2H]^{2+}$ calculated
**Biotin-L2**	Cyclo(Ac-YWHKNKYVLTYSC)LFAAGGK$_{biotin}$G-NH2	1336.2	1336.1
**Biotin-L4**	Cyclo(Ac-YRWVWTSFFSEPYFVVC)GGK$_{biotin}$G-NH2	1390.7	1390.6
**Biotin-S3**	Cyclo(Ac-yLKIYWSKIHGLVSEWC)GGK$_{biotin}$G-NH2	1345.2	1345.2
**Biotin-S5**	Cyclo(Ac-yVLFRWDHGTLATHWVC)GGK$_{biotin}$G-NH2	1334.6	1334.6
**L2-FAM**	Cyclo(Ac-YWHKNKYVLTYSC)LFAApa$_{FAM}$GDYKDDDDKG-NH2	2102.8	2102.4
**L4-FAM**	Cyclo(Ac-YRWVWTSFFSEPYFVVC) pa$_{FAM}$GDYKDDDDKG-NH2	2157.3	2156.9
**S3-FAM**	Cyclo(Ac-yLKIYWSKIHGLVSEWC) pa$_{FAM}$GDYKDDDDKG-NH2	2111.7	2111.5
**S5-FAM**	Cyclo(Ac-yVLFRWDHGTLATHWVC) pa$_{FAM}$GDYKDDDDKG-NH2	2101.3	2100.9
**S3 linear**	Ac-yLKIYWSKIHGLVSEWAG-NH2	1096.1	1096.1
**S3 scrambled**	Cyclo(Ac-yIHIYWWSVLSEGKKLC)G-NH2	1111.1	1111.1
**L4 linear**	Ac-YRWVWTSFFSEPYFVVAG-NH2	1141.6	1141.6
**L4 scrambled**	Cyclo(Ac-YFEWYRFWVTPSVSVGC)G-NH2	1156.6	1156.5

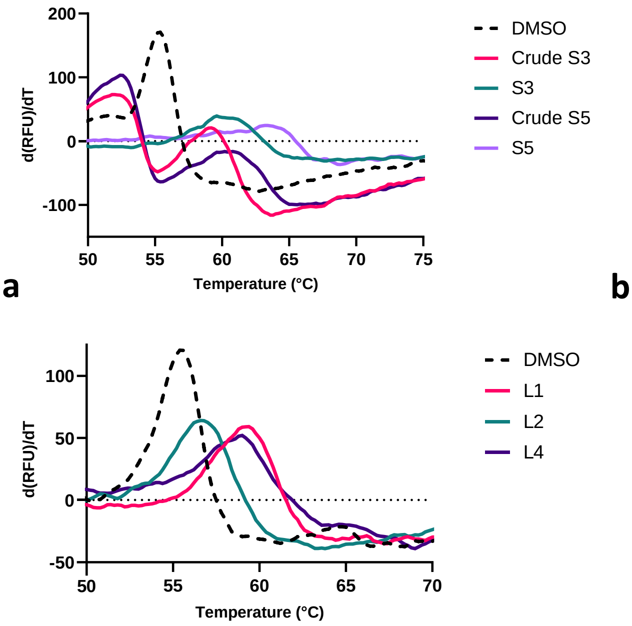

Figure S7. Thermal shift analysis of peptides binding to H1. Thermal denaturation curves of H1 with or without the addition of peptides are shown. **a.** Comparison of observed thermal stability increase for peptides S3 and S5, either as crude mixtures (estimated 5 µM) or purified peptides (1 µM). Clear change in thermal stability can be observed in both cases, with the peak at the highest temperature for the crude peptides being used for calculation of ΔT_m (table S4). **b.** Thermal denaturation curves of H1 with the addition of purified peptides L1, L2 and L4 at 1 µM.

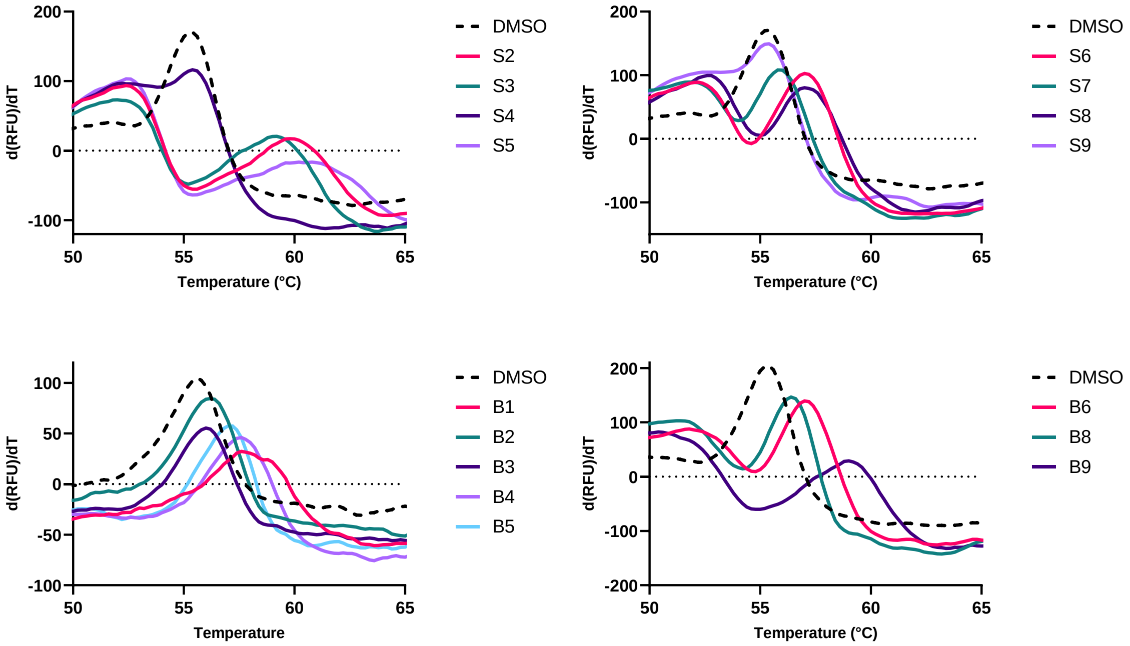

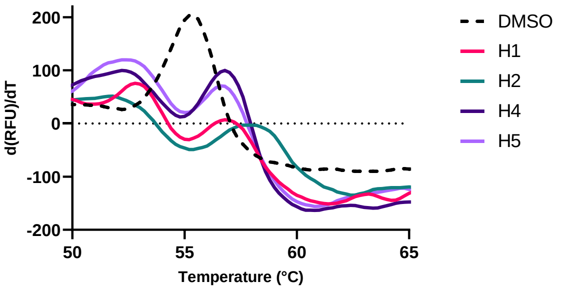

Figure S8. Thermal shift analysis of crude peptide binding to H1. Thermal denaturation curves of H1 with the addition of crude peptide mixtures (estimated 5 μM) or without (DMSO).

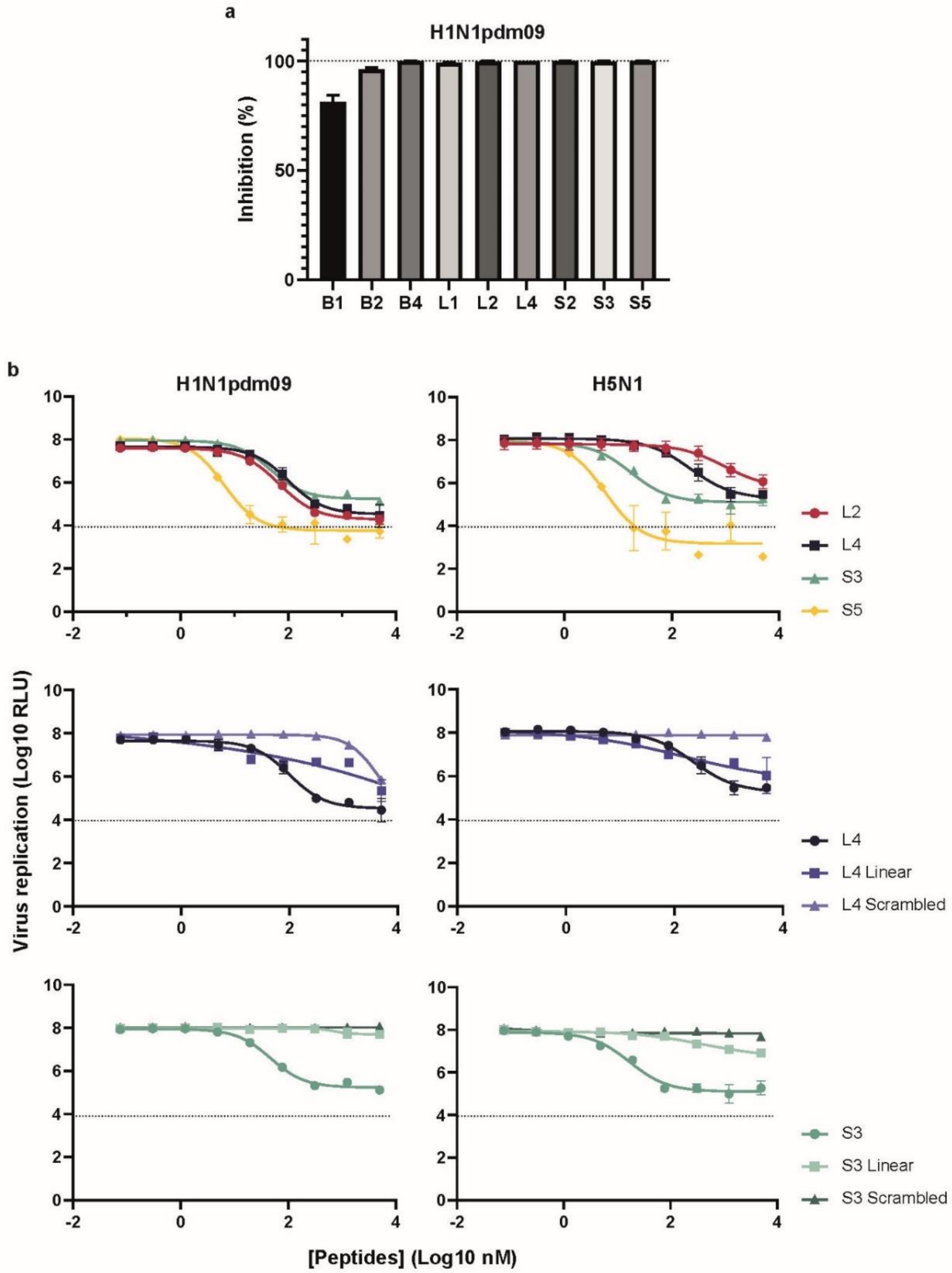

Figure S9. Dose-dependent neutralizing effect of peptides on H1N1pdm09 and H5N1. (a) Cells were infected with H1N1pdm09 in the absence or presence of a panel of macrocyclic peptides at 5 µM. Inhibition was calculated using the luciferase assay relative to cells treated without peptide inhibitors. (b) Replication of H1N1pdm09 and H5N1 virus in the presence of serial dilutions of peptides (starting at 5 µM, 4-fold dilutions) as determined with the luciferase assay. Upper panels: luciferase levels in the presence of candidate macrocyclic RaPID peptides. Middle and lower panels: luciferase levels of cells treated with control peptides. Linear = linearized versions of the original peptides. Scrambled = same amino acids as in the original peptides, but in a random order. Dotted lines represent background luciferase signal. Representative experiments (out of two performed) are shown as mean±SD (n=3).

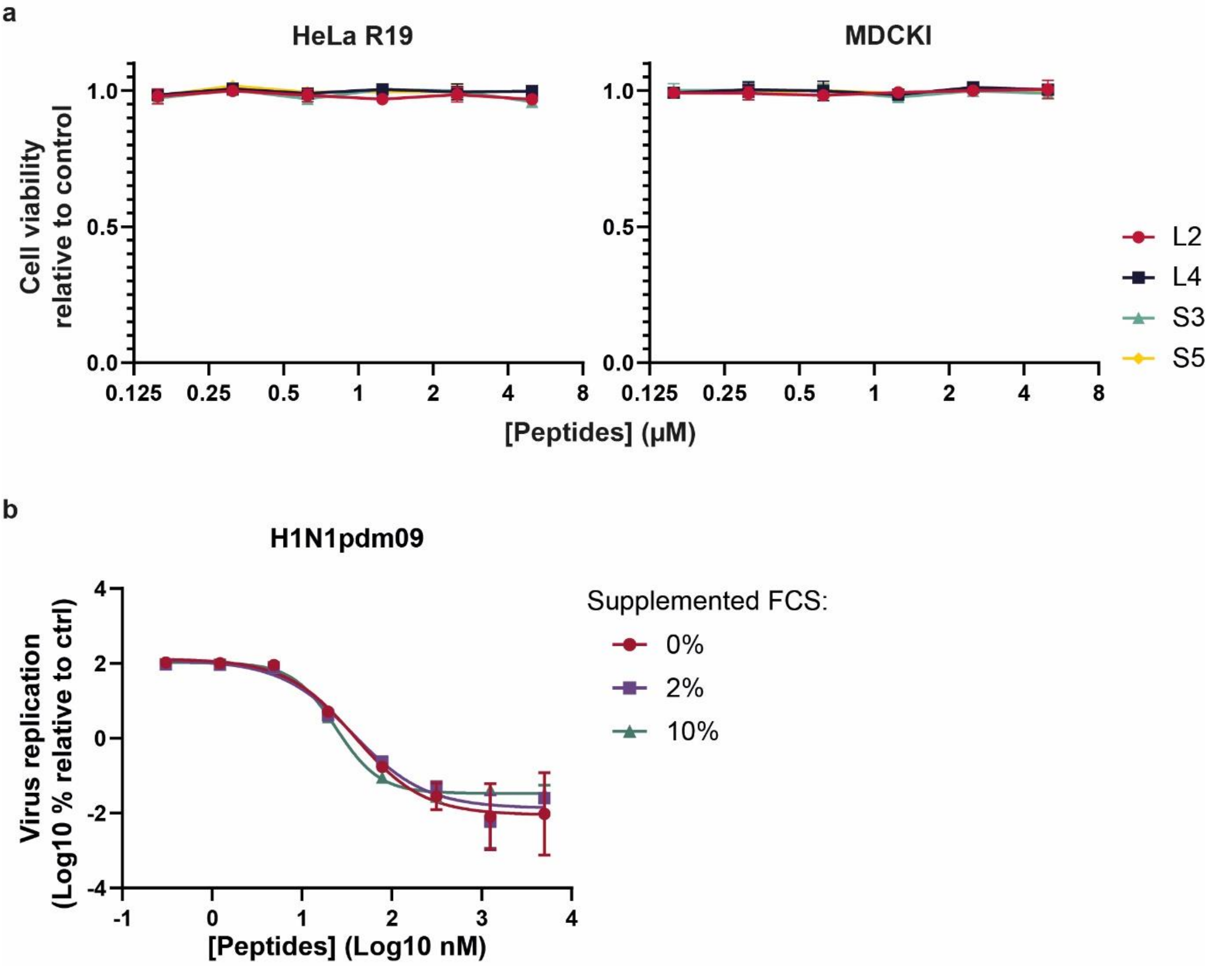

Figure S10. Peptides are non-cytotoxic and inhibition is unaffected by serum proteins. (a) Peptides do not affect viability of HeLa and MDCKI cells. Cells were treated with peptides for 72 h prior to analysis using Wst-1 proliferation reagent. Viability is plotted relative to untreated control wells. (b) Infection with H1N1pdm09 was performed in presence of varying amounts of fetal calf serum (FCS) with peptide S5 inhibition. Replication was measured as luciferase activity and plotted as a percentage of control without peptides. Data are shown as mean±SD (n=3).

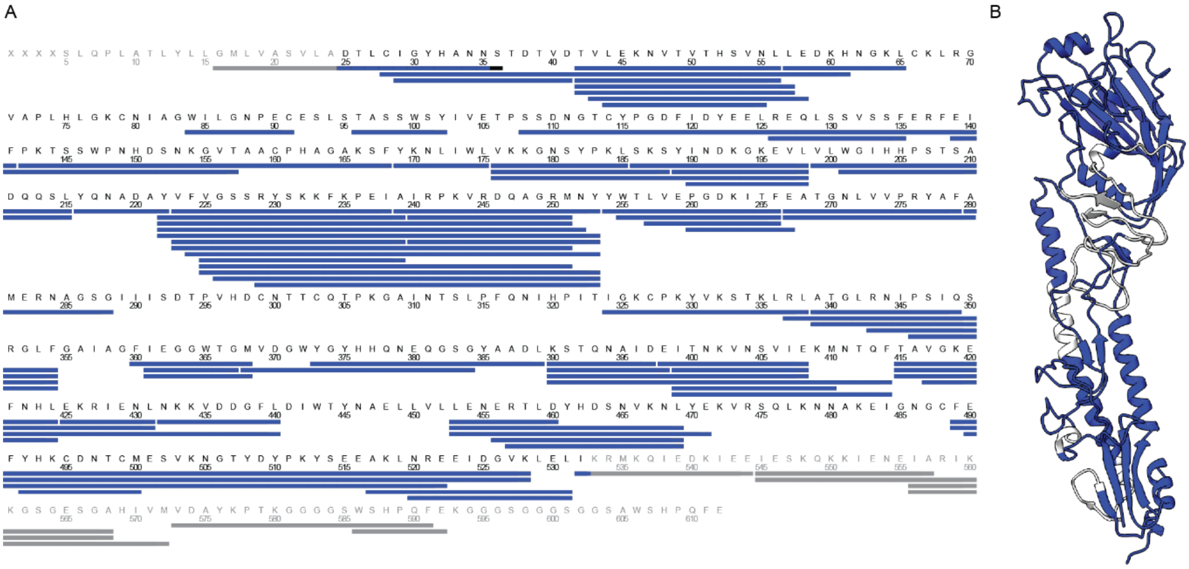

Figure S11. HDX-MS Coverage map of HA. (A) Complete sequence of recombinant soluble H1 HA ectodomain of H1N1pdm09 used in this study. Blue bars represent HA-specific peptides detected after HDX labeling experiment, gray bars correspond with peptides derived from trimerization domain and/or purification tags. (B) Visualization of the peptides observed in context of the HA monomer, blue indicate the observed parts of HA.

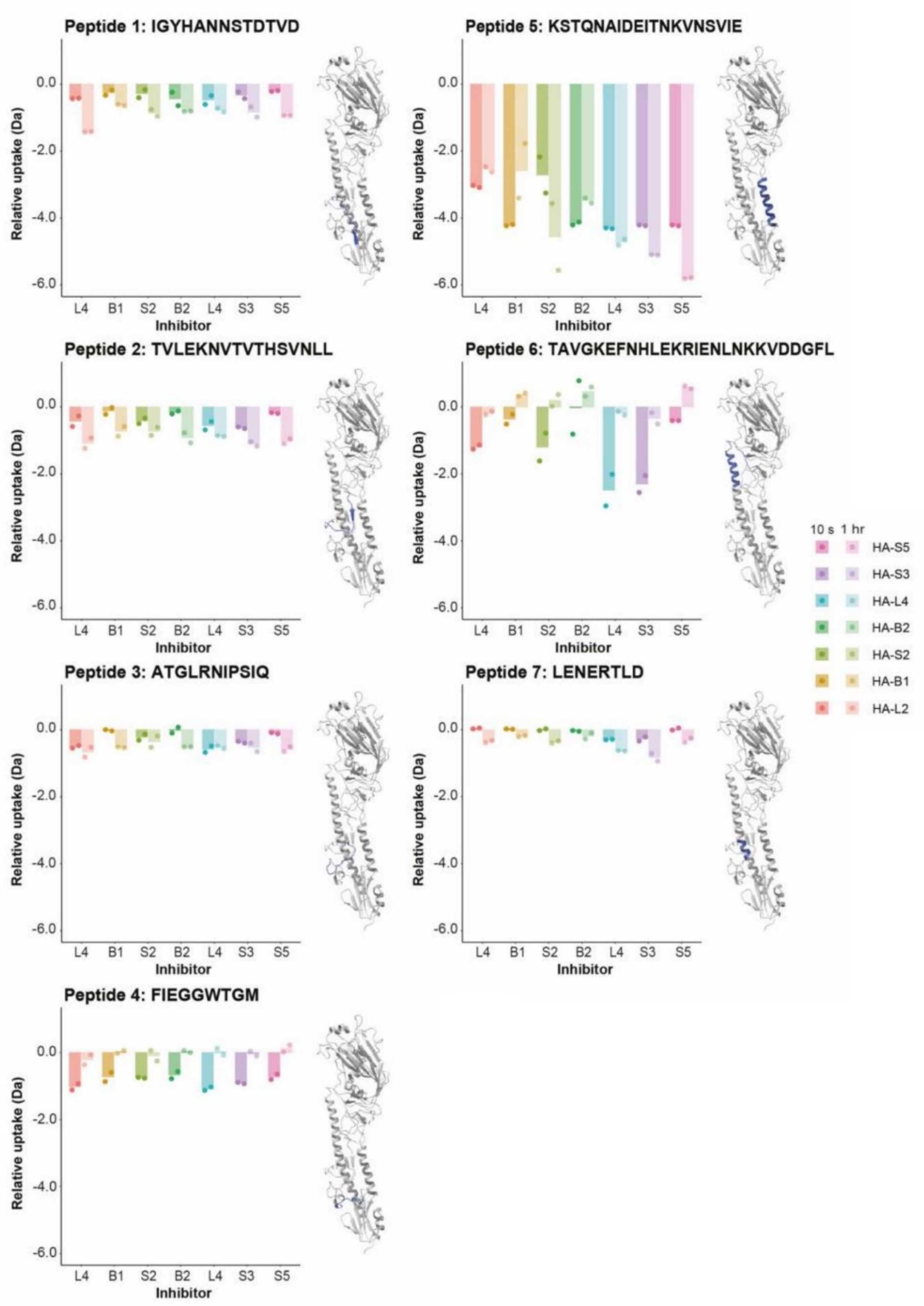

Figure S12. Deuterium uptake differences in per highlighted peptide relative to unbound HA. Results for different peptide inhibitors are shown in different colors, for either 10s (left bar) or 1 hour incubation (right bar). On the right side of the plots, the considered digested peptide fragments are mapped in blue on an atomic model of the HA monomer.

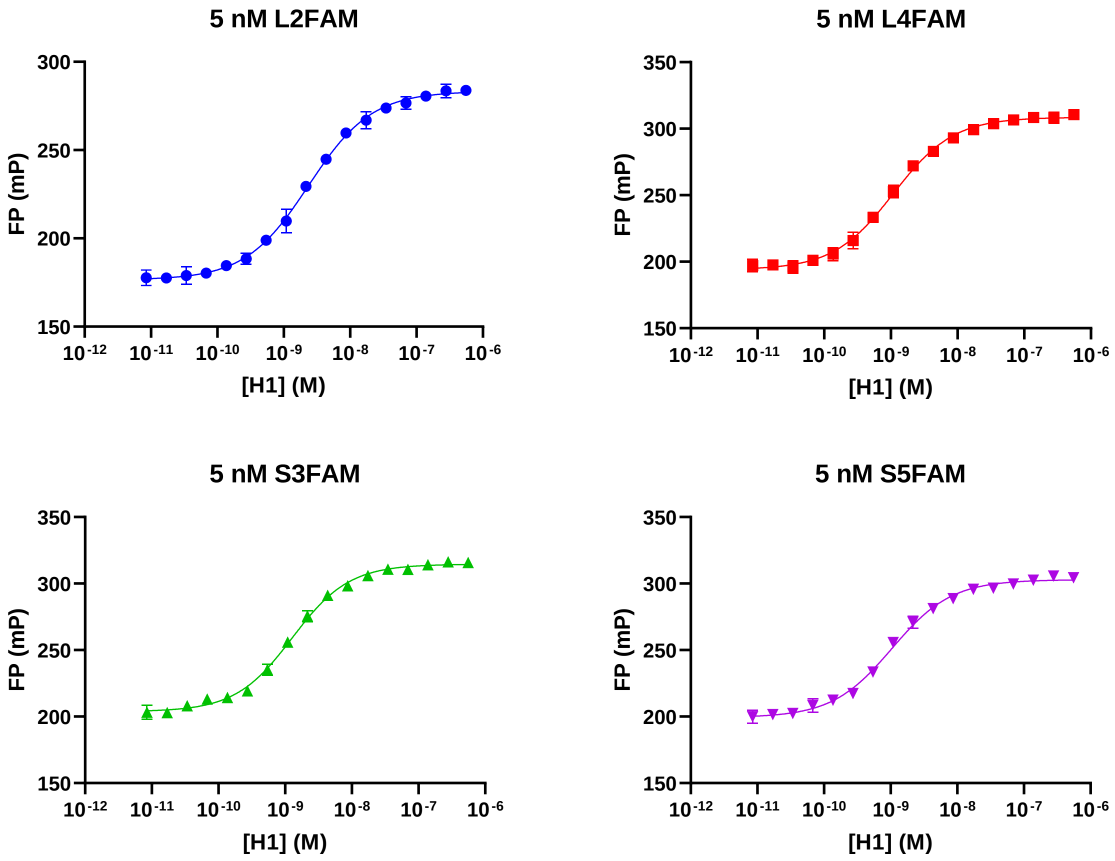

Figure S13. Fluorescence polarization measurements from 1:1 serial dilution of H1 with 5 nM FAM-labelled peptides.

Table S5. Binding affinities and Hillslope of fluorescence polarization assay at 5 nM peptide concentration.

	**Kd (nM)**	**95% CI Kd (nM)**	**Hillslope**	**95% CI Hillslope**
**L2FAM**	2.4	2.1-2.6	0.91	0.83-1.00
**L4FAM**	1.1	1.0-1.2	0.96	0.87-1.01
**S3FAM**	1.3	1.2-1.4	1.02	0.92-1.13
**S5FAM**	1.0	0.9-1.2	0.97	0.87-1.08

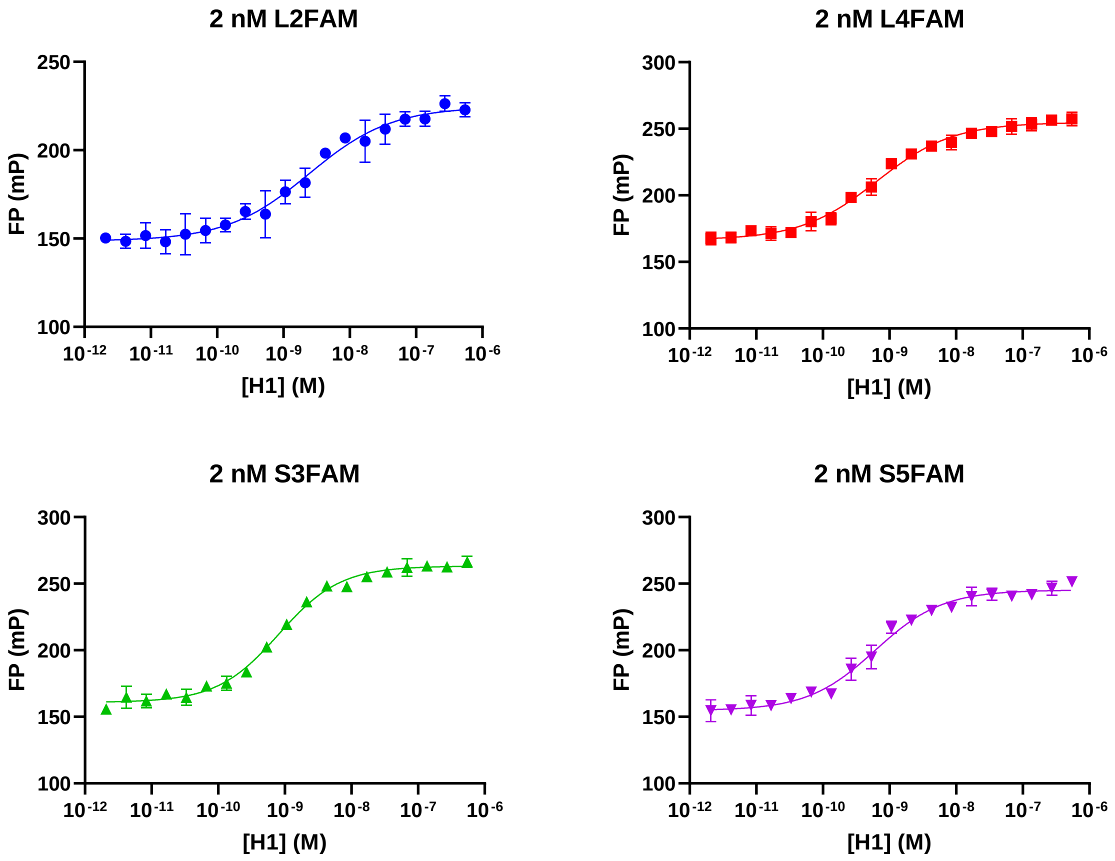

Figure S14. Fluorescence polarization measurements from 1:1 serial dilution of H1 with 2 nM FAM-labelled peptides.

Table S6. Binding affinities and Hillslope of fluorescence polarization assay at 2 nM peptide concentration.

	**Kd (nM)**	**95% CI Kd (nM)**	**Hillslope**	**95% CI Hillslope**
**L2FAM**	2.4	1.6-3.8	0.70	0.51-0.94
**L4FAM**	0.6	0.5-0.8	0.76	0.64-0.90
**S3FAM**	0.8	0.7-0.9	0.94	0.82-1.08
**S5FAM**	0.6	0.5-0.8	0.86	0.70-1.05

Table S7. Binding affinities (K_{d1}) of different hemagglutinin protein constructs against immobilized peptides in nanomolar.

	**L4**	**S3**	**S5**
**H1**	20.8 ± 1.4	16.8 ± 0.6	8.6 ± 1.1
**H1stem**	2.6 ± 0.1	37.2 ± 18.8	11.3 ± 2.5
**H1stem I375F**	-	-	-
**H1stem I463V**	7.8 ± 0.2	12.0 ± 0.2	23.8 ± 1.3
**H5stem**	-	160 ± 5	119 ± 3
**H3**	-	-	-
**H9**	-	-	-

[-] No binding observed

Table S8 Overview of SPR data for different protein constructs binding immobilized peptides

Peptide	Protein	K_{on1} (M^{-1} s^{-1})	K_{off1} (s^{-1})	Kd1 (nM)	SE Kd1 (nM)	Kon2 (RU^{-1} s^{-1})	Koff2 (s^{-1})	Rmax (RU)	X^2 (RU^2)
**L4**	*H1*	$5.01x10^{4}$	$1.04x10^{-3}$	20.8	1.4	$3.44x10^{-3}$	$2.32x10^{-3}$	595.1	1.21
	H1stem	$9.78x10^{4}$	$2.55x10^{-4}$	2.6	0.1	$3.42x10^{-5}$	$5.34x10^{-6}$	268.6	2.29
	H1stem I375F	-	-	-	-	-	-	-	-
	H1stem I463V	$7.75x10^{4}$	$6.06x10^{-4}$	7.8	0.2	$4.98x10^{-5}$	$1.43x10^{-8}$	276.3	0.812
	H5stem	-	-	-	-	-	-	-	-
	H3	-	-	-	-	-	-	-	-
	H9	-	-	-	-	-	-	-	-
**S3**	*H1*	$3.90x10^{3}$	$6.56x10^{-5}$	16.8	0.6	$1.47x10^{-7}$	$1.32x10^{-3}$	963.7	5.01
	H1stem	$4.34x10^{5}$	$1.61x10^{-2}$	37.2	18.8	$7.51x10^{-5}$	$2.15x10^{-5}$	163.6	13.3
	H1stem I375F	-	-	-	-	-	-	-	-
	H1stem I463V	$6.26x10^{4}$	$7.53x10^{-4}$	12.0	0.2	$4.01x10^{-5}$	$4.81x10^{-7}$	152.7	0.417
	H5stem	$4.40x10^{3}$	$7.03x10^{-4}$	160	5	$6.59x10^{-10}$	$2.38x10^{-3}$	259.4	0.699
	H3	-	-	-	-	-	-	-	-
	H9	-	-	-	-	-	-	-	-
**S5**	*H1fl*	$3.83x10^{4}$	$3.28x10^{-4}$	8.6	1.1	$4.15x10^{-5}$	$5.91x10^{-5}$	192.7	0.189
	H1stem	$7.79x10^{4}$	$8.83x10^{-4}$	11.3	2.5	$1.09x10^{-5}$	$2.72x10^{-5}$	160.2	9.45
	H1stem I375F	-	-	-	-.	-	-	-	-
	H1stem I463V	$3.34x10^{4}$	$7.96x10^{-4}$	23.8	1.3	$3.18x10^{-5}$	$7.58x10^{-5}$	185.9	0.574
	H5stem	$2.20x10^{4}$	$2.62x10^{-3}$	119	3	$3.81x10^{-5}$	$1.08x10^{-4}$	94.12	0.126
	H3	-	-	-	-	-	-	-	-
	H9	-	-	-	-	-	-	-	-

[-] No binding was observed

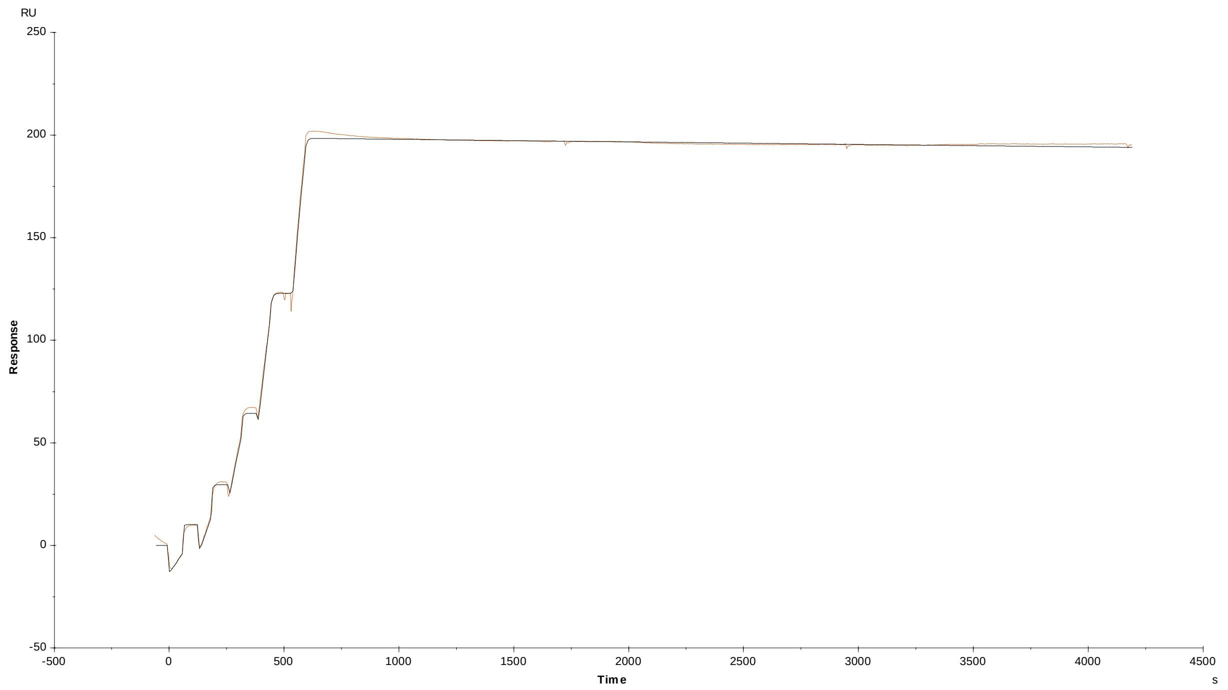

Figure S15. Single-cycle kinetic analysis of the interaction between immobilized peptide **L4** and **H1** by SPR, curve fitting with bivalent analyte model. Response was measured at 5 different protein concentrations (3 nM, 6 nM, 12 nM, 25 nM and 50 nM).

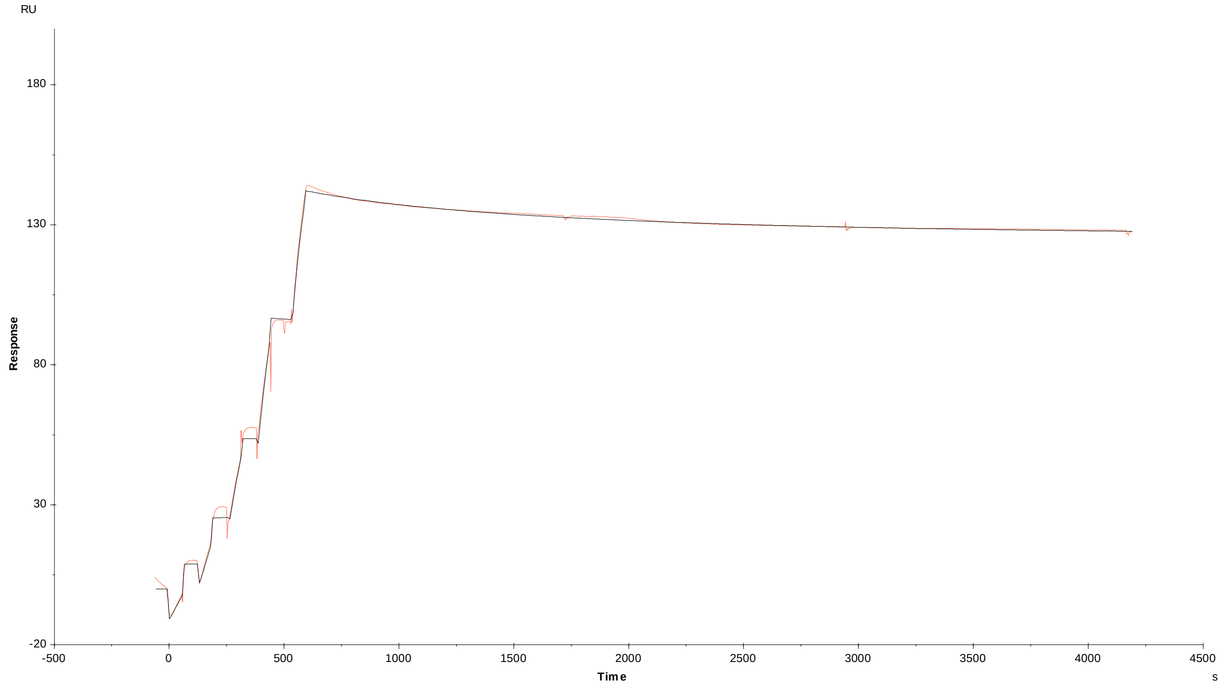

Figure S16. Single-cycle kinetic analysis of the interaction between immobilized peptide **L4** and **H1stem** by SPR, curve fitting with bivalent analyte model. Response was measured at 5 different protein concentrations (3 nM, 6 nM, 12 nM, 25 nM and 50 nM).

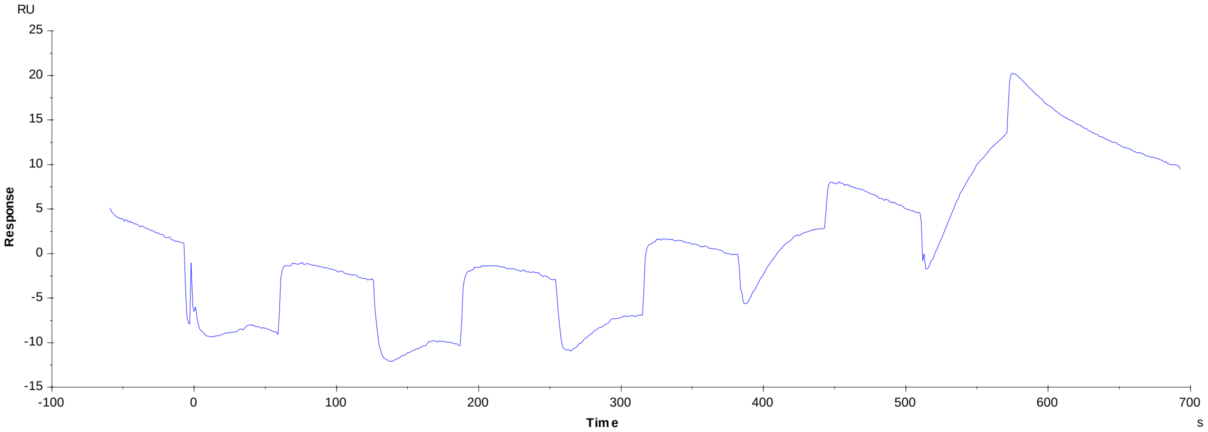

Figure S17. Single-cycle kinetic analysis of the interaction between immobilized peptide **L4** and **H1stem I375F** by SPR in which no binding was observed. Response was measured at 5 different protein concentrations (6 nM, 12 nM, 25 nM, 50 nM and 100 nM).

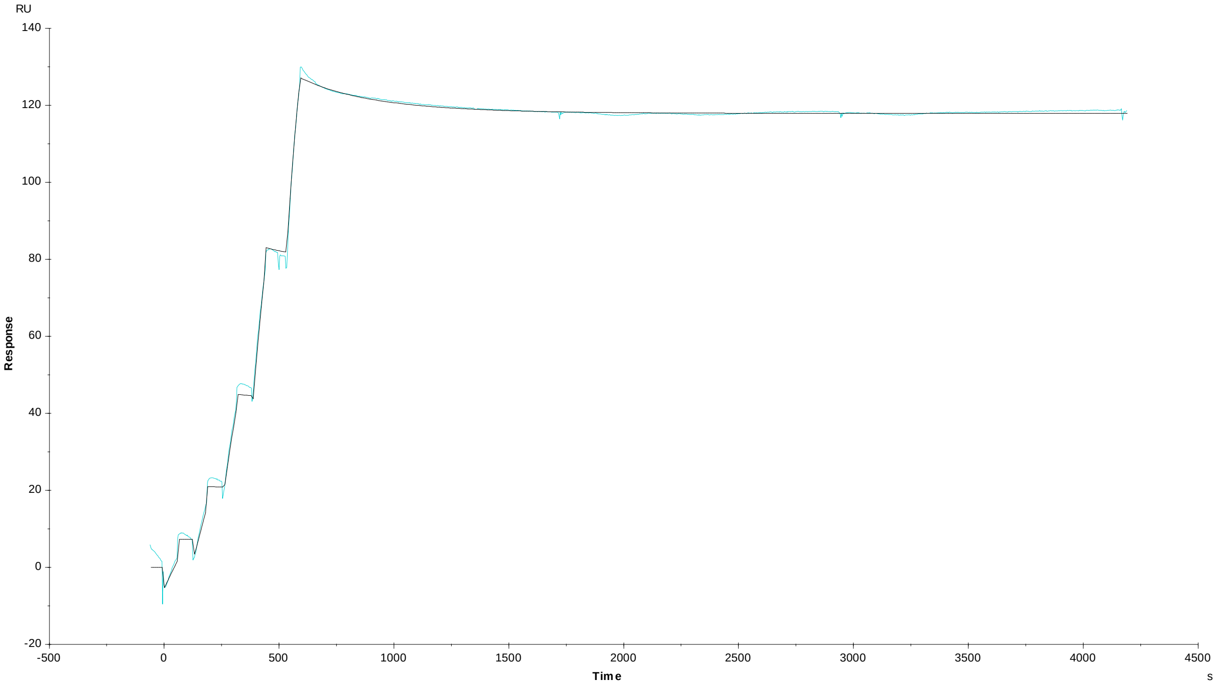

Figure S18. Single-cycle kinetic analysis of the interaction between immobilized peptide **L4** and **H1stem I463V** by SPR, curve fitting with bivalent analyte model. Response was measured at 5 different protein concentrations (3 nM, 6 nM, 12 nM, 25 nM and 50 nM).

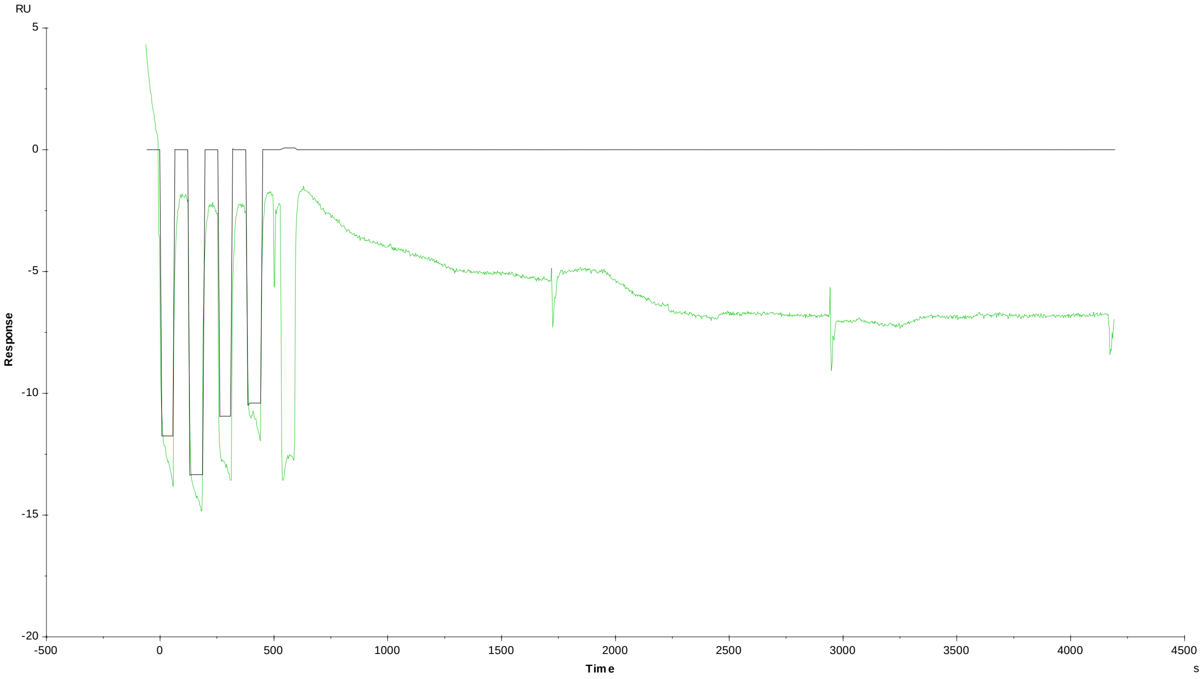

Figure S19. Single-cycle kinetic analysis of the interaction between immobilized peptide **L4** and **H5stem** by SPR in which no binding was observed. Response was measured at 5 different protein concentrations (20 nM, 30 nM, 40 nM, 50 nM and 60 nM).

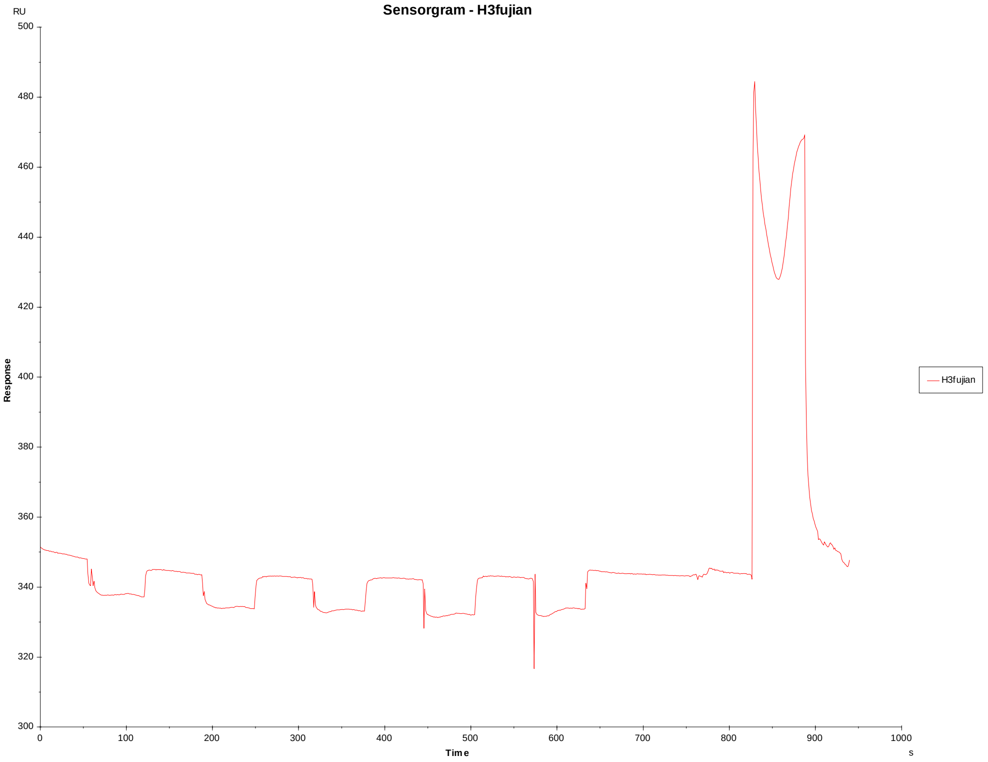

Figure S20. Single-cycle kinetic analysis of the interaction between immobilized peptide **L4** and **H3** by SPR in which no binding was observed. Response was measured at 5 different protein concentrations (6 nM, 12 nM, 25 nM, 50 nM and 100 nM).

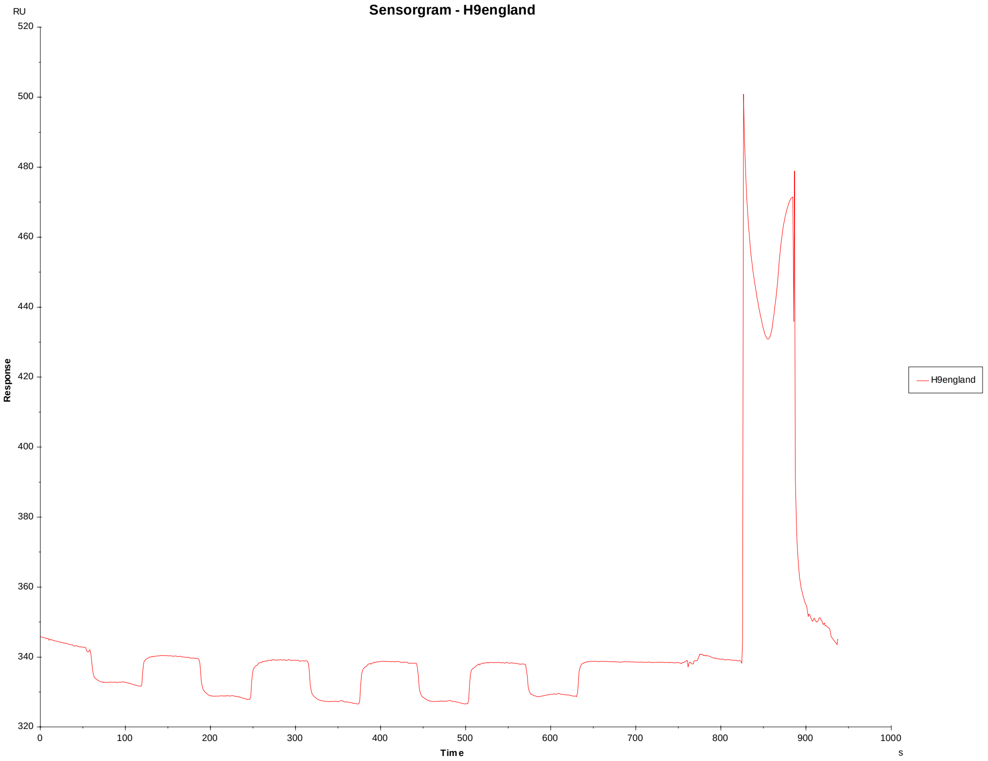

Figure S21. Single-cycle kinetic analysis of the interaction between immobilized peptide **L4** and **H9** by SPR in which no binding was observed. Response was measured at 5 different protein concentrations (3 nM, 6 nM, 12 nM, 25 nM and 50 nM).

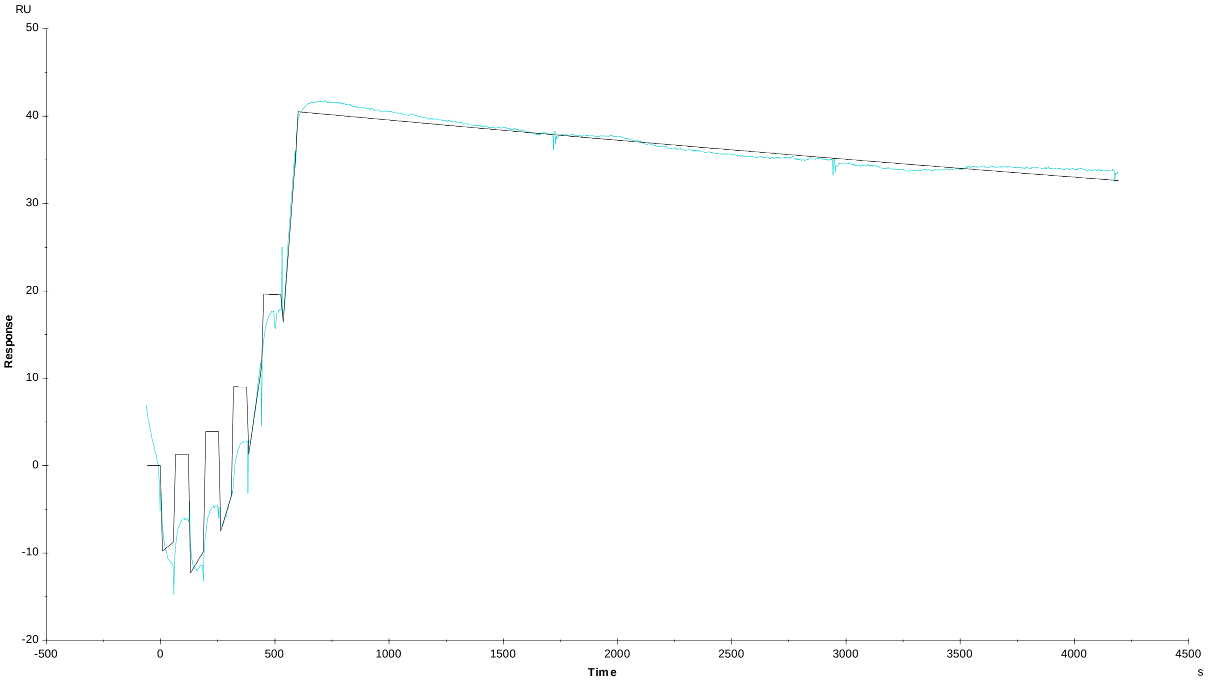

Figure S22. Single-cycle kinetic analysis of the interaction between immobilized peptide **S3** and **H1** by SPR, curve fitting with bivalent analyte model. Response was measured at 5 different protein concentrations (3 nM, 6 nM, 12 nM, 25 nM and 50 nM).

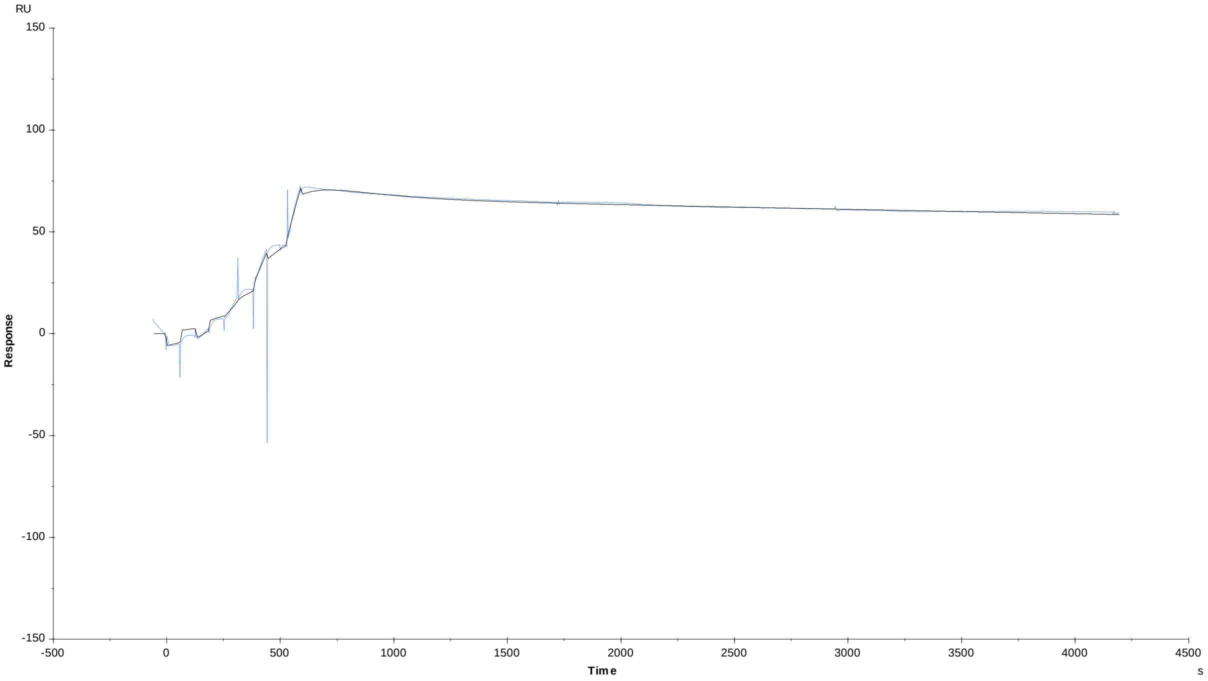

Figure S23. Single-cycle kinetic analysis of the interaction between immobilized peptide **S3** and **H1stem** by SPR, curve fitting with bivalent analyte model. Response was measured at 5 different protein concentrations (3 nM, 6 nM, 12 nM, 25 nM and 50 nM).

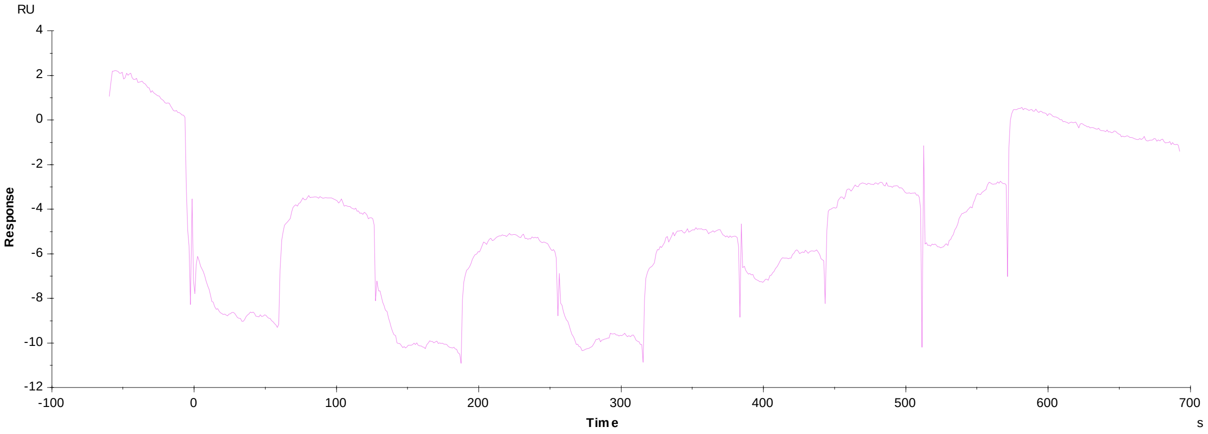

Figure S24. Single-cycle kinetic analysis of the interaction between immobilized peptide **S3** and **H1stem I375F** by SPR in which no binding was observed. Response was measured at 5 different protein concentrations (6 nM, 12 nM, 25 nM, 50 nM and 100 nM).

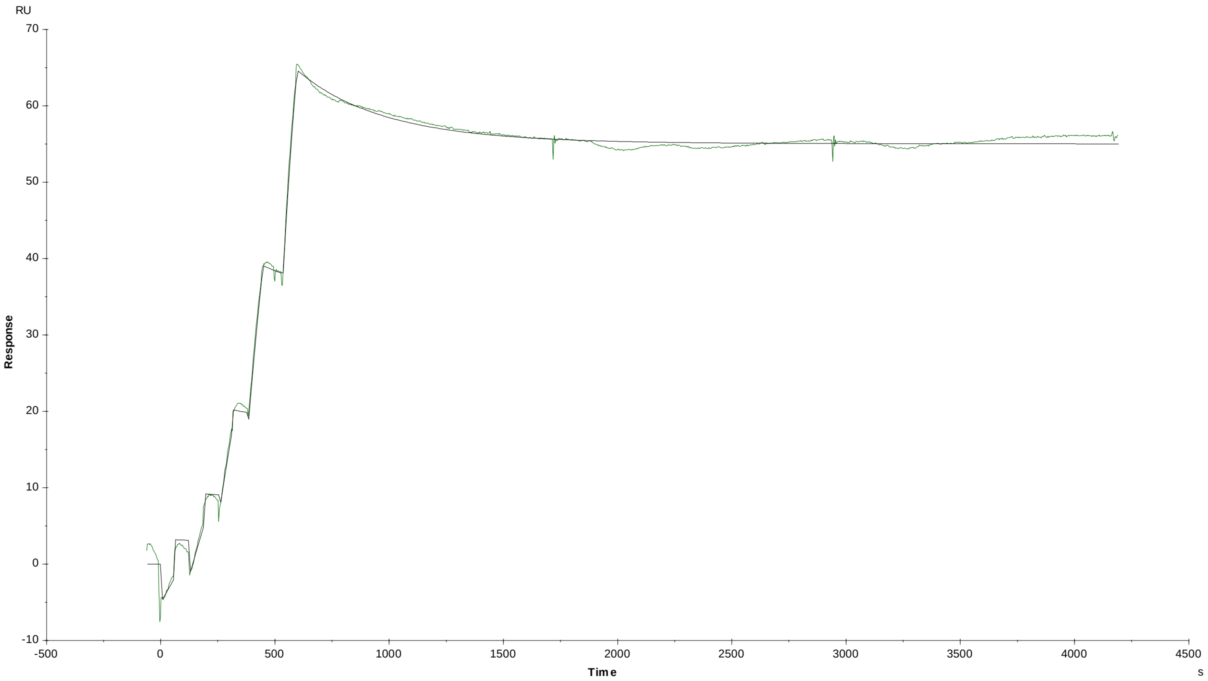

Figure S25. Single-cycle kinetic analysis of the interaction between immobilized peptide **S3** and **H1stem I463V** by SPR, curve fitting with bivalent analyte model. Response was measured at 5 different protein concentrations (3 nM, 6 nM, 12 nM, 25 nM and 50 nM).

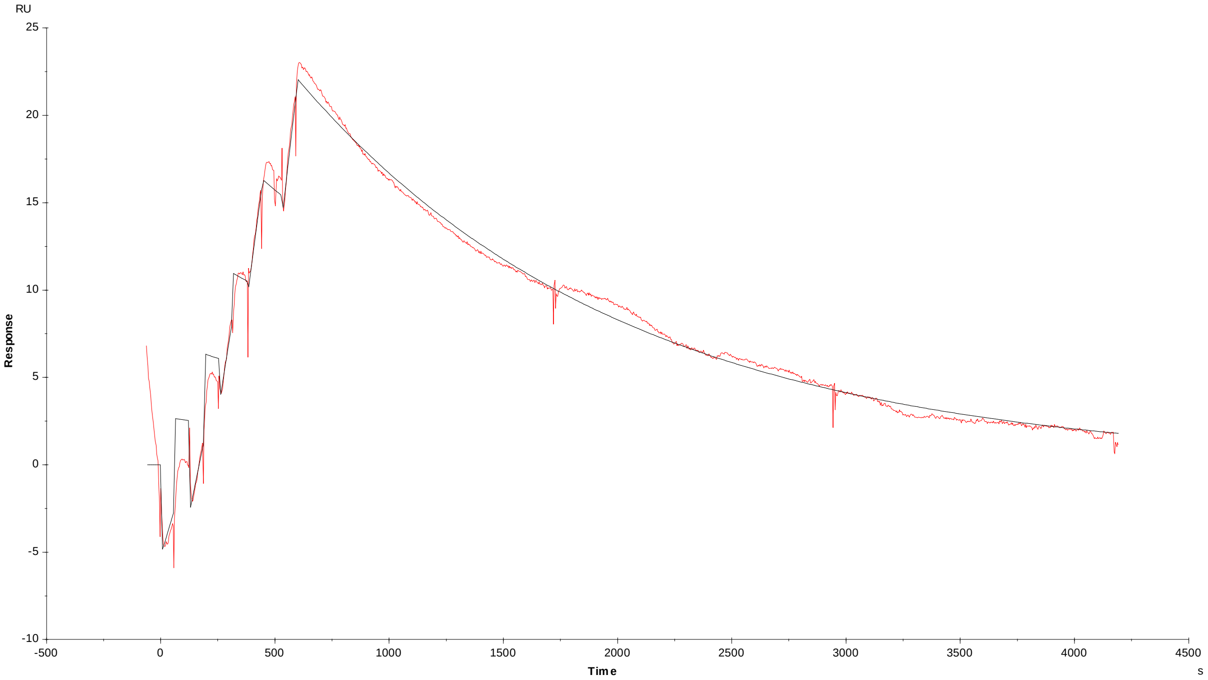

Figure S26. Single-cycle kinetic analysis of the interaction between immobilized peptide **S3** and **H5stem** by SPR, curve fitting with bivalent analyte model. Response was measured at 5 different protein concentrations (20 nM, 30 nM, 40 nM, 50 nM and 60 nM).

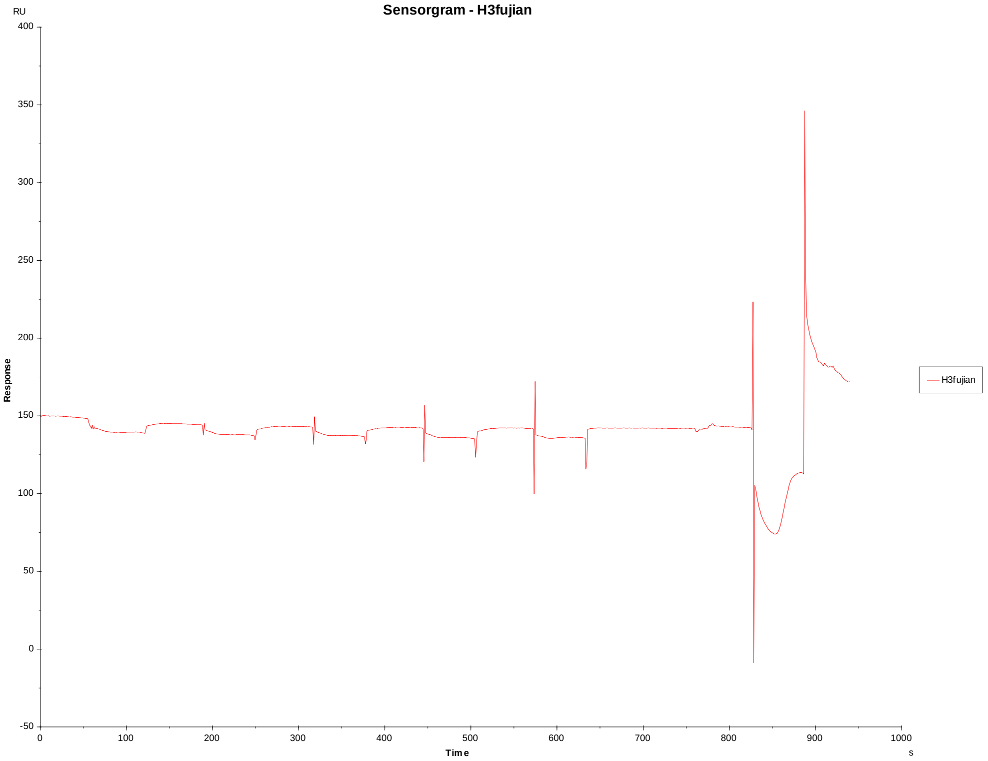

Figure S27. Single-cycle kinetic analysis of the interaction between immobilized peptide **S3** and **H3** by SPR in which no binding was observed. Response was measured at 5 different protein concentrations (6 nM, 12 nM, 25 nM, 50 nM and 100 nM).

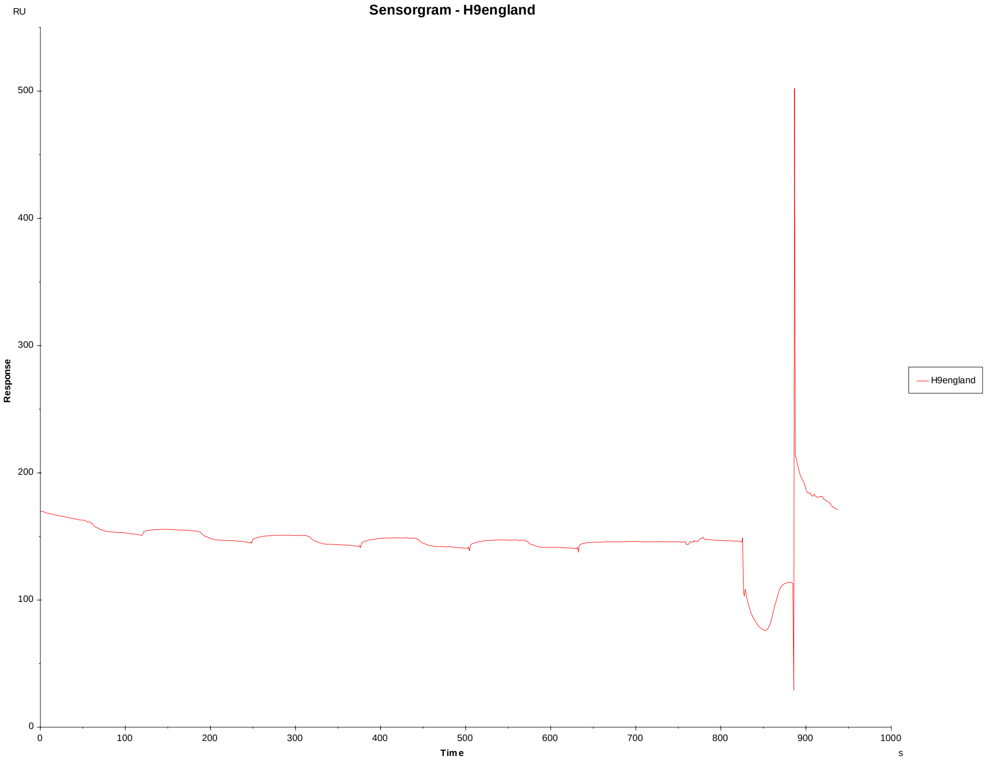

Figure S28. Single-cycle kinetic analysis of the interaction between immobilized peptide **S3** and **H9** by SPR in which no binding was observed. Response was measured at 5 different protein concentrations (3 nM, 6 nM, 12 nM, 25 nM and 50 nM).

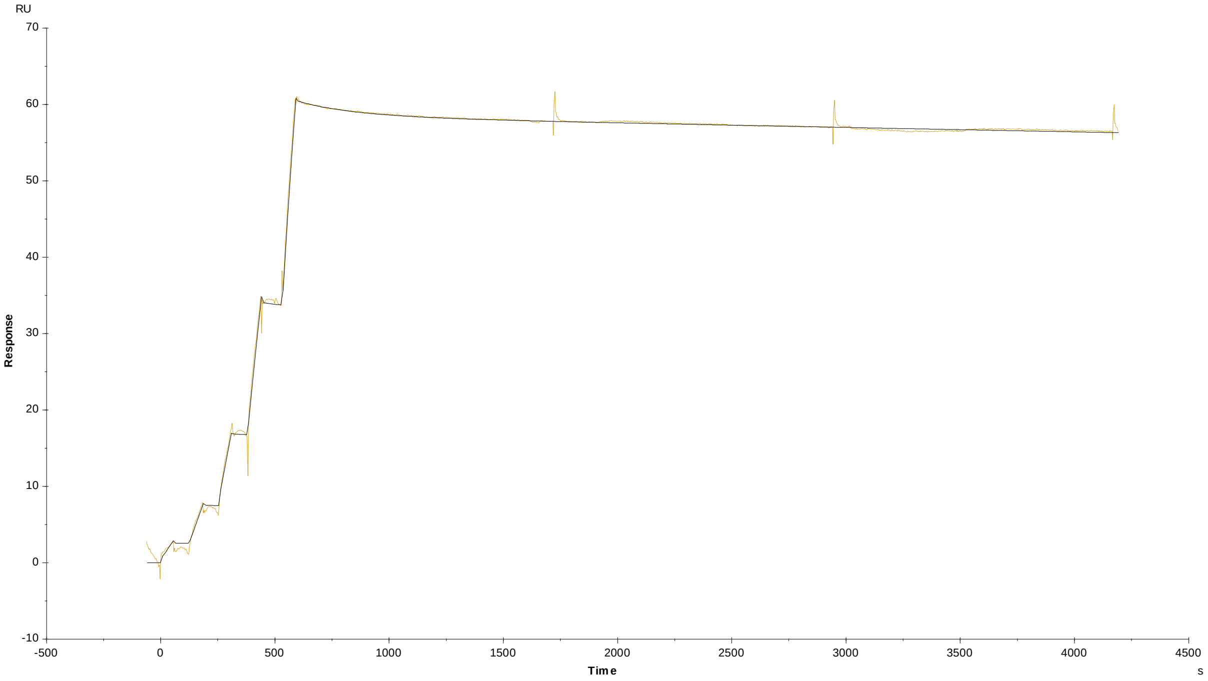

Figure S29. Single-cycle kinetic analysis of the interaction between immobilized peptide **S5** and **H1** by SPR, curve fitting with bivalent analyte model. Response was measured at 5 different protein concentrations (3 nM, 6 nM, 12 nM, 25 nM and 50 nM).

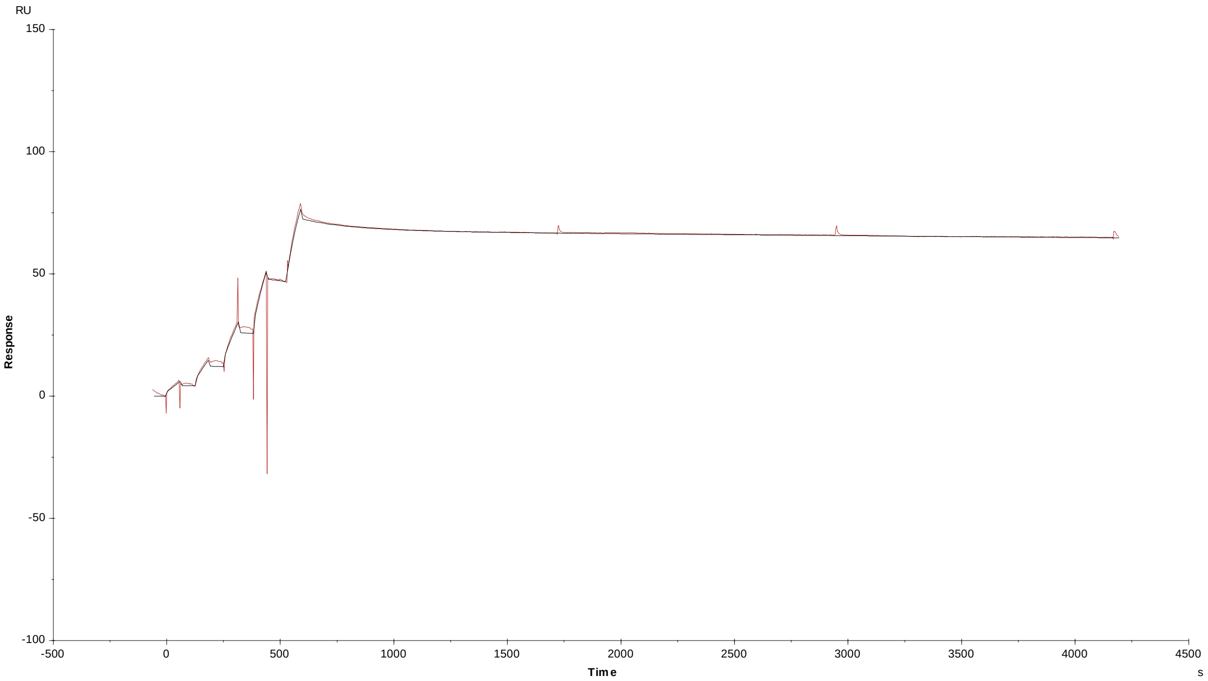

Figure S30. Single-cycle kinetic analysis of the interaction between immobilized peptide **S5** and **H1stem** by SPR, curve fitting with bivalent analyte model. Response was measured at 5 different protein concentrations (3 nM, 6 nM, 12 nM, 25 nM and 50 nM).

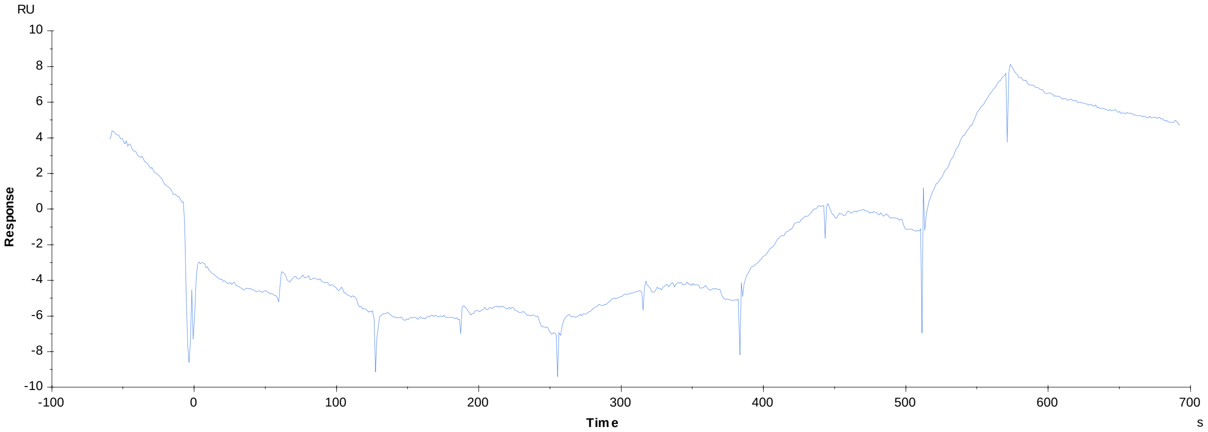

Figure S31. Single-cycle kinetic analysis of the interaction between immobilized peptide **S5** and **H1stem I375F** by SPR in which no binding was observed. Response was measured at 5 different protein concentrations (6 nM, 12 nM, 25 nM, 50 nM and 100 nM).

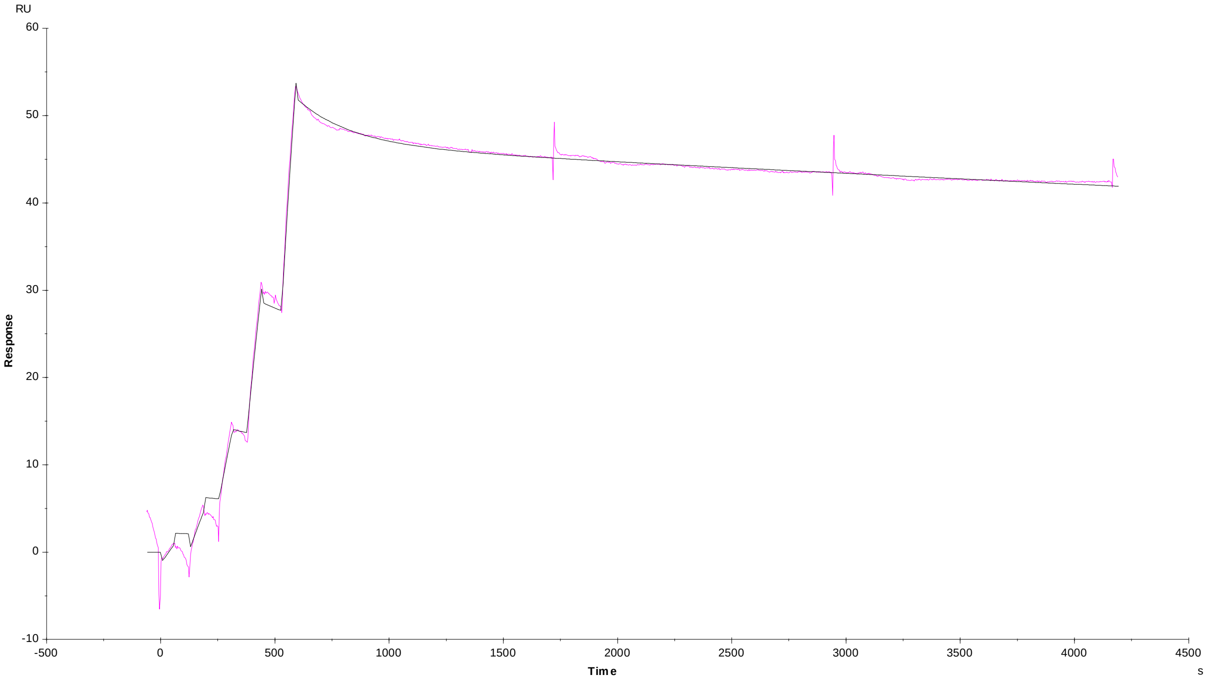

Figure S32. Single-cycle kinetic analysis of the interaction between immobilized peptide **S5** and **H1stem I463V** by SPR, curve fitting with bivalent analyte model. Response was measured at 5 different protein concentrations (3 nM, 6 nM, 12 nM, 25 nM and 50 nM).

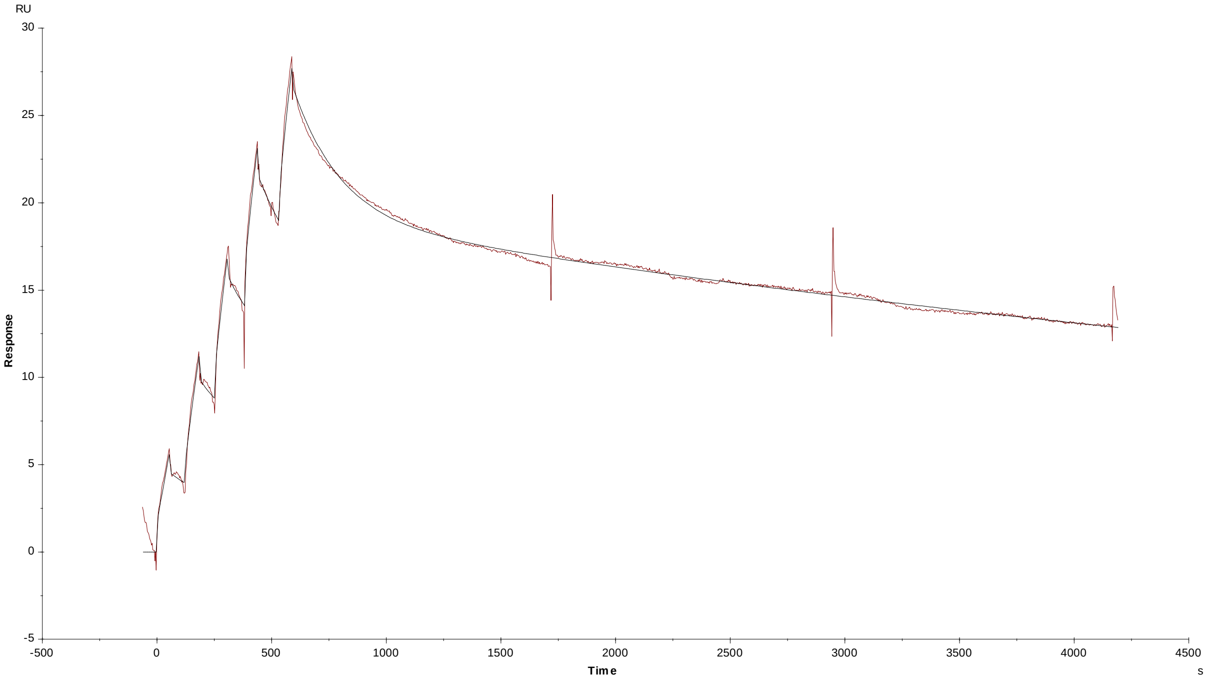

Figure S33. Single-cycle kinetic analysis of the interaction between immobilized peptide **S5** and **H5stem** by SPR, curve fitting with bivalent analyte model. Response was measured at 5 different protein concentrations (20 nM, 30 nM, 40 nM, 50 nM and 60 nM).

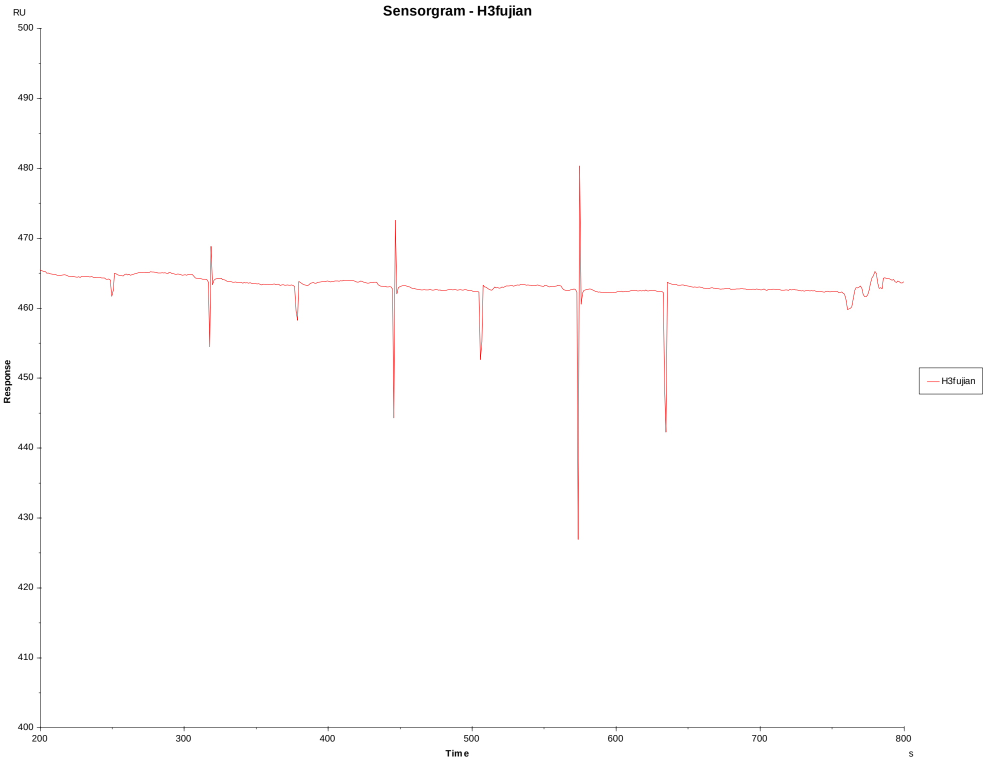

Figure S34. Single-cycle kinetic analysis of the interaction between immobilized peptide **S5** and **H3** by SPR in which no binding was observed. Response was measured at 5 different protein concentrations (6 nM, 12 nM, 25 nM, 50 nM and 100 nM).

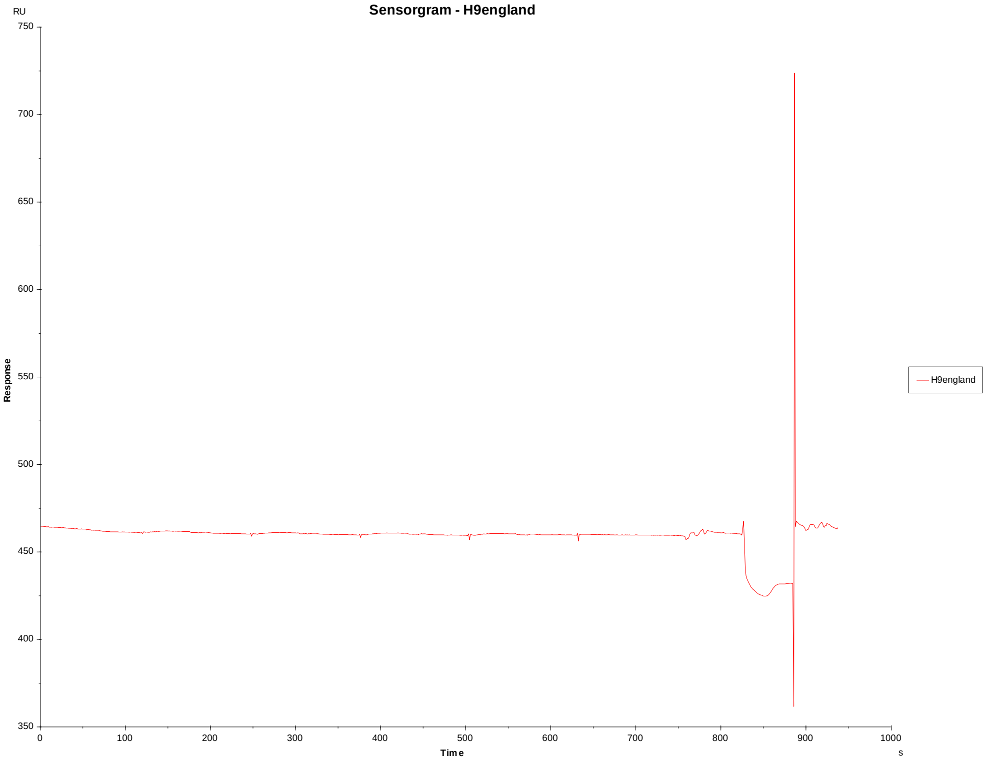

Figure S35. Single-cycle kinetic analysis of the interaction between immobilized peptide **S5** and **H9** by SPR in which no binding was observed. Response was measured at 5 different protein concentrations (3 nM, 6 nM, 12 nM, 25 nM and 50 nM).

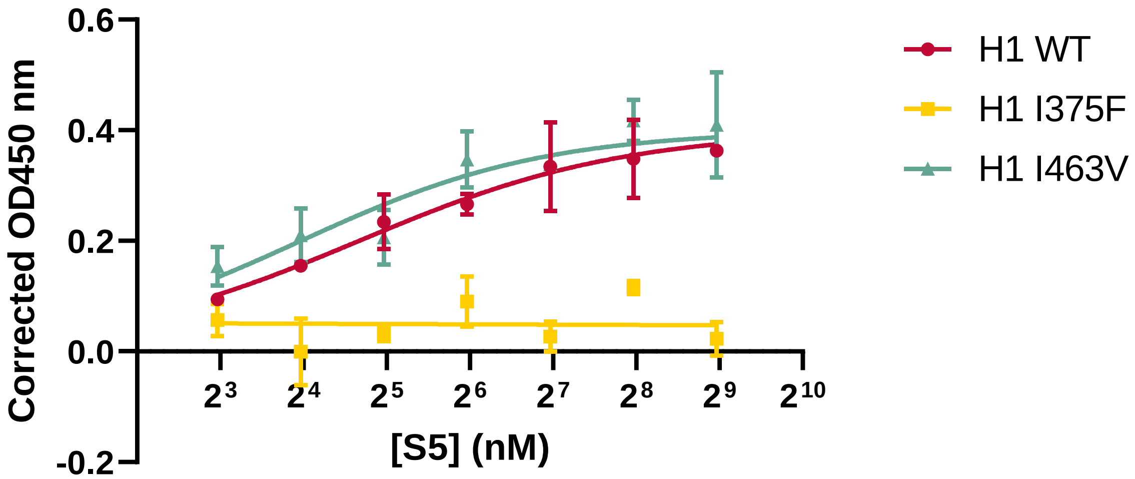

Figure S36. I375F mutation hinders binding of S5 to H1. Binding of S5 to cell surface-expressed wild-type H1 and H1 containing either the I375F or I463V substitution was analysed in a cell-based ELISA.

```
                                                     369   375         387
H1pdm09     EGGWTGMVDGWYGYHHQNEQGSGYAADLKSTQNAIDEITNKVNSVIEKMNTQFTAVGKEFN   400
H1pr8       EGGWTGMIDGWYGYHHQNEQGSGYAADQKSTQNAINGITNKVNTVIEKMNIQFTAVGKEFN   400
H1stem      EGGWTGMVDGWYGYHHQNEQGSGYAADQKSTQNAINGITNKVNSVIEKMNTQYTAIGCEYN   400
H5          EGGWQGMVDGWYGYHHSNEQGSGYAADKESTQKAIDGVTNKVNSIIDKMNTQFEAVGREFN   400
H5stem      EGGWQGMVDGWYGYHHSNEQGSGYAADKESTQKAIDGVTNKVNSIIDKMNTQYEAIGCEYN   400
H9          EGGWPGLVAGWYGFQHSNDQGVGMAADRESTQKAVDKITSKVNNIIDKMNKQYEIIDHEFS   400
H3_FU02     ENGWEGMVDGWYGFRHQNSEGTGQAADLKSTQAAINQINGKLNRLIGKTNEKFHQIEKEFS   400
H3_VI75     ENGWEGMIDGWYGFRHQNSEGTGQAADLKSTQAAIDQINGKLNRVIEKTNEKFHQIEKEFS   400
```

Figure S37. Alignment of macrocyclic peptide binding site in IAV HA. Alignment of part of different IAV HAs, containing the binding site of several macrocyclic peptides (colored yellow) as determined by HDX analysis, is shown for A/Netherlands/602/2009 H1N1 and A/California/04/2009 (both indicated by H1pdm09 as they are identical in the sequence shown), A/Puerto Rico/8/34/Mount Sinai H1N1(H1pr8), A/Vietnam/1194/2004 H5N1 (H5), A/turkey/England/13437/2013 H9N2 (H9), A/Fujian/411/2002 H3N2 (H3_FU02), A/Bilthoven/1761/76 (H3_VI75) and of HAstem proteins designed according to Impagliazzo et al.[7] for A/Kentucky/UR06-0258/2007 H1N1 (H1stem) and A/Vietnam/1194/2004 H5N1 (H5stem). H3 numbering is indicated. Isoleucine at position 375, substitution of which (I375F) interferes with peptide binding, is colored light blue. Amino acids differing in the peptide binding site from the sequence of H1pdm09 are colored grey.

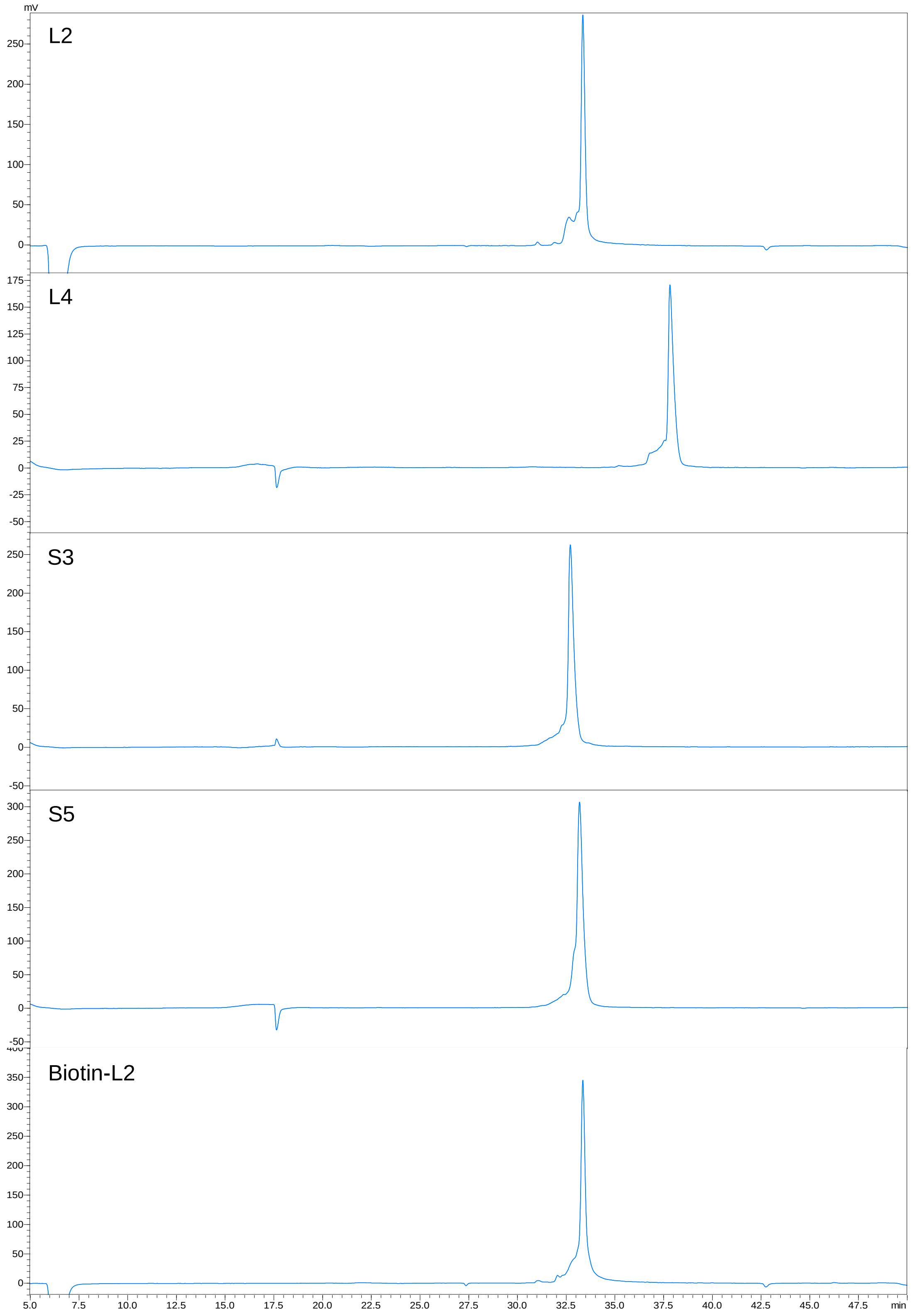
mV
L2
L4
S3
S5
Biotin-L2
min

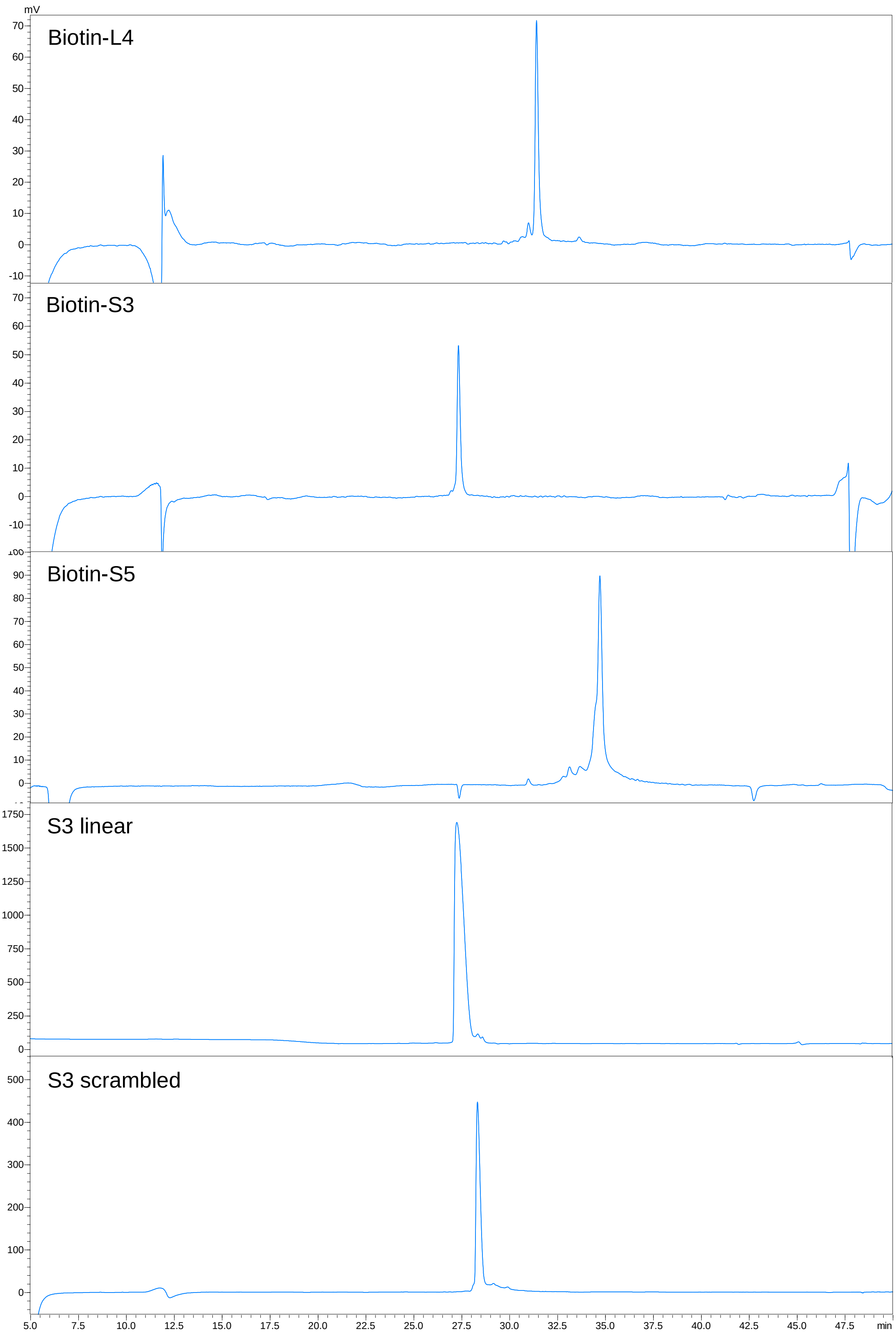

mV
Biotin-L4
Biotin-S3
Biotin-S5
S3 linear
S3 scrambled
min

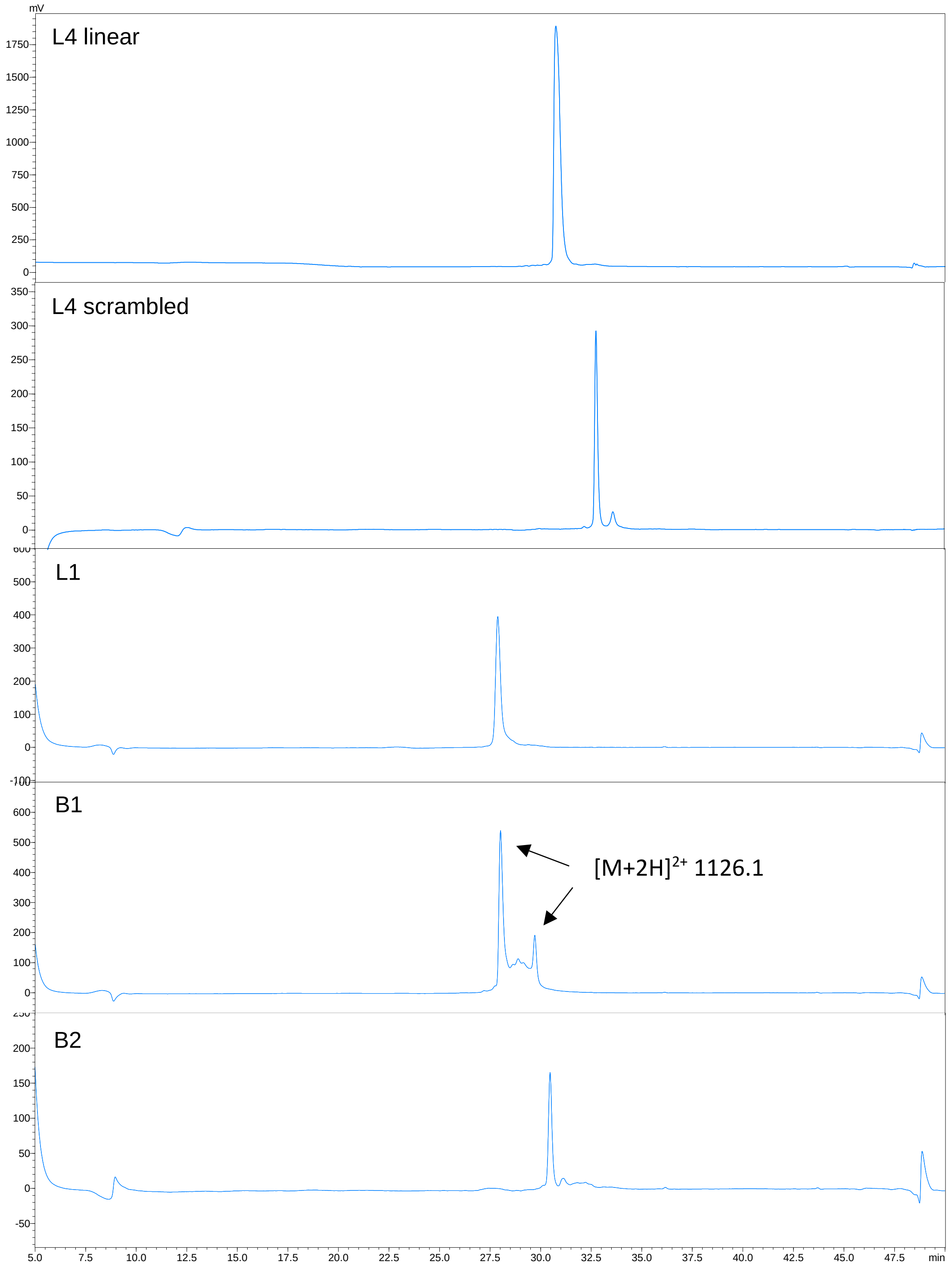
mV
L4 linear
L4 scrambled
L1
B1
[M+2H]$^{2+}$ 1126.1
B2
min

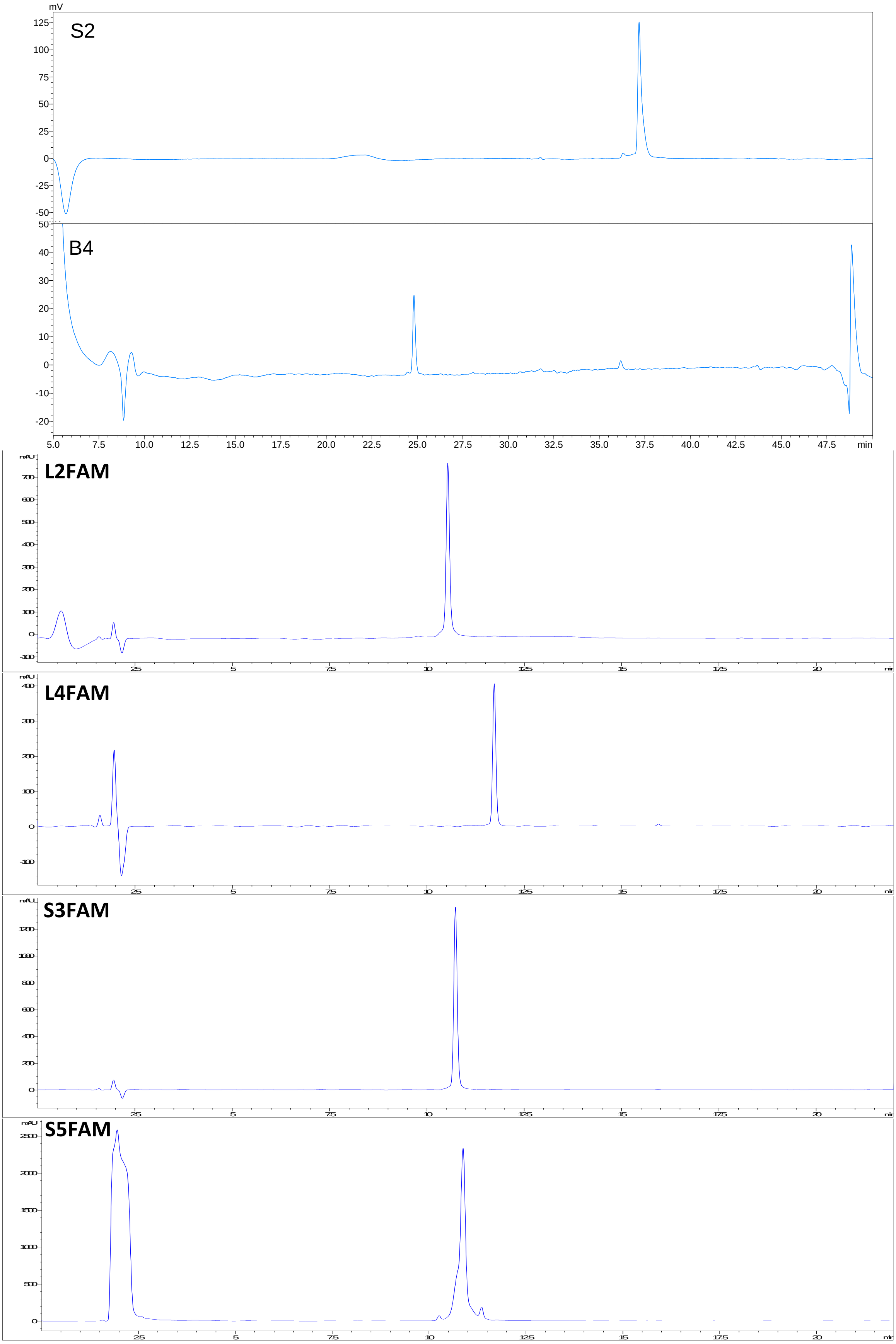

Figure S38. HPLC analysis of purified peptides. UV-absorption traces at 215 nm.

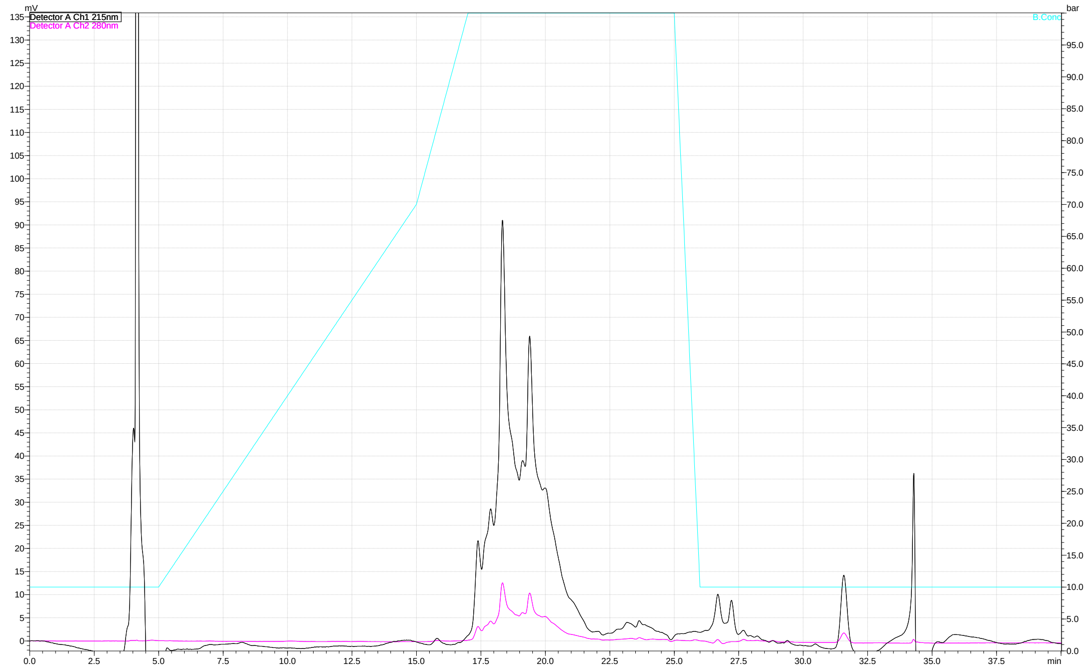

Figure S39. HPLC analysis of crude crude peptide **S1**. Correct peptide mass was not detected. (black, A215; purple A280; cyan, gradient)

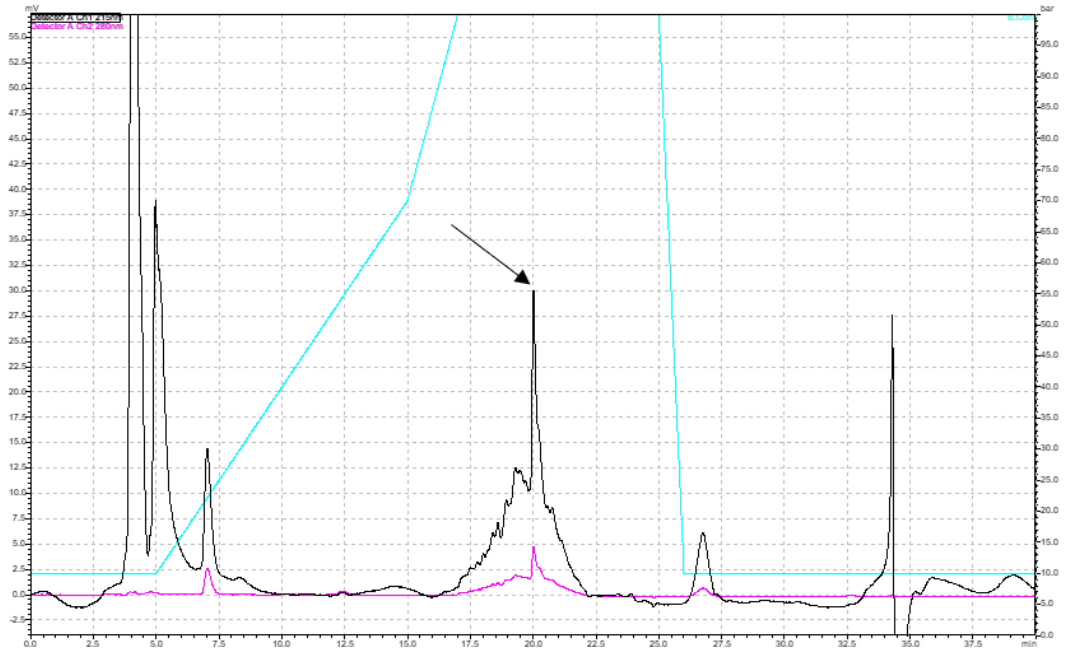

Figure S40. HPLC analysis of crude peptide **S2**. Peak corresponding to the correct peptide mass is highlighted by the arrow. (black, A215; purple A280; cyan, gradient)

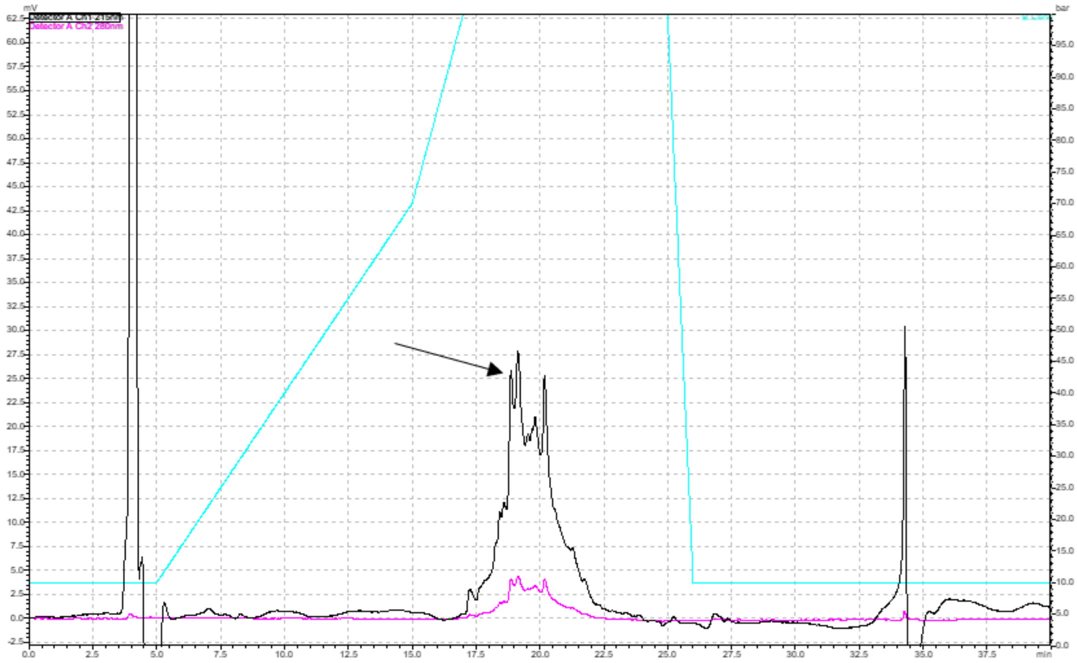

Figure S41. HPLC analysis of crude peptide **S3**. Peak corresponding to the correct peptide mass is highlighted by the arrow. (black, A215; purple A280; cyan, gradient)

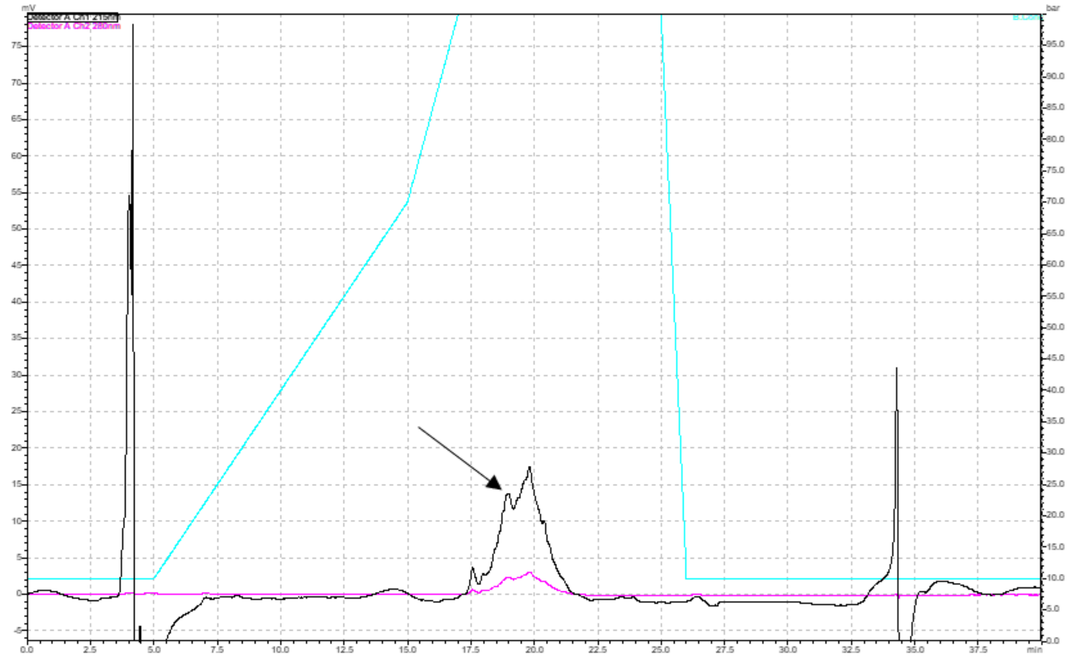

Figure S42. HPLC analysis of crude peptide **S4**. Peak corresponding to the correct peptide mass is highlighted by the arrow. (black, A215; purple A280; cyan, gradient)

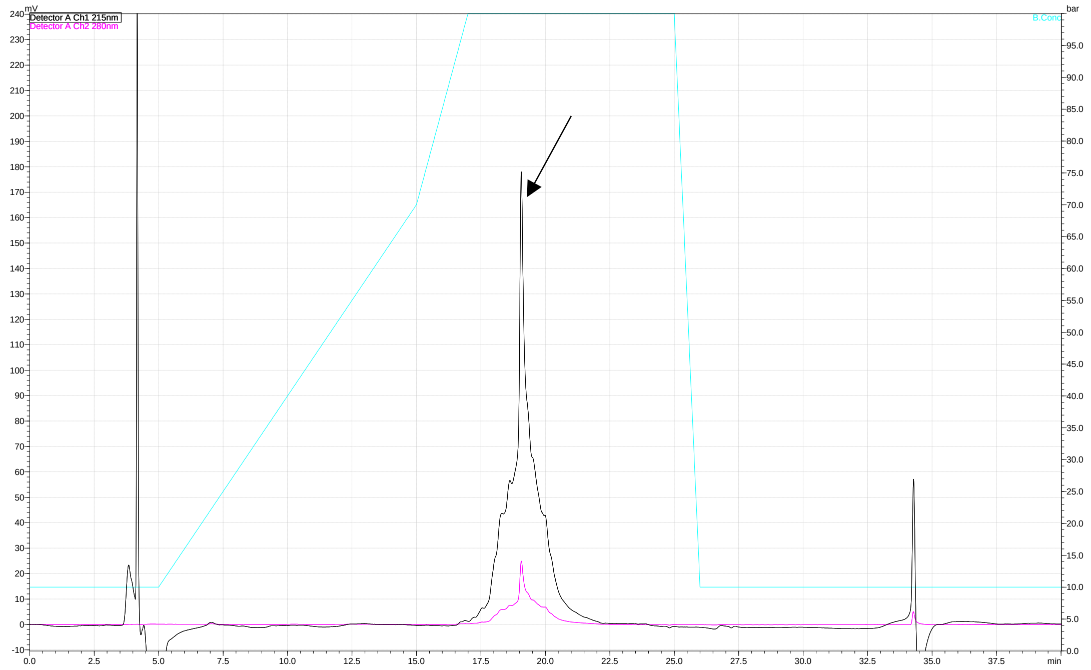

Figure S43. HPLC analysis of crude peptide **S5**. Peak corresponding to the correct peptide mass is highlighted by the arrow. (black, A215; purple A280; cyan, gradient)

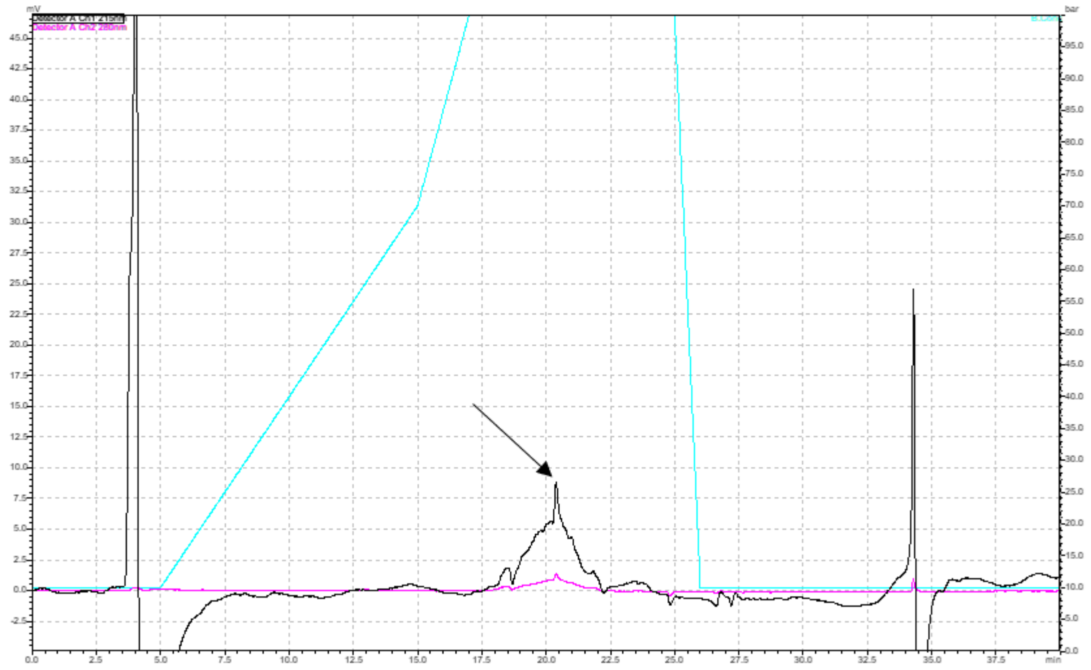

Figure S44. HPLC analysis of crude peptide **S6**. Peak corresponding to the correct peptide mass is highlighted by the arrow. (black, A215; purple A280; cyan, gradient)

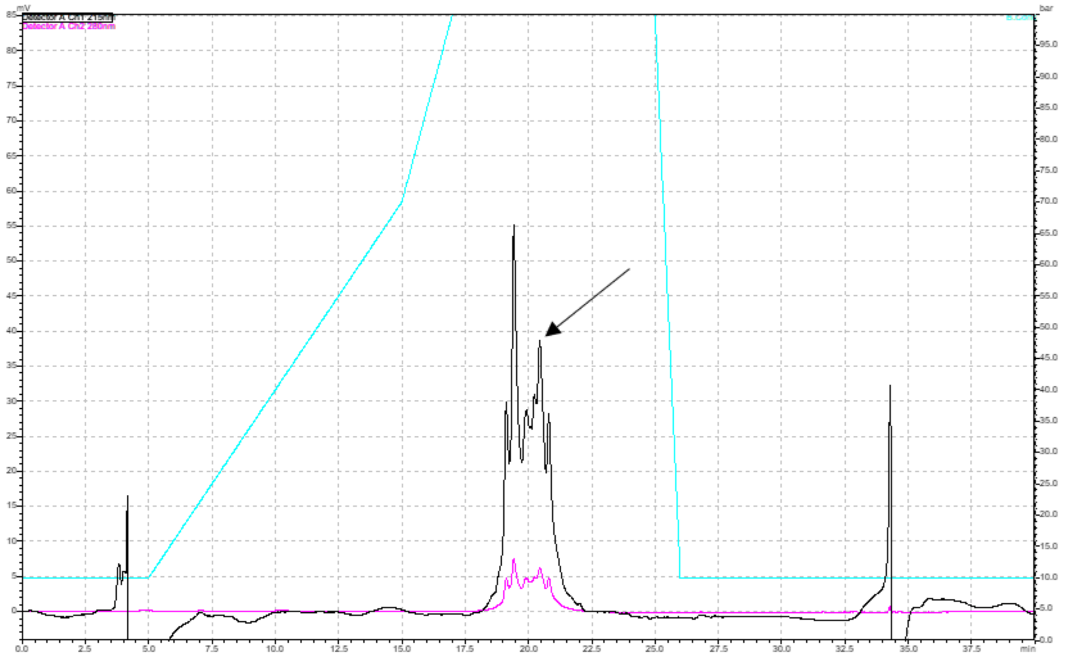

Figure S45. HPLC analysis of crude peptide **S7**. Peak corresponding to the correct peptide mass is highlighted by the arrow. (black, A215; purple A280; cyan, gradient)

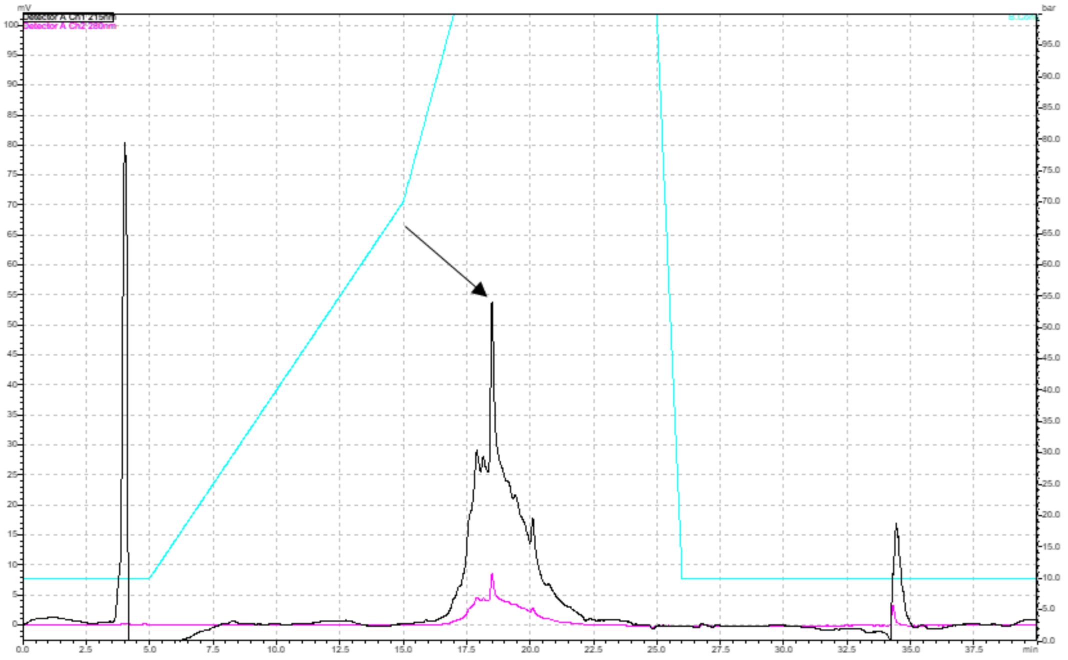

Figure S46. HPLC analysis of crude peptide **S8**. Peak corresponding to the correct peptide mass is highlighted by the arrow. (black, A215; purple A280; cyan, gradient)

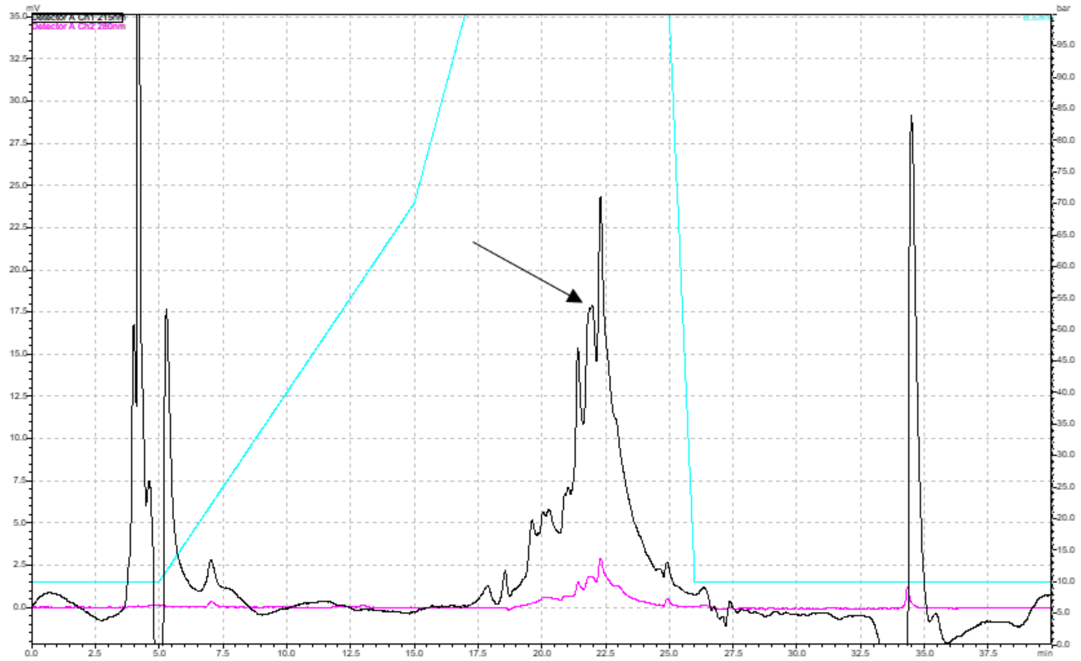

Figure S47. HPLC analysis of crude peptide **S9**. Peak corresponding to the correct peptide mass is highlighted by the arrow. (black, A215; purple A280; cyan, gradient)

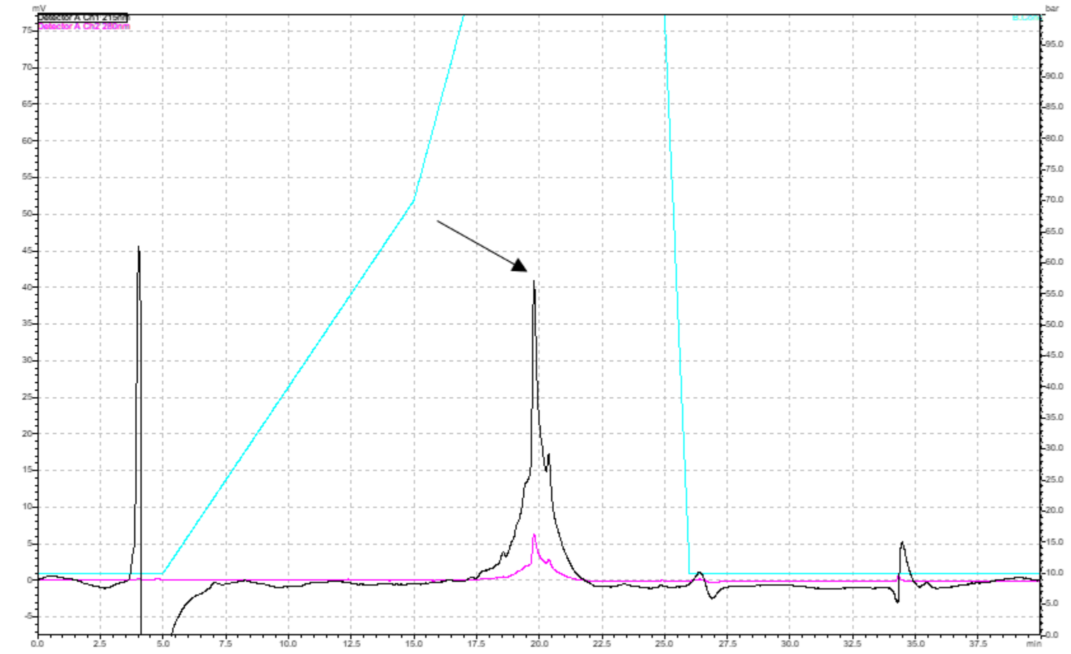

Figure S48. HPLC analysis of crude peptide **B1**. Peak corresponding to the correct peptide mass is highlighted by the arrow. (black, A215; purple A280; cyan, gradient)

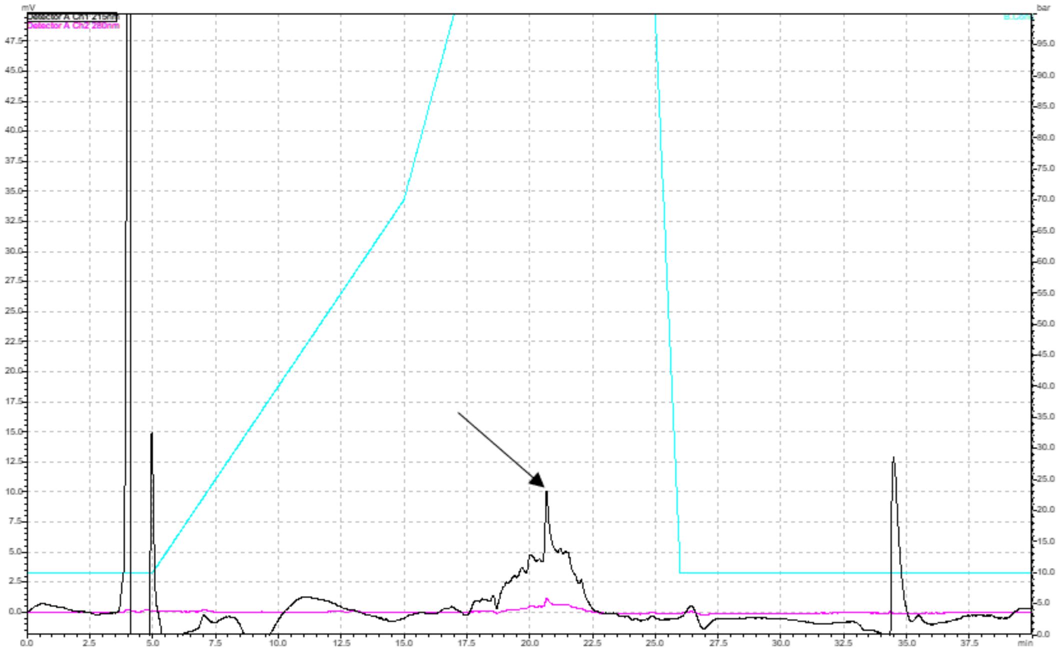

Figure S49. HPLC analysis of crude peptide **B2**. Peak corresponding to the correct peptide mass is highlighted by the arrow. (black, A215; purple A280; cyan, gradient)

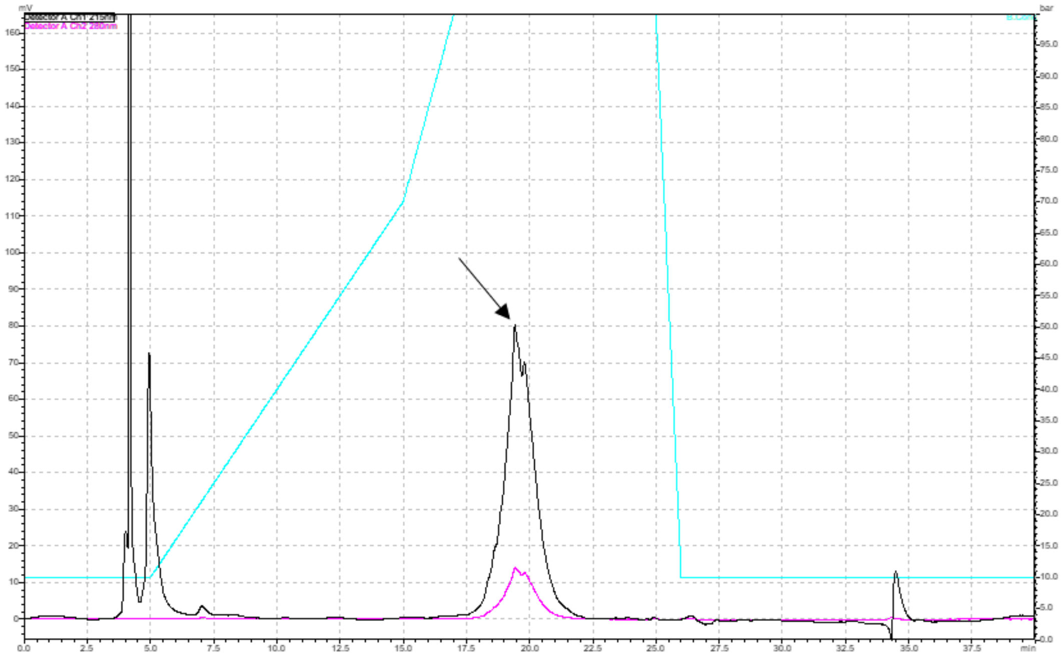

Figure S50. HPLC analysis of crude peptide **B3**. Peak corresponding to the correct peptide mass is highlighted by the arrow. (black, A215; purple A280; cyan, gradient)

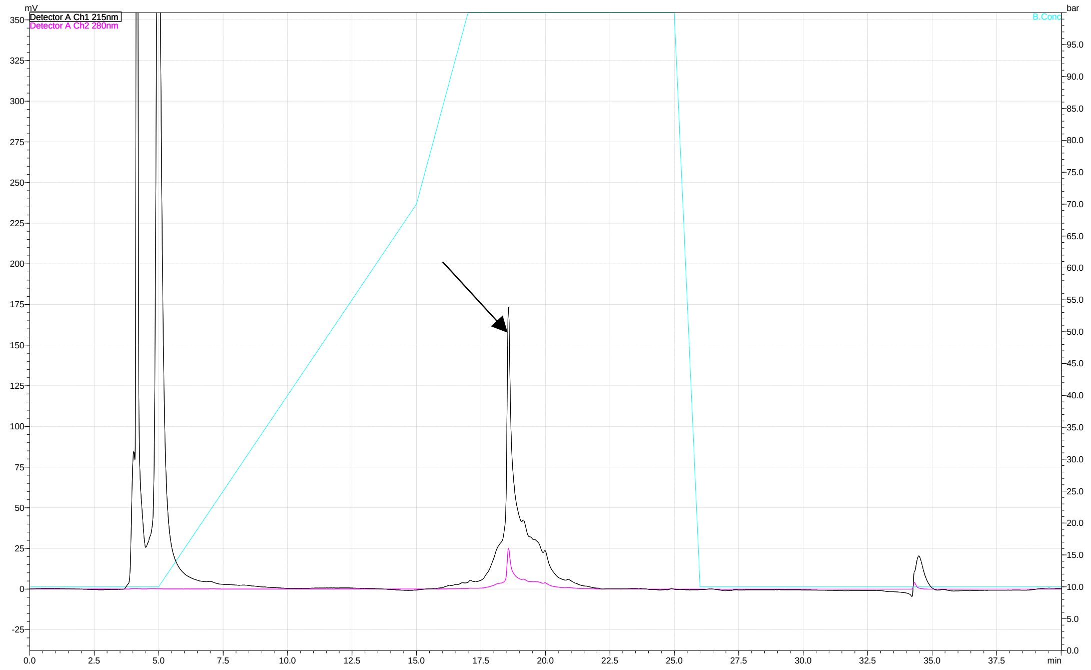

Figure S51. HPLC analysis of crude peptide **B4**. Peak corresponding to the correct peptide mass is highlighted by the arrow. (black, A215; purple A280; cyan, gradient)

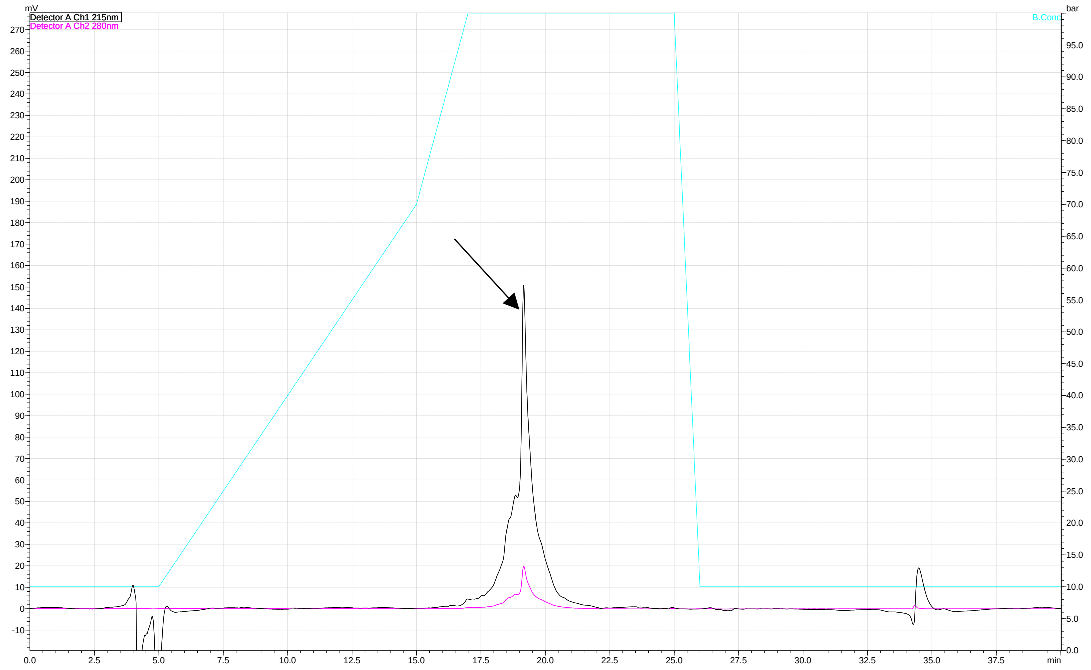

Figure S52. HPLC analysis of crude peptide **B5**. Peak corresponding to the correct peptide mass is highlighted by the arrow. (black, A215; purple A280; cyan, gradient)

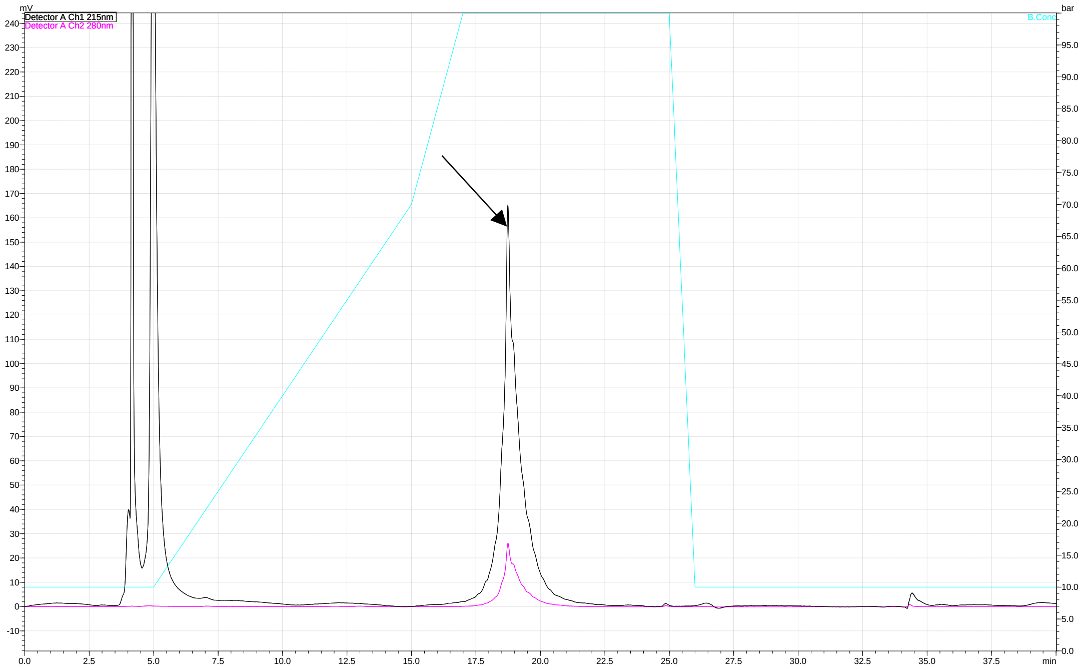

Figure S53. HPLC analysis of crude peptide **B6**. Peak corresponding to the correct peptide mass is highlighted by the arrow. (black, A215; purple A280; cyan, gradient)

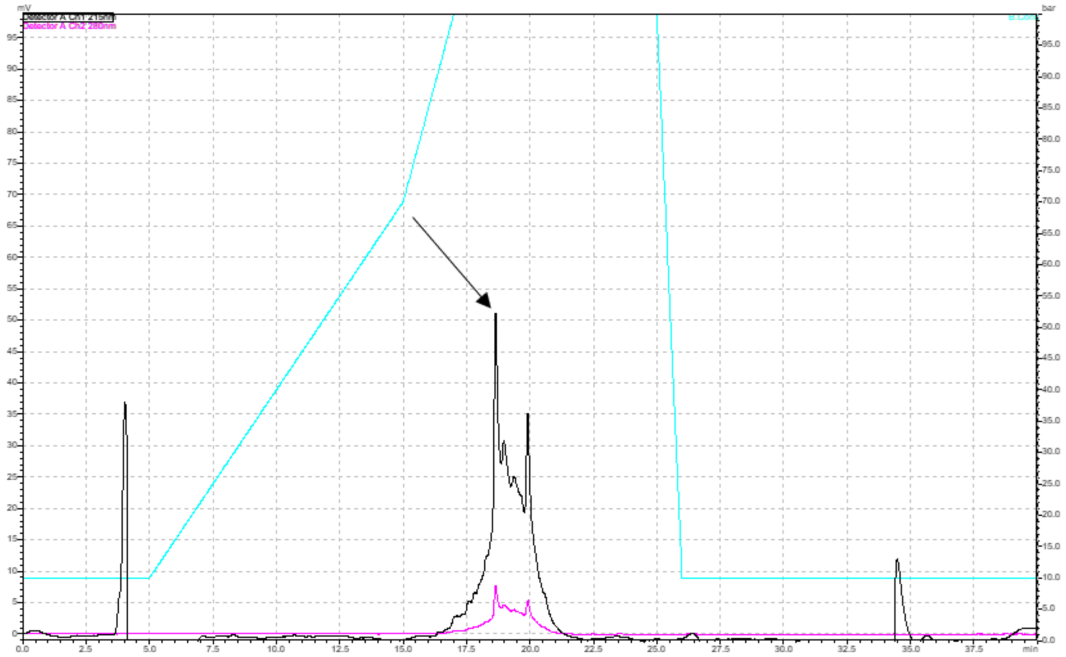

Figure S54. HPLC analysis of crude peptide **B8**. Peak corresponding to the correct peptide mass is highlighted by the arrow. (black, A215; purple A280; cyan, gradient)

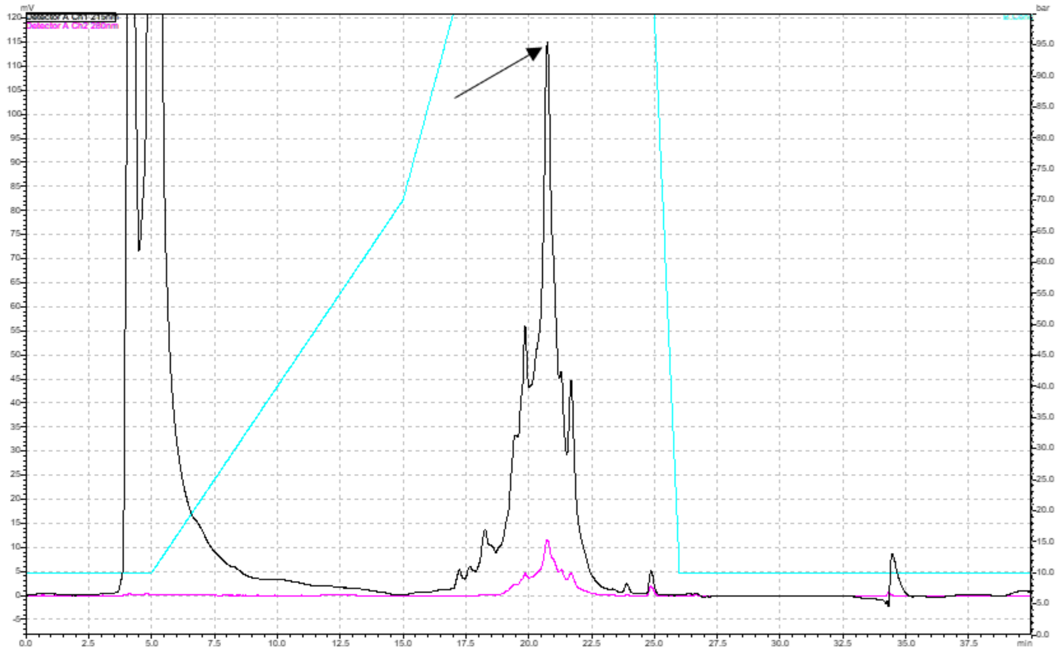

Figure S55. HPLC analysis of crude peptide **B9**. Peak corresponding to the correct peptide mass is highlighted by the arrow. (black, A215; purple A280; cyan, gradient)

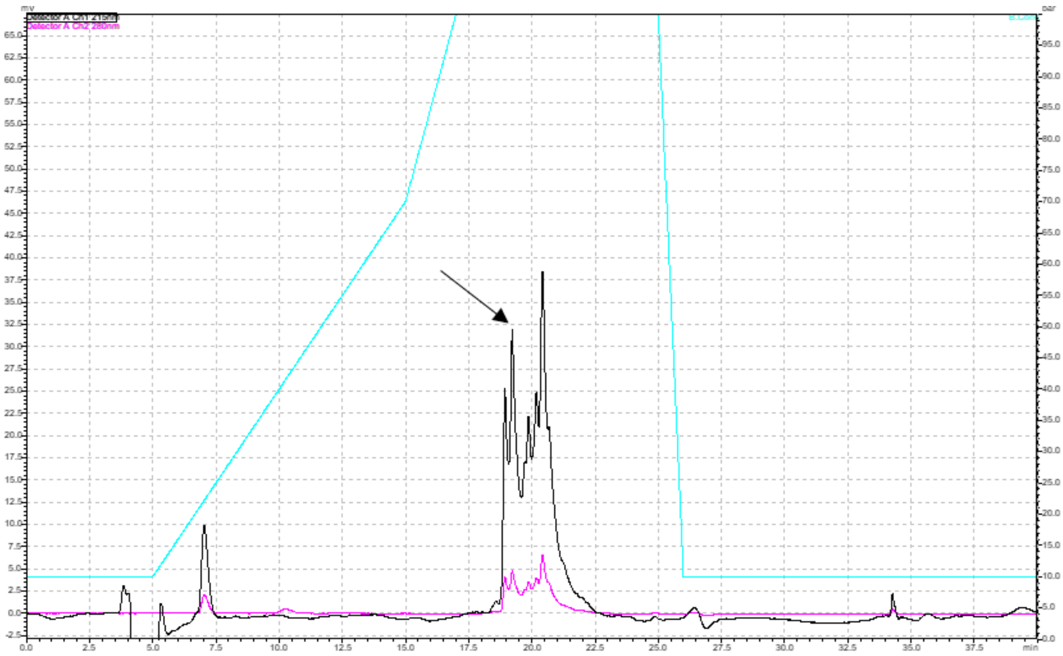

Figure S56. HPLC analysis of crude peptide **R1**. Peak corresponding to the correct peptide mass is highlighted by the arrow. (black, A215; purple A280; cyan, gradient)

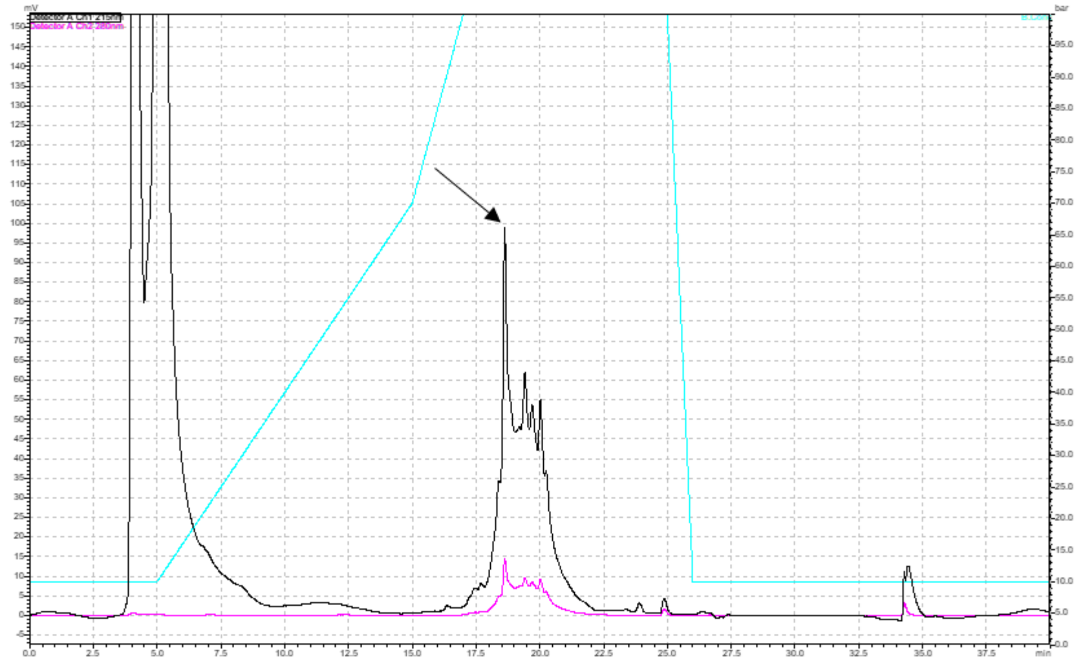

Figure S57. HPLC analysis of crude peptide **R2**. Peak corresponding to the correct peptide mass is highlighted by the arrow. (black, A215; purple A280; cyan, gradient)

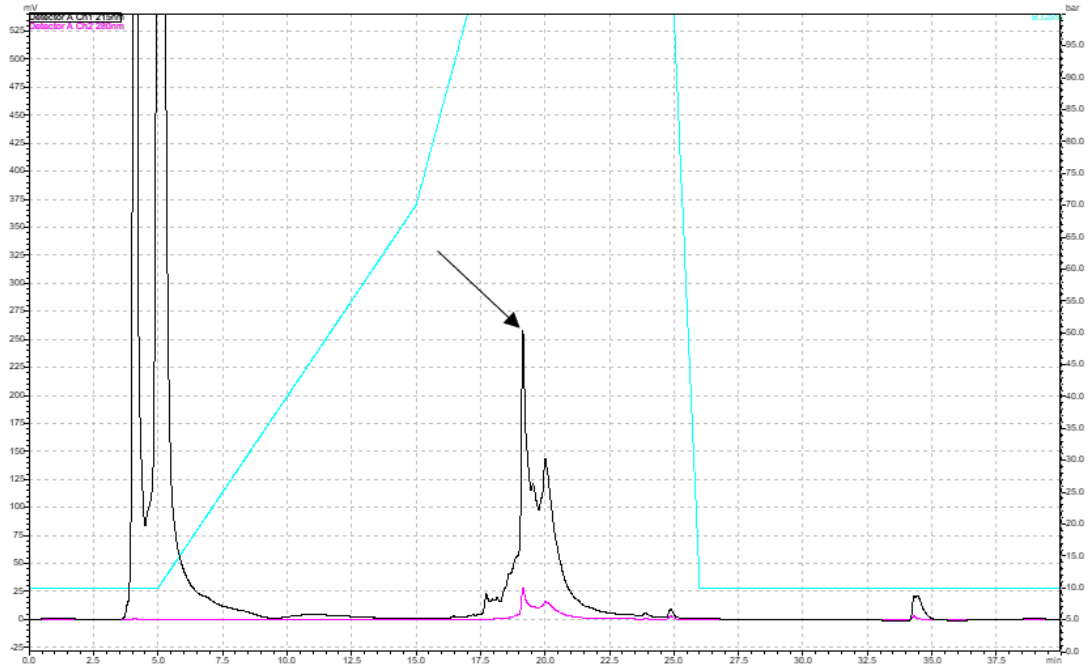

Figure S58. HPLC analysis of crude peptide **R4**. Peak corresponding to the correct peptide mass is highlighted by the arrow. (black, A215; purple A280; cyan, gradient)

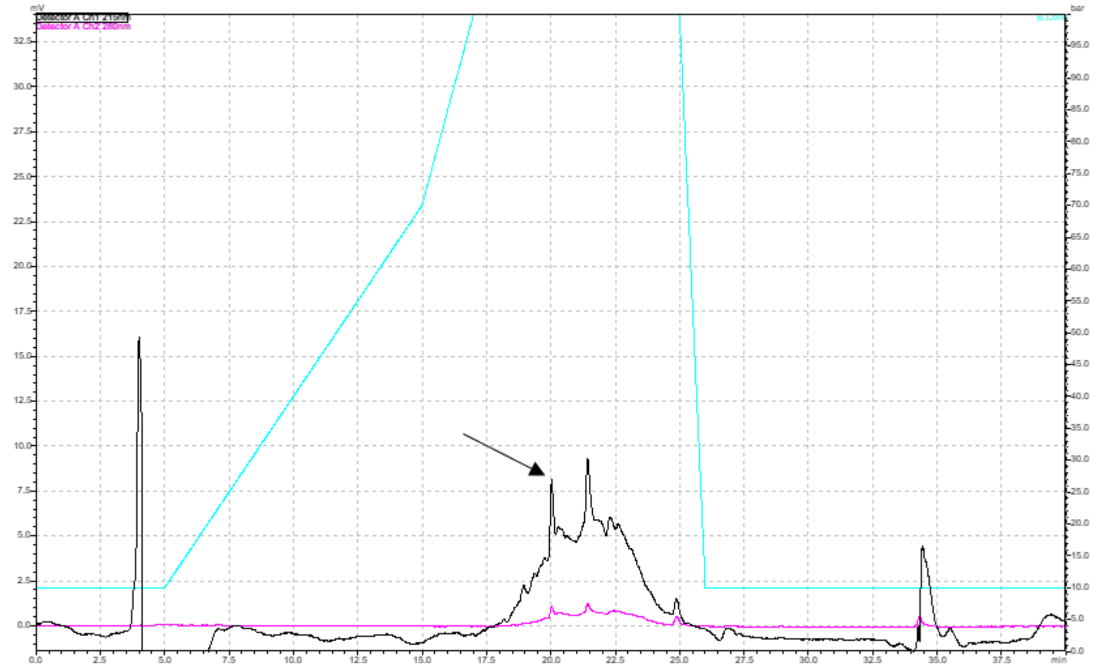

Figure S59. HPLC analysis of crude peptide **R5**. Peak corresponding to the correct peptide mass is highlighted by the arrow. (black, A215; purple A280; cyan, gradient)

Supplementary references

1. Yoshisada, R., van Gijzel, L. & Jongkees, S. A. K. (2017) Towards Tuneable Retaining Glycosidase-Inhibiting Peptides by Mimicry of a Plant Flavonol Warhead. *ChemBioChem* 18 (23), 2333–2339.

2. Pícha, J., Buděšínský, M., Macháčková, K., Collinsová, M. & Jiráček, J. (2017) Optimized syntheses of Fmoc azido amino acids for the preparation of azidopeptides. *J. Pept. Sci.* 23 (3), 202–214.

3. Guo, H., Rabouw, H., Slomp, A., Dai, M., van der Vegt, F., van Lent, J. W. M., McBride, R., Paulson, J. C., de Groot, R. J., van Kuppeveld, F. J. M. *et al.* (2018) Kinetic analysis of the influenza A virus HA/NA balance reveals contribution of NA to virus-receptor binding and NA-dependent rolling on receptor-containing surfaces. *PLOS Pathog.* 14 (8), e100723.

4. Munster, V. J., de Wit, E., van den Brand, J. M. A., Herfst, S., Schrauwen, E. J. A., Besteboer, T. M., van de Vijver, D., Boucher, C. A., Koopmans, M., Rimmelzwaan, G. f. *et al.* (2009) Pathogenesis and Transmission of Swine-Origin 2009 A(H1N1) Influenza Virus in Ferrets. *Science* 325 (5939), 481–483.

5. Du, W., Dai, M., Li, Z., Boons, G., Peeters, B., van Kuppeveld, F. J. M., de Vries, E., de Haan, C. A. M. (2018) Substrate Binding by the Second Sialic Acid-Binding Site of Influenza A Virus N1 Neuraminidase Contributes to Enzymatic Activity. *J. Virol.* 92 (20), e01243-18.

6. Koel, B. F., Burke, D. F., Bestebroer, T. M., van der Vliet, S., Zondag, G. C. M., Vervaet, G., Skepner, E., Lewis, N. S., Spronken, M. I. J., Russel, C. A. *et al.* (2013) Substitutions Near the Receptor Binding Site Determine Major Antigenic Change During Influenza Virus Evolution. *Science* 342 (6161), 976–979.

7. Impagliazzo, A. , Milder, F., Kuipers, H., Wagner, M. V., Zhu, X., Hoffman, R. M. B., van Meersbergen, R., Huizingh, J., Wanningen, P., Verspuij, J. *et al.* (2015) A stable trimeric influenza hemagglutinin stem as a broadly protective immunogen. *Science* 349 (6254), 1301–1306.
